# *Salvia limbata* extracts and essential oil as a novel source of bioactive agents: metabolomic fingerprint, *in vitro*, *in silico* and network pharmacological perspectives

**DOI:** 10.1039/d6ra05137e

**Published:** 2026-09-14

**Authors:** Bengusu H. Akgul, Dimitrina Zheleva-Dimitrova, Łukasz Swiątek, Mehmet Veysi Cetiz, Abdullahi Ibrahim Uba, Nilofar Nilofar, Mehmet Maruf Balos, Ismail Yapıcı, Ilhami Gulcin, Gokhan Zengin

**Affiliations:** a Department of Biology, Science Faculty, Selcuk University 42130 Konya Turkey gokhanzengin@selcuk.edu.tr; b Department of Pharmacognosy, Faculty of Pharmacy, Medical University 1000 Sofia Bulgaria dzheleva@pharmfac.mu-sofia.bg; c Department of Virology with Viral Diagnostics Laboratory, Medical University of Lublin Poland; d Department of Medical Biochemistry, Faculty of Medicine, Harran University Sanliurfa 63290 Turkey; e Department of Biostatistics and Medical Informatics, Faculty of Medicine, Istinye University 34396 Istanbul Türkiye; f Botanic Garden “Giardino dei Semplici”, Department of Pharmacy, “G. d'Annunzio” University 66100 Chieti Italy; g Şanlıurfa Provincial Directorate of National Education, Mehmet Gunes Anatolian High School TR-63000 Haliliye Şanlıurfa Turkey; h Department of Chemistry, Science Faculty, Ataturk University Erzurum Turkey

## Abstract

The genus *Salvia* has received considerable attention and has been studied extensively owing to its rich polyphenolic content. In the present study, *Salvia limbata* essential oil (EO) and extracts from its aerial parts and roots were used to examine the chemical profile and biological activities. The chemical profiles of the EO and extracts were determined using GC-MS and UHPLC-HRMS analyses, respectively. The biological activities were assessed using six antioxidant assays, nine enzyme inhibitions, and four cell lines for cytotoxicity and antiviral properties. In the essential oils, 8,13-epoxy-15,16-dinorlabd-12-ene (16.87%) and phytol (7.84%) were the dominant compounds. UHPLC-HRMS revealed that flavonoids were the main groups in the tested extracts, as well as phenolic acids (hydroxycinnamic and hydroxybenzoic acids) and caffeic acid oligomers. Among the extracts, the ethyl acetate (EA) root extract had the highest antioxidant activity, with values of 503.32 mg TE per g, 425.44 mg TE per g, and 349.34 mg TE per g in the CUPRAC, ABTS, and FRAP assays, respectively. The aerial-part ethanol/water extract showed higher antioxidant capacity in the CUPRAC, ABTS, and DPPH assays. The EO showed strong enzyme inhibition, notably against AChE, BChE, tyrosinase, and lipase, and exhibited significantly higher inhibitory activity against CA I (IC_50_ = 10.93 µg mL^−1^) and CA II (IC_50_ = 27.07 µg mL^−1^) compared to all solvent extracts. The aerial-part hexane, ethyl acetate, and ethanolic extracts of *S. limbata* were moderately cytotoxic to non-cancerous and all cancer-derived cell lines, while the extract obtained from the leaves with 70% ethanol was not toxic to non-cancerous VERO cells but showed weak to moderate cytotoxicity to the other cell lines. Ethanolic and aqueous ethanolic aerial-part extracts, as well as the EO, reduced the HHV-1-induced cytopathic effect, but the titration assay indicated that the highest inhibition of virus infectivity was found for the aqueous ethanolic extract. To gain further insights into the molecular mechanisms underlying these biological activities, major identified metabolites were evaluated using network pharmacology, molecular docking, and molecular dynamics simulations. These results emphasize the significant medicinal value of *S. limbata*, which exhibits promising antioxidant, enzyme-inhibiting, and anticancer activities.

## Abstract 2

*Salvia limbata* is a promising source of multifunctional bioactive agents for preparing effective health-promoting applications.

## Graphical abstract

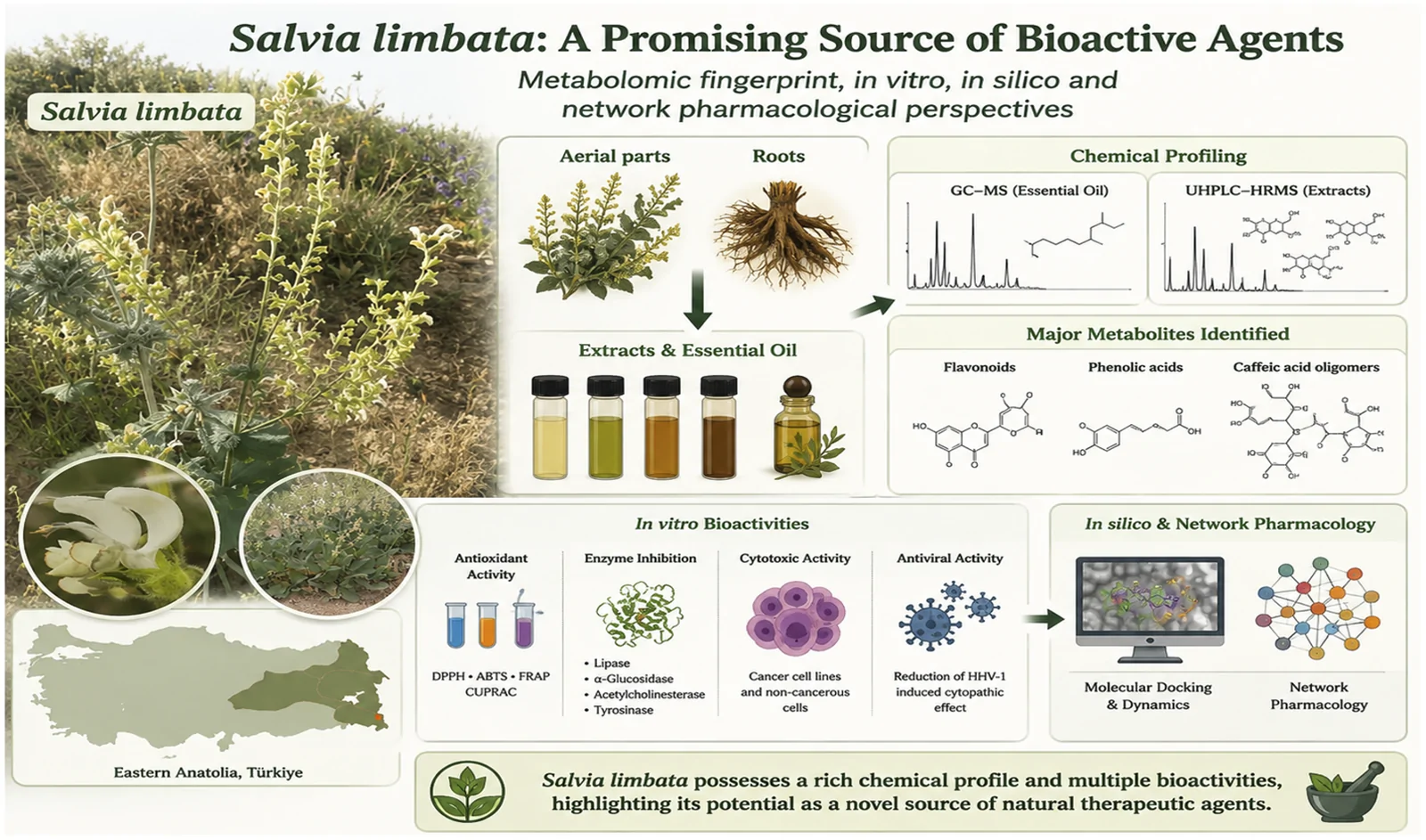

## 1. Introduction

*Salvia* is a diverse genus comprising around 1000 species, belonging to the Lamiaceae family.^1^ In Turkey, the *Salvia* genus includes 115 species, of which 63 species (54.7%) are endemic. Because of the wide variety of natural compounds found in the species of this genus, the *Salvia* species have been used for treating gastrointestinal and respiratory problems, infections, and other diseases, such as liver problems, diabetes, infertility, and hemorrhaging, in Middle Eastern traditional medicine.^2^

The aerial parts of many *Salvia* species have been used to flavor food items and in folk medicine. The most well-known *Salvia* is *Salvia officinalis*,^3–5^ commonly referred to as sage, and other *Salvia* species, including *S. fruticosa*,^6^*S. lavandulifolia*,^7^ and *S. tomentosa*,^4^ have significant uses in producing essential oils, drugs, pigments, cosmetics, fragrances, and biocidal preparations. The *Salvia* genus has been reported to be used in European folk medicine to treat gastrointestinal disorders,^5^ inflammation in the mouth and throat,^8^ and skin abnormalities.^9^ However, in Asia^10–12^ and South America,^13,14^*Salvia* species have been reportedly employed to treat rheumatism, ulcers, diarrhea, and hyperglycemia.

The dried roots of *S. miltiorrhiza*, known as Chinese Danshen, have been traditionally used to treat disorders caused by the cardiovascular, neural, hepatic, and renal systems.^15,16^ Recent studies have indicated that the biologically active compounds of *Salvia* extracts, primarily polar phenolic acids, including salvianolic acids A, B, C, D, E, F, G, H, and K, as well as yunnaneic acids A, B, C, D, E, F, G, and H, primarily originate from the roots.^17–19^ Similarly, as reported by Llorent-Martínez *et al.*, caffeic acid derivatives are present in higher concentrations than flavonoids in many *Salvia* species.^20^ In South Africa, the indigenous *Salvia* species have been confirmed to contain phenolic compounds that are primarily responsible for their pharmacological properties.^21^ The bioactive compounds in *Salvia* include caffeic acid, oleanolic acid, ursolic acid, and, in some cases, alkaloids.^20,22,23^

The EO composition of *Salvia limbata* has been reported, and some studies have addressed its antimicrobial and antioxidant properties.^24^ However, comprehensive investigations of its root extract, particularly its antiviral, cytotoxic, and enzyme-inhibitory activities, remain limited. Furthermore, network pharmacology analysis was performed to identify the potential biological targets of the major metabolites, followed by molecular docking and dynamics simulations to evaluate the stability and binding behavior of selected protein–ligand complexes associated with cancer-related proteins, metabolic enzymes, carbonic anhydrase isoforms, and herpes virus targets. Network pharmacology enables the systematic identification of compound–target–pathway relationships, whereas molecular docking and dynamics simulations provide structural support for predicted compound–target interactions and their stability, respectively.^25,26^ Together, these complementary computational approaches facilitate the prioritization of bioactive compounds and therapeutic targets, providing a strong rationale for integrated natural product-based drug discovery. Therefore, the present study provides a more integrated evaluation of these biological activities (antioxidant, antiviral, cytotoxic, and enzyme-inhibitory), offering new insights into the pharmacological potential of this species.

## 2. Materials and methods

### 2.1. Plant collection

Plant specimens were collected in 2024 from Cilo Mount (Hakkari, Yüksekova, Mergan Valley) at an elevation of 2640 m. Dr Maruf Balos carried out the formal botanical classification of the material. A voucher specimen, under the code MB-5706, was placed in Harran University. After collection, the aerial parts and roots were separated immediately and air-dried at room temperature in a shaded, well-ventilated area. After complete drying, the plant material was milled to a fine powder. To ensure chemical integrity and prevent deterioration, the powdered samples were sealed in opaque containers and stored under stable conditions.

### 2.2. Plant extract preparation

Five solvent systems were used to obtain bioactive constituents: *n*-hexane, ethyl acetate, ethanol, 70% (v/v) ethanol–water, and distilled water. For each extraction, 10 g of the prepared plant material was mixed with 200 mL of the corresponding solvent. The methodology was adapted from our previous paper.^27^ 5 g of plant material was stirred (at almost 200 rpm using a magnetic stirrer) with the organic solvents (100 mL) for 24 h under ambient conditions (25 ± 2 °C). In contrast, in water-based extraction, the plant materials (5 g) were kept in boiled water (100 mL) for 15 min. After the extraction process, the mixture was filtered using a Whatman blue band filter paper under a vacuum, and then, the organic solvents were evaporated using a rotary evaporator (at 40 °C, under a vacuum). The aqueous extract was stabilized by lyophilisation (−80 °C, under a vacuum for 72 h).

### 2.3. Isolation and analysis of the essential oils

One hundred grams of air-dried aerial plant material was subjected to water distillation for five hours utilizing a British-type Clevenger apparatus. The oils were dehydrated using anhydrous sodium sulfate; thereafter, they were filtered and kept at +4 °C until testing and analysis were conducted. The oils were examined using gas chromatography equipped with a flame ionization detector (GC-FID) and gas chromatography coupled with mass spectrometry (GC/MS). The GC/MS measurements were performed on an Agilent 5975 GC-MSD system connected to an Agilent 7890A gas chromatograph (Agilent Technologies Inc., Santa Clara, CA). An HP-Innowax FSC capillary column (60 × 0.25 mm, 0.25 µm film thickness) was employed, with helium (99.99% purity) as the carrier gas at a flow rate of 1.2 mL min^−1^. Further experimental parameters are described in the supplemental materials.

### 2.4. Total phenolic and flavonoid contents

The total phenolic and flavonoid contents of the extracts were determined using established colorimetric assays, following the referenced procedure. Calibration curves were constructed using gallic acid (mg gallic acid equivalents (GAE) per g) and rutin (mg rutin equivalents (RE) per g) as standards.^28^

### 2.5. UHPLC-HRMS

The UHPLC-HRMS analyses were performed as previously described^29^ on a Q Exactive Plus mass spectrometer (Thermo Fisher Scientific, Inc.) with a heated electrospray ionization (HESI-II) probe, in negative and positive ion modes; the *m*/*z* range was from 150 to 1500. Chromatographic separation was achieved on a reversed-phase C18 column (1.8 µm, 2.1 × 100 mm), and the temperature was 40 °C. The mobile phase consisted of 0.1% formic acid in water (A) and 0.1% formic acid in acetonitrile (B), and the run time was 33 min; the flow rate and other chromatographic conditions were previously described.^26^ Xcalibur 4.2 (Thermo Scientific, Waltham, MA, USA) and MZmine 2 software were used for data processing.

### 2.6. Antioxidant assays

Antioxidant capacity was assessed using a panel of *in vitro* assays, following a previously described protocol.^28^ Reducing power was evaluated using FRAP (ferric reducing antioxidant power) and CUPRAC (cupric reducing antioxidant capacity), while radical scavenging was measured using DPPH (2,2-diphenyl-1-picrylhydrazyl) and ABTS (2,2′-azino-bis(3-ethylbenzothiazoline-6-sulfonic acid)) assays. Results from these four assays were quantified and standardized to Trolox and reported as mg Trolox equivalents (TE) per g of dried extract (mg TE per g). Total antioxidant capacity was additionally determined by the phosphomolybdenum method (PBD assay) and expressed as mmol Trolox equivalents per g (mmol TE per g). Finally, metal-chelating activity was evaluated using a chelation assay and reported as mg EDTA equivalents (EDTAE) per g of extract (mg EDTAE per g).

### 2.7. Enzyme inhibitory activity assays

The enzyme inhibitory potential of the extracts was evaluated against five key targets: acetylcholinesterase (AChE), butyrylcholinesterase (BChE), tyrosinase, α-amylase and α-glucosidase, using established colorimetric procedures.^28^ To standardise the results, inhibition was quantified using established reference compounds. Cholinesterase inhibitory activity was expressed as mg galanthamine equivalents (GALAE) per g of extract (mg GALAE per g). α-Amylase and α-glucosidase inhibitory activities were expressed as mg acarbose equivalents (ACAE) per g (mg ACAE per g), while tyrosinase inhibition was reported as mg kojic acid equivalents (KAE) per g (mg KAE per g). Lipase and elastase inhibitory effects were also evaluated as orlistat (OE) and catechin (CAE) equivalents, respectively.^28,30^ In addition, the extracts were evaluated for inhibitory activity against human carbonic anhydrase isoenzymes I and II (hCA I and hCA II).^31^

### 2.8. Cell line maintenance

The cell lines used in the presented study were obtained from the American Type Culture Collection (ATCC). The cell line panel comprised non-cancerous VERO cells, alongside selected cancer cell lines: AGS (ATCC: CRL-1739, human gastric adenocarcinoma), FaDu (ATCC: HTB-43, human hypopharyngeal squamous cell carcinoma), and RKO (ATCC: CRL-2577, human colon cancer). The VERO cells were cultured in Dulbecco's Modified Eagle Medium (DMEM, Corning, Tewksbury, MA, USA), while the remaining cell lines were cultured in Modified Eagle Medium (MEM, Corning). Additionally, culture media were enriched with antibiotics (penicillin–streptomycin solution, 100×, 30-002-CI, Corning) and fetal bovine serum (FBS, Corning). In experiments, 2% FBS was added, while 10% FBS was used for cell passaging. Phosphate-buffered saline (PBS) and trypsin were obtained from Corning; MTT (3-(4,5-dimethylthiazol-2-yl)-2,5-diphenyltetrazolium bromide) was procured from Sigma-Aldrich (St. Louis, MO, USA). The incubation was conducted in a 5% CO_2_ atmosphere at 37 °C (CO_2_ incubator, Panasonic Healthcare Co., Tokyo, Japan). The extracts were dissolved in cell culture-grade DMSO (PanReac AppliChem) to form stock solutions (50 mg mL^−1^), which were then stored at −23 °C until required.

### 2.9. Cytotoxicity testing and antiviral screening

Cytotoxicity was evaluated utilizing an MTT (3-(4,5-dimethylthiazol-2-yl)-2,5-diphenyltetrazolium bromide) assay, following the established methodology.^32^ In summary, the monolayer of selected cells in 96-well plates was treated with the extracts diluted in cell media for a period of 72 hours. At the same time, the cytotoxicity of DMSO at a concentration equal to that present in serial dilutions of tested extracts was assessed. Subsequently, cell viability was assessed through the MTT method. Absorbance measurements were recorded at wavelengths of 540 and 620 nm using a Synergy H1 Multi-Mode Microplate Reader (BioTek Instruments, Inc., Winooski, Vermont, USA), which was operated under Gen5 software (version 3.09.07; BioTek Instruments, Inc). The results were subsequently analyzed using GraphPad Prism software. The CC_50_ values, defined as the concentration at which cell viability was reduced by 50%, were obtained from dose–response curves *via* non-linear regression analysis. Additionally, the selectivity of the extracts concerning cancer cells was determined using selectivity indexes (SI = VERO CC_50_/cancer cell line CC_50_). A statistical analysis of CC_50_ variances across different cell lines was conducted utilizing GraphPad Prism, applying a two-way ANOVA with Tukey's multiple comparison test.

### 2.10. Evaluation of antiviral activity

Antiviral activity was assessed against Human Herpesvirus type 1 (HHV-1; ATCC, No. VR-260) and Human Coxsackievirus B3 (CVB3; ATCC, VR-30) propagated in the VERO cell line. The antiviral assays evaluated the influence of the tested extract on the formation of cytopathic effects (CPE) induced by HSV-1 or CVB3 using an established methodology.^33^ The VERO cells were placed in 48-well plates and incubated overnight to form a monolayer. They were then exposed to HHV-1 or CVB3 at an infectious dose of 100-fold CCID_50_ per mL (50% cell culture infectious dose) for 1 hour, while uninfected wells served as controls. Afterwards, the cells were rinsed with PBS and treated with non-toxic concentrations of the extracts, ensuring that the highest dose did not decrease VERO cell viability by more than 10%. The non-infected VERO cells (cell control) and untreated infected cells (virus control) wells were supplied with media containing 2% FBS. The plates were incubated further until a characteristic cytopathic effect (CPE) appeared in the virus control group. Routine CPE observations were made using an inverted microscope (CKX41, Olympus Corporation, Tokyo, Japan) equipped with a camera (Moticam 3+, Motic, Hong Kong), and the effects of the extracts on CPE formation were recorded with Motic Images Plus 2.0 (Motic). DMSO-treated virus-infected VERO cells were used as control cells to exclude any direct antiviral effect of the solvent. Subsequently, the plates were frozen three times at −72 °C and then thawed. The samples were collected and stored frozen at −72 °C until virus titration.

The endpoint dilution assay was used to assess the infectious titer of HHV-1 or CVB3 in the samples collected in the previous experiment. Briefly, tenfold dilutions of samples were incubated with VERO cells in 96-well plates for 72 h. Subsequently, the values of CCID_50_ were calculated using the MTT test, and the difference (Δlog) between the extract-treated infected cells (ET) and the virus control (VC) was calculated (Δlog = log CCID_50_VC − log CCID_50_ET).

DNA isolation from samples obtained during anti-HHV-1 assays was conducted utilizing a commercially available kit (QIAamp DNA Mini Kit, QIAGEN, Hilden, Germany) in accordance with the manufacturer's instructions. qPCR amplification was executed employing the SsoAdvanced Universal SYBR Green Supermix (Bio-Rad Laboratories, Life Science Group, Hercules, CA, USA) and primers (UL54F – 5′ CGCCAAGAAAATTTCATCGAG 3′, UL54R – 5′ ACATCTTGCACCACGCCAG 3′) on the CFX96 thermal cycler (Bio-Rad Laboratories). The parameters for the amplification cycle were delineated as follows: initial activation (98 °C, 3 minutes); cycling (40 repeats: denaturation (95 °C, 10 seconds), annealing and synthesis (60 °C, 30 seconds), and fluorescence acquisition); and melting curve analysis (65–95 °C, 0.5 °C increment every 5 seconds). The viral load of HHV-1 in the examined samples was evaluated in relation to the virus control using the relative quantity (ΔCq) method with CFX Manager Dx Software (Bio-Rad Laboratories).

### 2.11. Network pharmacology

The Canonical SMILES representations of the differentially abundant metabolites were collected from the PubChem repository (https://pubchem.ncbi.nlm.nih.gov). These chemical identifiers were then analyzed using the Similarity Ensemble Approach platform (SEA; https://sea.bkslab.org/) to estimate possible protein targets based on structural similarity principles.^34^ To define disease-related genes, searches were performed in the GeneCards database (https://www.genecards.org/) separately for gastric cancer, head and neck squamous cell carcinoma, and colorectal cancer. The “All sections” query mode was applied, and entries categorized under “Disorders” and “Proteins” were considered. Only protein-coding genes were retained. In order to restrict the dataset to genes strongly associated with each malignancy, a relevance score cutoff of 30 was used. Gene lists generated for each cancer type were exported and processed for downstream comparison. Redundant entries were manually removed before identifying intersecting targets between SEA-predicted proteins and cancer-related genes using Venny v2.1.0. These shared genes were regarded as potential therapeutic targets. Interaction relationships among the overlapping genes were explored through the STRING database v12.0 (https://string-db.org/). An interaction score threshold of 0.4 was selected to balance network coverage and confidence. The interaction network was subsequently examined and visualized in Cytoscape v3.10.3 for further topological assessment.

### 2.12. GO enrichment and KEGG pathway analysis

Functional enrichment analyses were performed to better understand the biological roles of the identified targets. Gene Ontology (GO) terms and Kyoto Encyclopedia of Genes and Genomes (KEGG) pathways were analyzed using the DAVID 6.8 platform (https://david.ncifcrf.gov/home.jsp). Terms with a *P* value < 0.05 were considered statistically significant. For presentation, the ten most enriched GO categories in biological process (BP), cellular component (CC), and molecular function (MF), along with the twenty most significant KEGG pathways, were selected according to their *P* values. The enrichment results were visualized using the SRplot online tool (https://www.bioinformatics.com.cn/srplot).

### 2.13. Molecular docking

Molecular docking analysis was performed to evaluate the binding potential of *Salvia limbata*-derived phytochemicals against selected protein targets. Cancer-related targets were prioritized based on network pharmacology analysis, particularly according to their topological significance within the protein–protein interaction network and enrichment results. In contrast, enzyme targets (AChE, BChE, amylase, glucosidase, and carbonic anhydrase I and II) and herpes virus-related proteins were selected based on the *in vitro* experimental findings. The representative high-resolution crystal structures of all selected targets were retrieved from the Protein Data Bank (PDB) for docking studies. Ligand structures were generated in ChemDraw and subsequently geometry-optimized using Avogadro v1.2.0. Prior to docking, polar hydrogens were added, and Gasteiger charges were assigned using AutoDockTools v4.2.6. Docking simulations were conducted using AutoDock Vina v1.1.2, with the exhaustiveness parameter set to 32 to improve conformational sampling.^35–37^ Binding sites were defined based on cavity predictions obtained from POCASA v1.1. The docking grid box was centered on the predicted active site, and detailed grid parameters for each protein are provided in Table S1.^38,39^ The root mean square deviation (RMSD) between the experimental and predicted poses was calculated to assess docking accuracy. RMSD values below 2.0 Å were considered indicative of reliable pose reproduction.^40^ Protein–ligand interaction patterns, including hydrogen bonds, hydrophobic contacts, and π–π stacking interactions, were analyzed using the Protein–Ligand Interaction Profiler (PLIP).^41,42^

### 2.14. Molecular dynamics simulation

The dynamic stability and binding characteristics of the highest-scoring protein–ligand complexes were further evaluated by molecular dynamics (MD) simulations. System construction was performed using the CHARMM-GUI platform (https://charmm-gui.org).^43^ Proteins and ligands were parameterized with the CHARMM36m force field, and each complex was placed in an explicit TIP3P water environment. To ensure electrostatic neutrality and approximate physiological conditions, counterions were added, and the ionic concentration was adjusted to 0.15 M NaCl.^44–46^ All simulations were conducted with GROMACS v2024.3.^47,48^ Short-range non-bonded interactions were treated using the Verlet cutoff scheme, while long-range electrostatic interactions were calculated using the particle-mesh Ewald (PME) method. Bonds involving hydrogen atoms were constrained with the LINCS algorithm. Energy minimization was first carried out using the steepest descent approach until the maximum force dropped below 1000 kJ mol^−1^ nm^−1^.^49,50^ The systems were subsequently equilibrated in two stages: an initial NVT phase to stabilize temperature at 310 K, followed by an NPT phase to stabilize pressure and density. Finally, 100 ns production simulations were performed under periodic boundary conditions. The stability and dynamic behavior of the protein–ligand complexes were evaluated by analyzing RMSD, root mean square fluctuation (RMSF), solvent-accessible surface area (SASA), minimum ligand–protein distance, and hydrogen bond occupancy over a 100 ns simulation trajectory.

### 2.15. Statistical analysis

All experiments were conducted in triplicate, and differences among extracts were evaluated for statistical significance. One-way ANOVA was performed in GraphPad Prism (version 9.2), followed by Tukey's post hoc test for pairwise comparisons. Statistical significance was set at *p* < 0.05. To evaluate the statistical significance of cytotoxicity results, CC_50_ values obtained for extracts from a particular plant part across different cell lines were compared. A Pearson correlation between total phenolic/flavonoid contents and biological activities was also performed using GraphPad Prism.

## 3. Results and discussion

### 3.1. Total phenolic and flavonoid content

Polyphenols are plant-derived compounds that occur naturally and affect taste, color, odor, and resistance to oxidation.^51^ Flavonoids are a class of polyphenols that are ubiquitous, responsible for plant pigmentation, and have many biologically active properties.^52^ The health benefits of flavonoids, including cancer prevention, have made them a focus of scientific interest. *Salvia* is a rich source of bioactive compounds, especially phenolic compounds and flavonoids. In the present study, the total phenolic content (TPC) and total flavonoid content (TFC) of *S. limbata* extracts were determined, and the results are given in Table 1. The results showed significant variability among the extracts from the aerial parts and roots of *S. limbata*. The plant part and solvent used for extraction appeared to significantly affect the phytochemical content. In the aerial parts, the water extract showed the highest levels of TPC, measuring 33.69 mg GAE per g, followed by the ethanol/water extract, 30.70 mg GAE per g. However, the *n*-hexane extracts of the aerial parts showed a relatively low TPC, measuring 14.52 mg GAE per g. The ethyl acetate (EA) extracts of the aerial parts showed the highest flavonoid content, followed by ethanol, measuring 29.60 mg RE per g and 22.73 mg RE per g, respectively. Conversely, the hexane extract comparatively showed the lowest TFC, measuring 8.97 mg RE per g. In accordance with the results of this study, reports have demonstrated that flavonoids are present at maximum levels in ethyl acetate extracts.^24^ Previous studies have also identified high concentrations of flavonoid compounds in ethyl acetate and methanol extracts of *Salvia* species,^53,54^ including *S. limbata*,^55^ demonstrating the potency of these solvents for extracting diverse flavonoid profiles. This was reiterated in our observations, since various extracts and different parts of the plants exhibited different total flavonoid contents, reflecting the influence of both plant tissue and extraction solvent on flavonoid distribution.

**Table 1:** Total phenolic and flavonoid contents in the tested extracts^a^

Parts	Extracts	Extraction yields (%)	TPC (mg GAE per g)	TFC (mg RE per g)
Aerial parts	*n*-Hexane	2.49	14.52 ± 0.21^h^	8.97 ± 0.33^c^
	Ethyl acetate	4.04	18.87 ± 0.19^g^	29.60 ± 0.21^a^
	Ethanol	6.34	26.47 ± 0.05^f^	22.73 ± 0.19^b^
	Ethanol/water	16.90	30.70 ± 0.50^e^	20.26 ± 0.31^b^
	Water	10.17	33.69 ± 0.55^d^	19.83 ± 0.17^b^
Roots	*n*-Hexane	1.93	41.50 ± 0.45^c^	nd
	Ethyl acetate	3.31	51.11 ± 2.14^b^	19.62 ± 3.50^b^
	Ethanol	5.71	60.32 ± 0.72^a^	31.43 ± 3.08^a^
	Ethanol/water	8.54	40.92 ± 1.04^c^	9.26 ± 0.20^c^
	Water	4.83	39.84 ± 0.47^c^	2.29 ± 0.16^d^

a Values are reported as mean ± SD of three parallel measurements. GAE: gallic acid equivalent; RE: rutin equivalent; and nd: not detected. Different letters indicate significant differences between the tested extracts (*p* < 0.05, one-way ANOVA by Tukey assay).

In the case of the root extracts, the ethanol and EA extracts exhibited high levels of TPC, recorded at 60.32 mg GAE per g and 51.11 mg GAE per g, respectively. In addition, these two extracts showed high contents of flavonoids, measuring 31.43 mg RE per g and 19.62 mg RE per g, respectively. Therefore, this plant part appeared to be rich in phytochemical content. In contrast, the *n*-hexane extract showed no TFC. These findings highlighted that there were differential levels of phenolics and flavonoids between *S. limbata* aerial parts and root and that extraction procedures had significant effects on phytochemical content.

### 3.2. Ultra-high-performance liquid chromatography coupled to high-resolution mass spectrometry (UHPLC-HRMS) profiling of the *Salvia limbata* extracts

Based on a metabolite identification strategy combining MS and MS/MS accurate masses, together with retention times, fragmentation patterns in MS/MS spectra, relative ion abundance, and comparison with reference standards and literature data,^77^ secondary metabolites were identified or tentatively annotated in *S. limbata* extracts using UHPLC-HRMS. The metabolite profiling was performed according to the essentials of Çiçek *et al.*^56^ in order to be of maximum scientific relevance. Four identification confidence levels were used (Tables S2 and 2).

Secondary metabolites in the *S. limbata* extracts^a^

**Table:** 

No.	Identified/tentatively annotated compound	Molecular formula	Exact mass	*t*_R_ (min)	*Δ* (ppm)	Level of confidence	Distribution
			[M–H]^−^				
**Hydroxybenzoic, hydroxycinnamic, acylquinic acids and derivatives**							
1	Salvianic acid A (danshensu)	C_9_H_10_O_5_	197.0455	1.78	−3.536	D1	3, 5, 6, 7, 8, 9, 10
2	Protocatechuic acid^a^	C_7_H_6_O_4_	153.0193	2.04	−7.397	B	2, 4, 7, 8, 9, 10
3	Caffeoyltartaric acid	C_13_H_12_O_9_	311.0409	2.37	5.127	D1	5, 6, 10
4	*p*-Hydroxyphenyllactic acid^a^	C_9_H_10_O_4_	181.0506	2.54	−4.762	B	1, 2, 3, 4, 5, 8, 9, 10
5	Hydroxybenzoic acid	C_7_H_6_O_3_	137.0244	2.84	−8.592	D1	1, 2, 3, 4, 5, 7, 8, 9, 10
6	*p*-Hydroxybenzoic acid^a^	C_7_H_6_O_3_	137.0244	2.94	−7.133	B	1, 3, 4, 5, 7, 8, 9, 10
7	*p*-Coumaric acid *O*-dihexoside	C_21_H_28_O_13_	487.1457	2.95	0.772	D1	5, 10
8	*p*-Coumaroyltartaric acid	C_13_H_12_O_8_	295.0459	3.11	0.371	D1	1, 5, 8
9	Chlorogenic acid^a^	C_16_H_18_O_9_	353.0878	3.20	0.070	B	5, 10
10	*p*-Coumaric acid *O*-hexoside	C_15_H_18_O_8_	325.0929	3.35	−1.479	D1	2, 4, 5, 7, 10
11	4-Caffeoylquinic acid	C_16_H_18_O_9_	353.0878	3.38	0.155	D1	5, 10
12	Feruloyltartaric acid	C_14_H_14_O_9_	325.0565	3.55	0.415	D1	2, 4, 5, 10
13	Caffeic acid^a^	C_9_H_8_O_4_	179.0350	3.57	−5.094	A2	2, 3, 4, 5, 6, 7, 8, 9, 10
14	*p*-Coumaric acid *O*-hexoside isomer	C_15_H_18_O_8_	325.0929	3.82	2.735	D1	2, 4, 5, 7, 10
15	Ferulic acid *O*-hexoside	C_16_H_20_O_9_	355.1035	3.76	0.182	D1	2, 5, 10
16	*o*-Coumaric acid^a^	C_9_H_8_O_3_	163.0401	4.55	−6.792	B	2, 3, 4, 5, 10
17	Ferulic acid^a^	C_10_H_10_O_4_	193.0506	5.41	−3.585	B	5, 9, 10
18	3,5-Dicaffeoylquinic acid	C_25_H_24_O_12_	515.1195	5.88	−1.532	B	3, 5, 10
19	4,5-Dicaffeoylquinic acid^a^	C_25_H_24_O_12_	515.1195	6.23	0.137	B	3, 5, 10

**Caffeic acid oligomers**							
20	Przewalskinic acid A	C_18_H_14_O_8_	357.0616	4.43	1.343	D1	5, 10
21	Yunnaneic acid D	C_27_H_24_O_12_	539.1195	4.95	0.465	D1	5, 10
22	Rosmarinic acid *O*-hexoside	C_24_H_26_O_13_	521.1301	5.15	5.039	D1	5, 8, 9, 10
23	Rosmarinic acid *O*-hexuronide	C_24_H_24_O_14_	535.1093	5.16	0.433	D1	5
24	Yunnaneic acid F	C_29_H_26_O_14_	597.1250	5.23	0.337	D1	5, 10
25	Sagerinic acid	C_36_H_32_O_16_	719.1618	5.94	0.907	D1	3, 4, 5, 8, 9, 10
26	Salvianolic acid K	C_27_H_24_O_13_	555.1144	6.30	1.038	D1	5, 6, 7, 9, 10
27	Rosmarinic acid^a^	C_18_H_16_O_8_	359.0772	6.34	0.416	B	2, 3, 4, 5, 6, 7, 8, 9, 10
28	Methylrosmarinate	C_19_H_18_O_8_	373.0929	7.47	−0.404	D1	2, 3, 4, 5, 10

**Phenylethanoid glycosides, iridoid, and lignans**							
29	Verbascoside^a^	C_29_H_36_O_15_	623.1981	5.50	0.347	C	5
30	Martynoside	C_31_H_40_O_15_	651.2294	7.26	1.008	D1	2, 3, 4, 5, 8, 9, 10
31	Caffeoyl-hexosyl-iridoid I	C_26_H_32_O_14_	567.1719	4.46	−2.572	D1	5, 8, 9, 10
32	Caffeoyl-hexosyl-iridoid II	C_25_H_30_O_13_	537.1614	4.51	0.588	E	4, 5, 8, 10
33	Syringaresinol *O*-hexoside	C_28_H_36_O_13_	579.2083	6.07	−2.632	D1	5, 8, 9, 10

**Flavonoids**							
34	Quercetin 3-*O*-hexuronylhexoside	C_27_H_28_O_18_	639.1203	3.53	0.787	D1	5
35	Naringenin 6,8-di-*C*-hexoside	C_27_H_32_O_15_	595.1668	3.65	0.246	D1	5
36	Apigenin 6,8-*C*-hexosyl hexoside	C_27_H_30_O_15_	593.1512	4.05	1.326	D2	5
37	Luteolin *O*-dihexuronide	C_27_H_26_O_18_	637.1046	4.37	0.444	D1	4, 5, 10
38	Luteolin 7-*O*-hexuronyl-hexoside	C_27_H_28_O_17_	487.0882	4.64	0.670	D1	5
39	Eriodictyol 7-*O*-rutinoside	C_27_H_32_O_15_	595.1668	5.13	0.347	D2	2, 3, 5
40	Apigenin 7-*O*-hexuronyl-hexoside	C_27_H_28_O_16_	607.1305	5.15	0.267	D1	5
41	Isovitexin^a^	C_21_H_20_O_10_	431.0984	5.17	−0.023	B	2, 3, 10
42	Quercetin 3-*O*-galactoside (hyperoside)^a^	C_21_H_20_O_12_	463.0882	5.20	0.390	B	2, 3, 4, 5, 8
43	Quercetin 3-*O*-glucoside (isoquercitrin)^a^	C_21_H_20_O_12_	463.0882	5.31	0.455	B	2, 3, 4, 5, 8
44	Luteolin 7-*O*-hexuronide	C_21_H_18_O_12_	461.0725	5.39	0.241	D1	2, 3, 4, 5, 9, 10
45	Luteolin 7-*O*-glucoside^a^	C_21_H_20_O_11_	447.0933	5.41	0.079	B	2, 3, 4, 8
46	Apigenin 7-*O*-glucoside^a^	C_21_H_20_O_10_	431.0984	6.10	−0.371	B	2, 3, 4, 5
47	Apigenin 7-*O*-hexuronide	C_21_H_18_O_12_	445.0776	6.15	−2.661	D1	1, 3, 4, 5, 9, 10
48	Luteolin 7-*O*-malonylhexoside	C_17_H_26_O_19_	533.0996	6.20	−1.729	D1	5
49	Hispidulin *O*-hexoside	C_22_H_22_O_11_	461.1089	6.31	−0.574	D1	2, 3, 4, 5
50	Chrysoeriol *O*-hexuronide	C_22_H_20_O_12_	475.0882	6.35	−1.682	D1	2, 3, 4, 5, 10
51	Luteolin 7-*O*-acetylhexuronide	C_23_H_20_O_13_	503.0831	6.48	1.006	D1	2, 3, 4, 5
52	Methylhypolaetin	C_16_H_12_O_7_	315.0510	6.85	0.775	D1	2
53	Apigenin 7-*O*-acetylhexoside	C_23_H_22_O_11_	473.1089	6.93	0.159	D1	5
54	Apigenin 7-*O*-acetylhexuronide	C_23_H_20_O_12_	487.0882	7.26	0.310	D1	3, 4, 2
55	Luteolin 7-*O*-acetylhexuronide isomer	C_23_H_20_O_13_	503.0831	7.27	1.006	D1	2, 3, 4, 5, 10
56	Chrysoeriol *O*-malonylpentoside	C_24_H_22_O_13_	517.0988	7.48	0.225	D1	3, 4, 5
57	Luteolin^a^	C_15_H_10_O_6_	285.0405	7.62	0.241	B	1, 2, 3, 4, 5, 7, 8, 9
58	Quercetin^a^	C_15_H_10_O_7_	301.0354	7.62	0.080	B	2, 3, 8, 9
59	Methylisoscutellarein	C_16_H_12_O_6_	299.0561	7.87	0.029	D1	2, 3
60	Apigenin 7-*O*-acetylhexuronide	C_23_H_20_O_12_	487.0882	8.06	0.433	D1	5
61	Cirsiliol^a^	C_17_H_14_O_7_	329.0667	8.29	0.468	B	2
62	Narinngenin^a^	C_15_H_12_O_5_	271.0612	8.55	0.197	B	7, 8, 9
63	Pigenin^a^	C_15_H_10_O_5_	269.0455	8.65	0.161	B	2, 3, 4, 5, 7, 8, 9
64	Chrysoeriol^a^	C_16_H_12_O_6_	299.0561	8.94	0.029	B	2, 3, 4, 5, 9
65	Jaceosidin^a^	C_17_H_14_O_7_	329.0667	9.17	0.286	B	2, 3, 6, 7, 8, 9
66	Luteolin 7-methylether	C_16_H_12_O_6_	299.0561	10.28	−0.272	D1	2, 3, 4
67	Pectolinarigenin	C_17_H_14_O_6_	313.0718	10.37	0.347	C	2, 3, 4, 5, 7, 8, 9
68	Eupatilin^a^	C_18_H_16_O_7_	343.0823	10.67	0.128	B	2, 3, 4, 5, 7, 8, 9
69	Genkwanin^a^	C_16_H_12_O_5_	283.0611	11.62	0.082	B	2, 3, 4, 7, 8, 9

a Confidence level: A2: confirmed structure including confirmed stereochemistry; B: confirmed structure except for one or more stereochemical aspects; C: tentative identification matched with a standard compound, match of at least tR, MS and MS/MS with an actual authentic standard analyzed in parallel, preferably supported by other online data. D: Tentative identification based on libraries, model compounds, *etc.*; D1: relatively reliable evidence; D2: relatively poor evidence; E: tentative candidate or tentative identification of metabolite class.^52^^a^ Comparsion with reference standard; 1-aerial parts-*n*-hexane; 2-aerial parts-ethyl acetate; 3-aerial parts-ethanol; 4-aerial parts-ethanol/water; 5-aerial parts-water; 6-roots-*n*-hexane; 7-roots-ethyl acetate; 8-roots-ethanol; 9-roots-ethanol/water; and 10-roots-water.

**Table:** 

No.	Identified/tentatively annotated compound	Molecular formula	Exact mass	*t*_R_ (min)	*Δ* (ppm)	Level of confidence	Distribution
			[M + H]^−^				
**Diterpenes**							
70	Taxodione	C_20_H_26_O_3_	315.1954	14.22	−2.822	D2	1, 2, 3, 4, 5, 6, 7, 8, 9, 10
71	20-Deoxocarnosol	C_20_H_28_O_3_	317.2111	15.60	−0.950	D2	2, 3, 6, 7, 8, 9, 10
72	Sugiol	C_20_H_28_O_2_	301.2162	15.98	−1.881	D2	2, 3, 6, 7, 8, 9, 10
73	6-Hydroxysalvonolone/carnosol	C_20_H_26_O_4_	331.1904	16.74	−3.817	D2	1, 2, 3, 4, 6, 7, 8, 9, 10
74	Viridone	C_21_H_28_O_3_	329.2111	17.07	−3.523	D2	2, 3, 6, 7, 8, 9, 10
75	Miltirone	C_19_H_22_O_2_	283.1693	19.47	−3.372	D2	6, 7, 8, 9
76	Sahandinone	C_20_H_24_O_2_	297.1849	20.78	−3.079	D2	1, 3, 4, 5, 6, 7, 8, 9, 10
77	Dehydrosugiol	C_20_H_26_O_2_	299.2006	21.91	−1.326	D2	1, 2, 3, 4, 5, 6, 7, 8, 9, 10

Four hydroxybenzoic acids (2 and 4–6), eight hydroxycinnamic acids (1, 3, 8, 11, 12, 13, 16, and 17) and their glycosides (7, 10, 14, 15, and 17), together with three caffeoylquinic acids (9, 18, and 19), were identified based on the comparison with literature data and/or reference standards (Tables S2, 2 and Fig. S1–S7).^57^

Caffeic acid oligomers are specialized metabolites typical for *Salvia* species.^58^ Compound 27 was identified as rosmarinic acid and demonstrated a fragment ion at *m*/*z* 197.044 [M–H-162.03]^−^ and a base peak at *m*/*z* 161.023 [M–H-198.05], corresponding to the cleavage of ester bonds with the loss of caffeoyl and danshensu residues, respectively. The structure of the compound was evidenced by comparison with reference standards (Tables S2, 2 and Fig. S8). Compounds 22 and 23 were tentatively annotated as rosmarinic acid hexoside and hexuronide, based on the loss of 162.05 and 176.03 Da, respectively (Tables S2 and 2). Compound 28 differed from compound 27 by a CH_2_ radical and was related to methylrosmarinate. In the MS/MS spectrum, compound 20 gave indicative fragment ions at *m*/*z* 313.072 [M–H–CO_2_]^−^, 295.064 [M–H–CO_2_–H_2_O]^−^, and 269.082 [M–H–2CO_2_–H_2_O]^−^, as well as a base peak at *m*/*z* 109.028 [C_6_H_5_O_2_]^−^. Thus, compound 20 was dereplicated as przewalskinic acid A.^59^ Two yunnaneic acids D and F (21 and 24), characterized by their intricate bicyclic structures, were dereplicated in the studied extracts based on comparison with their fragmentation patterns.^60^ The fragmentation pathway of 25 was similar to that of rosmarinic acid, with additional losses of two caffeic acids, indicating its association with sagerinic acid, which possesses a cyclobutane ring.^61^ Compound 26 differed from rosmarinic acid by an additional C_9_H_8_O_5_ (dihydroxyphenyl-dihydroxypropanoyl residue) and was annotated as salvianolic acid K.^61^

Two phenylethanoid (29 and 30), two iridoid (31 and 32), and one lignan (33) glycosides were found in the studied extracts. Compound 29 with deprotonated molecular ions at *m*/*z* 623.198 gave a typical fragmentation pattern for verbascoside, with indicative ions at *m*/*z* 461.166 [M–H–caffeoyl]^−^ and 315.108 [M–H–caffeoyl–dHex]^−^, together with ions corresponding to caffeic acid (CA) at *m*/*z* 179.034 [CA–H]^−^, 161.023 [CA–H–H_2_O]^−^, and 135.044 [CA–H–CO_2_]^−^ (Table 2 and Fig. S8). The compound was unambiguously identified by comparison with a reference standard. The MS/MS spectrum of compound 30 [M–H]^−^ at *m*/*z* 651.229 revealed ions at *m*/*z* 475.185 and 329.124, corresponding to the loss of feruloyl and deoxyhexosyl residues, respectively, and a base peak due to the dehydrated ferulic acid (FA) at *m*/*z* 175.039 [FA–H–H_2_O]^−^. Thus, 30 was tentatively identified as martynoside (Tables S2 and 2).^62^ Compound 32 gave fragment ions at *m*/*z* 341.088 [M–H–C_10_H_12_O_4_]^−^ and 179.054 [M–H–C_10_H_12_O_4_–Hex]^−^, as well as a base peak at *m*/*z* 161.023 and a fragment at *m*/*z* 135.044, revealing the presence of caffeoyl and hexosyl residues. Further fragments at *m*/*z* 195.065 [C_10_H_11_O_4_]^−^, 177.055 [C_10_H_11_O_4_–H_2_O]^−^, and 149.059 [C_10_H_11_O_4_–H_2_O–CO]^−^ could be tentatively related to caffeoyl hexosyl iridoid.^63^ Compound 31 differed from 32 by 30 Da and demonstrated a similar fragmentation pathway; it was also related to caffeoyl hexosyl iridoid (Table S2). Additionally, based on comparison with literature data, 33 was identified as syringaresinol *O*-hexoside.^64^

Thirty-six flavonoids belonging to the class of flavones, flavonols, and flavanones were annotated in the *S. limbata* extracts (Tables S2 and 2). They contained apigenin, luteolin, chrysoeriol, cirsilol, nepetin, methylhypolaetin, naringenin, eriodictyol, quercetin as an aglycone moiety and their glycosides (Tables S2 and 2). A flavonoid dereplication strategy was previously discussed in detail.^57,65^

Based on the comparison with reference standards, 41–43, 45, 57, 58, 61–65, and 67–69 were unambiguously identified. The MS/MS spectra of the identified level B compounds are presented in Fig. S9–S13. An important step in the dereplication/annotation of flavonoid glycosides was the neutral loss of 162.05, 146.05, 132.04, 176.03, 204.06 Da, corresponding to hexose, deoxyhexose, pentose, hexuronic acid and acetylhexose.^65^ A series of ions in (−) ESI/MS/MS from the neutral losses of CH_2_O (−30 Da), CO (−28 Da), CO_2_ (−44 Da), H_2_O (−18 Da), H_2_O + CO (−46 Da), 2CO (−56 Da), and CO_2_ + CO (−72 Da) was used for the dereplication of the flavonoid aglycones. Additionally, the fragment ions resulting from the Retro–Diels–Alder (RDA) reaction of the flavonoid skeleton were informative.^66^

In addition, eight diterpenes were tentatively annotated in the studied extracts, in positive ion mode (Tables S2 and 2). The typical fragmentation patterns of diterpenes included ions from the neutral loss of H_2_O, CO, H_2_O + CO, and H_2_O + 2CO.^67^ Compounds were tentatively related to diterpenes with confidence level E, based on comparison with literature data.^67^

#### Essential oil composition

Table 3 presents the chemical profile of *S. limbata* essential oil. In total, 25 constituents were identified, representing 94.31% of the oil. The oil was characterized by a complex assemblage of oxygenated diterpenoids, sesquiterpenes, and long-chain hydrocarbons. The predominant compound was 8,13-epoxy-15,16-dinorlabd-12-ene (16.87%), followed by phytol (7.84%), hexahydrofarnesylacetone (7.35%), piperitenone oxide (7.26%), and docosane (6.94%). Myristicin (6.06%), tetracosane (5.13%), and heneicosane (3.82%) were also present at noteworthy levels, indicating a substantial contribution from aliphatic hydrocarbons and their oxygenated derivatives. Similar to our findings, Afandiyev *et al.*^68^ identified 8,13-epoxy-15,16-dinorlabd-12-ene (12.1%) as one of the primary constituents in the *S. limbata* essential oil collected in Azerbaijan. However, Kurkcuoglu *et al.*^65^ investigated the chemical composition of *S. limbata* collected from two locations in Turkey and found that α-pinene and sabinene were the most abundant components. Another study by Ögütçü *et al.*^54^ identified spathulenol (29.3%) as the major component of the essential oil of *S. limbata*. In the current study, the prevalence of labdane-type diterpenoids and oxygenated terpenoids pointed to a unique chemotype, distinct from the classical monoterpene-rich *Salvia* oils,^69–71^ which generally contain high proportions of 1,8-cineole, camphor, or thujone derivatives. In contrast, monoterpenes, such as 1,8-cineole (0.55%), linalool (3.66%), and terpinen-4-ol (0.74%), occurred only in minor amounts. This compositional pattern suggested that the biological activities of this oil were mainly governed by higher-molecular-weight oxygenated constituents rather than highly volatile monoterpenes. The relatively elevated contents of oxygenated diterpenoids and phenylpropanoid derivatives (*e.g.*, myristicin) may underlie the oil's pronounced biological potential,^72–74^ as these compound classes are frequently linked to antioxidant, anti-inflammatory, and enzyme-inhibitory effects. Phytol and hexahydrofarnesylacetone, in particular, are well documented^75–77^ as bioactive, lipid-soluble molecules with membrane-interacting properties that can modulate antimicrobial and cytoprotective responses. Also, the presence of oxygenated sesquiterpenes, such as spathulenol and caryophyllene oxide, underscored the oil's therapeutic significance, as these molecules were often associated with biological activity.^78,79^ Overall, the chemical profile of *S. limbata* essential oil exhibited a unique composition, marked by the presence of oxygenated diterpenoids and long-chain hydrocarbons, which distinguished it from common *Salvia* chemotypes.

**Table 3:** Chemical composition of *S. limbata* essential oils

No.	Compounds	RRI^a^	(%)
1	1,8-Cineole	1211	0.55
2	α-Copaene	1501	2.36
3	β-Bourbonene	1531	0.27
4	Linalool	1548	3.66
5	Terpinen-4-ol	1612	0.74
6	α-Terpineol	1706	1.70
7	Germacrene D	1729	0.56
8	Geranyl acetate	1763	0.81
9	3-Methylacetophenone	1791	2.02
10	α-Calacorene	1943	2.27
11	Piperitenone oxide	1985	7.26
12	Caryophyllene oxide	2017	2.95
13	Salvial-4(14)-en-1-one	2043	1.40
14	Heneicosane	2100	3.82
15	Hexahydrofarnesylacetone	2134	7.35
16	Spathulenol	2147	2.89
17	Docosane	2200	6.94
18	8,13-Epoxy-15,16-dinorlabd-12-ene	2272	16.87
19	Sclaerel oxide	2277	2.26
20	Myristicin	2291	6.06
21	(*E*)-Nuciferol	2355	3.29
22	Tetracosane	2400	5.130
23	Hexacosane	2600	2.15
24	Phytol	2618	7.84
25	*n*-Hexadecanoic acid	2912	3.16
	**Total identified (%)**		**94.31**

a Relative retention index: Kovats index.

### 3.3. Antioxidant properties

In the human body, free radicals are continuously produced by various metabolic activities and environmental factors. These free radicals disturb the redox system, which is linked with the development of various diseases, including cancer, diabetes, and aging.^80,81^ Plant phenolics were found to play an important role in the maintenance of human health, mainly by their antioxidant potential. Antioxidants play an essential role in maintaining the balance between free radicals. In the present research, antioxidant activity was found to vary significantly for both parts of the plant and EO; the results are given in Table 4.

**Table 4:** Antioxidant properties of the tested samples^a^

Parts	Extracts	DPPH (mg TE per g)	ABTS (mg TE per g)	CUPRAC (mg TE per g)	FRAP (mg TE per g)	MCA (mg EDTAE per g)	PBD (mmol TE per g)
Aerial part	*n*-Hexane	21.15 ± 0.67^f^	34.04 ± 0.09^i^	57.06 ± 1.58^i^	35.45 ± 3.75^i^	4.72 ± 0.14^f^	1.69 ± 0.11^g^
	Ethyl acetate	24.70 ± 1.65^f^	38.23 ± 0.75^i^	79.85 ± 7.03^h^	44.16 ± 4.74^i^	17.95 ± 0.66^c^	1.96 ± 0.14^ef^
	Ethanol	42.94 ± 0.87^e^	59.94 ± 0.70^h^	143.52 ± 2.85^g^	82.12 ± 0.88^g^	15.11 ± 0.21^d^	2.01 ± 0.12^e^
	Ethanol/water	51.04 ± 0.05^e^	100.11 ± 0.06^f^	184.79 ± 3.02^f^	110.43 ± 0.64^f^	20.95 ± 0.23^b^	1.30 ± 0.10^h^
	Water	49.74 ± 0.11^e^	99.82 ± 0.05^f^	180.76 ± 1.77^f^	114.61 ± 0.59^f^	23.76 ± 0.46^a^	1.09 ± 0.05^h^
Roots	*n*-Hexane	109.92 ± 5.36^d^	287.80 ± 4.32^c^	350.63 ± 1.62^c^	279.79 ± 7.11^c^	20.14 ± 1.19^b^	3.23 ± 0.09^c^
	Ethyl acetate	296.46 ± 15.82^a^	425.44 ± 1.80^a^	503.32 ± 12.33^a^	349.34 ± 11.03^b^	21.10 ± 0.94^b^	3.49 ± 0.11^b^
	Ethanol	217.81 ± 5.16^b^	358.99 ± 9.61^b^	459.23 ± 1.30^b^	378.80 ± 2.97^a^	23.81 ± 0.28^a^	3.04 ± 0.01^c^
	Ethanol/water	127.07 ± 1.90^c^	241.54 ± 3.86^d^	290.32 ± 3.87^d^	237.19 ± 0.32^d^	23.79 ± 1.48^a^	2.64 ± 0.04^d^
	Water	125.55 ± 1.55^cd^	194.03 ± 3.61^e^	241.21 ± 2.58^e^	168.54 ± 2.02^e^	23.80 ± 0.08^a^	1.74 ± 0.02^fg^
EO		20.15 ± 0.12^f^	71.91 ± 0.70^g^	74.99 ± 2.21^h^	58.32 ± 0.22^h^	12.79 ± 0.41^e^	4.87 ± 0.01^a^

a Values are reported as mean ± SD of three parallel measurements. TE: Trolox equivalent; EDTAE: EDTA equivalent; MCA: metal chelating activity; and PBD: phosphomolybdenum. EO: essential oil. Different letters indicate significant differences among the tested extracts (*p* < 0.05, one-way ANOVA by Tukey's test).

In the aerial parts, the highest antioxidant activity was consistently observed for the ethanol/water extract, as measured by the CUPRAC (184.79 mg TE per g), ABTS (100.11 mg TE per g), and DPPH (51.04 mg TE per g) assays. The second-highest antioxidant activity was observed for the water extracts, measuring 180.76 mg TE per g, 99.82 mg TE per g, and 49.74 mg TE per g, respectively. The water extract also showed the highest FRAP and metal chelating activity (MCA), measuring 114.61 mg TE per g and 23.76 mg EDTAE per g, respectively. Prior research has shown^54^ that, compared to non-polar extracts of *S. limbata*, methanol extracts exhibit stronger antioxidant activity, which is consistent with the high antioxidant activity observed in the present study. Similarly, Salehi *et al.* reported the highest antioxidant activity of the methanolic extract.^82^ Conversely, the lowest antioxidant activity was observed for the *n*-hexane extract, which also exhibited the lowest TPC and TFC levels, indicating that phenolic compounds contributed to antioxidant activity. To confirm this finding, we performed a Pearson correlation analysis, the results of which are presented in Fig. 1. There was a strong correlation (*R* > 0.8) between the total phenolic content and the results of the radical scavenging (DPPH and ABTS) and reducing power (CUPRAC and FRAP) assays. In addition, the total phenolic content was moderately correlated with phosphomolybdenum and metal chelating ability. The high antioxidant activity of these extracts, which also showed the highest levels of phenolic compounds, supported the previously identified importance of phenolic compounds as the primary contributors to antioxidant activity.^83,84^ The antioxidant activity of *Salvia* species was mainly due to phenolic acids, such as rosmarinic and salvianolic acids, as well as specific flavonoids, including apigenin, luteolin, quercetin, and salvigenin.^85^ Mohammadhosseini *et al.* reported that the methanolic extract of *S. limbata* flowers, leaves, and stems possessed potent radical scavenging properties, with the highest activity recorded by the flower extract.^86^ Recent investigations have highlighted that *S. limbata* uses its antioxidant defense mechanisms to combat heavy metal stress, which strongly indicates its antioxidant potential.^87^

**Fig. 1:**
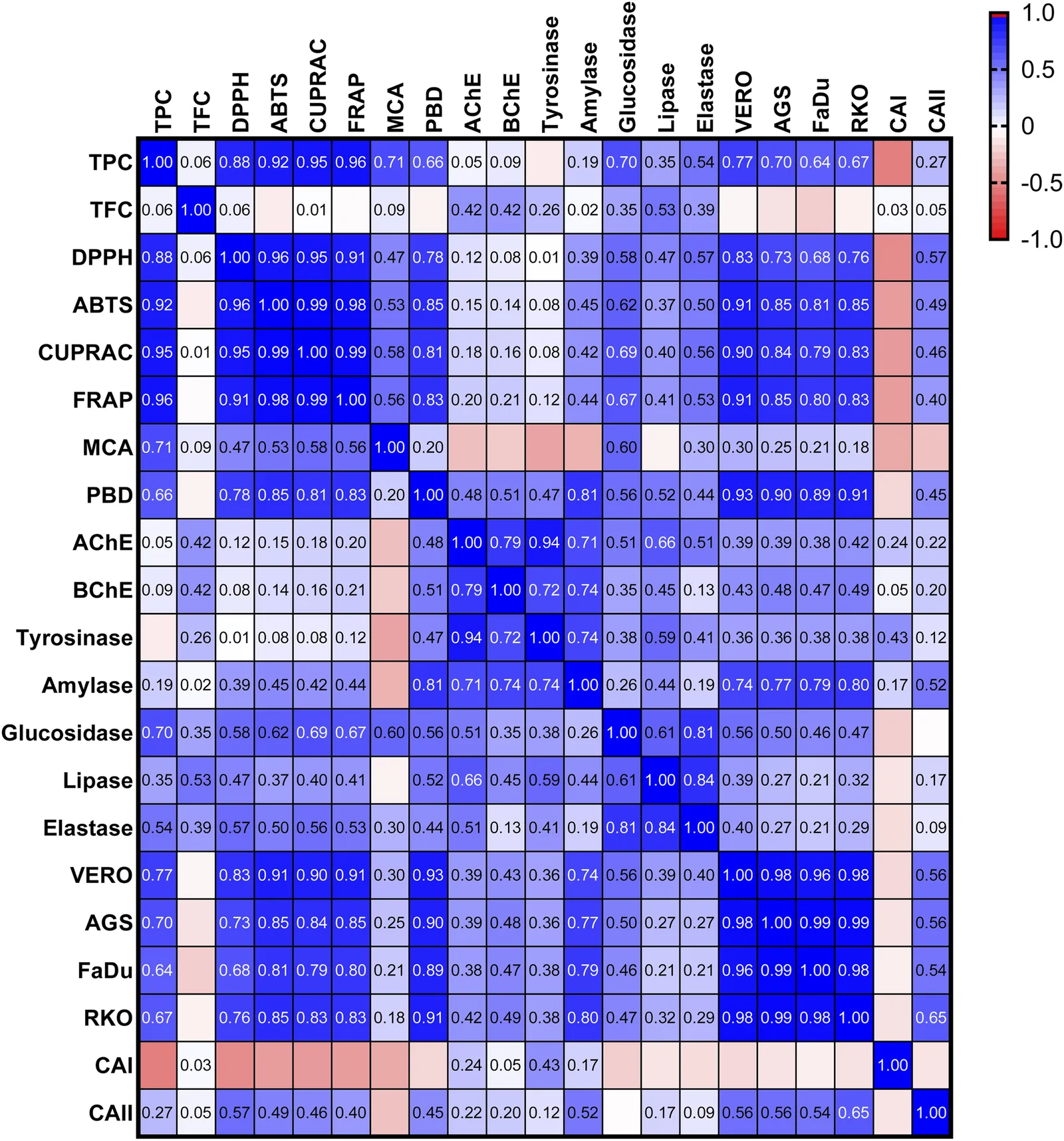
Pearson correlation between the total phenolic/flavonoid contents and biological activities (MCA: metal chelating ability and PBD: phosphomolybdenum assay).

Compared to the aerial parts, the root extracts exhibited the highest antioxidant activity, consistent with the highest phenolic and flavonoid content. The most potent antioxidant activities were shown by EA extracts, measuring 503.32 mg TE per g, 425.44 mg TE per g, and 349.34 mg TE per g in CUPRAC, ABTS, and FRAP assays, respectively. The MCA values of all root extracts exhibited nearly similar levels of activity, indicating a comparable metal-chelating potential across the different extraction solvents. A relatively low FRAP activity was shown by the water extract (168.54 mg TE per g), despite its moderately high TPC. This was consistent with a previous study, in which *S. officinalis* root extracts exhibited the strongest radical-scavenging activity.^88^

On the other hand, EO of *S. limbata* possessed potent antioxidant capacity. The EO showed the highest antioxidant activity in the CUPRAC and FRAP assays, measuring 74.99 mg TE per g and 58.32 mg TE per g, respectively. The highest PBD activity was found in the EO, with a recorded value of 4.87 mmol TE per g. In addition, there were several *in vitro*^89–91^ and *in vivo*^92,93^ studies that evaluated the protective effects of sage extracts in cultured cells and in animal models, following the induction of oxidative stress.

### 3.4. Enzyme inhibitory effects

The increasing aging population and the improvement in the quality of healthcare services worldwide have increased the need for developing new therapies against complex diseases, such as neurodegenerative disorders and cancer. Natural products are increasingly recognized as valuable sources of multifunctional bioactive compounds capable of modulating multiple biological targets simultaneously.^94,95^ Enzymes are important targets in drug discovery, as the modulation of enzyme activity directly affects the biological system, with almost half of the drugs in clinical use inhibiting enzymes.^96^

In the current study, we targeted several clinically relevant enzymes, including cholinesterase, tyrosinase, α-amylase, α-glucosidase, lipase, and elastase. The enzyme inhibitory activity of the aerial-part and root extracts of *S. limbata* and its EO indicated that there were significant variations in extract and enzyme specificity. The results are shown in Table 5.

**Table 5:** Enzyme inhibitory effects of the tested samples^a^

Parts	Extracts	AChE (mg GALAE per g)	BChE (mg GALAE per g)	Tyrosinase (mg KAE per g)	Amylase (mmol ACAE per g)	Glucosidase (mmol ACAE per g)	Lipase (mg OE per g)	Elastase (mg CAE per g)
Aerial parts	*n*-Hexane	1.65 ± 0.14^d^	1.44 ± 0.33^b^	55.94 ± 0.32^c^	0.98 ± 0.03^c^	0.17 ± 0.04^e^	29.16 ± 2.20^e^	na
	Ethyl acetate	2.23 ± 0.10^abc^	2.90 ± 0.21^a^	58.47 ± 0.70^bc^	0.99 ± 0.02^c^	0.52 ± 0.05^d^	27.10 ± 2.02^e^	na
	Ethanol	2.38 ± 0.09^ab^	2.76 ± 0.13^a^	58.27 ± 0.87^bc^	0.85 ± 0.01^d^	0.80 ± 0.02^bc^	62.65 ± 2.71^c^	1.89 ± 0.18^b^
	Ethanol/water	2.25 ± 0.04^abc^	1.15 ± 0.05^bc^	55.83 ± 0.25^cd^	0.38 ± 0.02^f^	0.79 ± 0.06^bc^	24.01 ± 2.97^e^	1.92 ± 0.13^b^
	Water	0.14 ± 0.03^e^	0.31 ± 0.07^d^	30.99 ± 0.81^e^	0.12 ± 0.02^g^	0.48 ± 0.02^d^	na	na
Roots	*n*-Hexane	2.14 ± 0.13^bc^	2.93 ± 0.10^a^	60.51 ± 1.83^b^	1.51 ± 0.05^a^	0.70 ± 0.03^c^	6.04 ± 1.29^f^	na
	Ethyl acetate	2.02 ± 0.01^c^	1.66 ± 0.13^b^	52.74 ± 1.78^d^	1.38 ± 0.08^b^	0.84 ± 0.07^ab^	59.40 ± 0.79^cd^	2.45 ± 0.11^a^
	Ethanol	2.46 ± 0.13^a^	3.14 ± 0.42^a^	57.33 ± 1.15^c^	1.03 ± 0.01^c^	0.94 ± 0.01^a^	72.56 ± 3.78^b^	2.40 ± 0.39^a^
	Ethanol/water	1.67 ± 0.09^d^	0.73 ± 0.08^cd^	57.73 ± 1.08^bc^	0.71 ± 0.01^e^	0.81 ± 0.01^bc^	53.98 ± 2.02^d^	2.42 ± 0.22^a^
	Water	0.06 ± 0.01^e^	0.24 ± 0.04^d^	25.01 ± 1.07^f^	0.13 ± 0.01^g^	0.48 ± 0.04^d^	na	na
EO		2.49 ± 0.09^a^	3.36 ± 0.27^a^	65.07 ± 0.70^a^	0.76 ± 0.03^de^	0.79 ± 0.03^bc^	109.48 ± 2.67^a^	na

a Values are reported as mean ± SD of three parallel measurements. GALAE: galantamine equivalent; KAE: kojic acid equivalent; ACAE: acarbose equivalent; OE: orlistat equivalent; and CAE: catechin equivalent. EO: essential oil. Different letters indicate significant differences among the tested extracts (*p* < 0.05, one-way ANOVA by Tukey's test).

The aerial-part ethanol extracts exhibited potent inhibitory activity against AChE, tyrosinase, and lipase, as well as notable activity against elastase, indicating their potential as a broad-spectrum enzyme inhibitor. Ethyl acetate extracts, on the other hand, showed the most potent BChE, α-amylase, and tyrosinase inhibitory activity, measuring 2.90 mg GALAE per g, 0.99 mmol ACAE per g, and 58.47 mg KAE per g, respectively. The high inhibitory activity of the ethanol and EA extracts may be linked to their high levels of TFC, as suggested by previous studies, which report that flavonoid-rich extracts are potent inhibitors of cholinesterases^97,98^ and metabolic enzymes.^99,100^ As shown in Fig. 1, there was a moderate correlation between the total flavonoid content and the observed AChE and BChE inhibitory effects (*R* > 0.4). These findings can be explained by the presence of non-flavonoid inhibitors, including alkaloids, in these extracts. Conversely, the water extract of the aerial parts exhibited the lowest inhibitory activity against all enzymes. Similarly, the root's ethanol extracts exhibited the strongest inhibitory activities against AChE (2.46 mg GALAE per g), BChE (3.14 mg GALAE per g), α-glucosidase (0.94 mmol ACAE per g), and lipase (72.56 mg OE per g), suggesting neuroprotective^101^ and antidiabetic effects.^102^ However, the lowest inhibitory activities were observed for the water extract, which was in accordance with its lowest phytochemical contents. The root extracts, except *n*-hexane and water extracts, showed potent inhibition of elastase, suggesting an anti-ageing effect.^103^ As shown in Fig. 1, the correlation analysis revealed that the total phenolic content was strongly correlated with the glucosidase and elastase inhibitory effects (*R* > 0.5).

The EO showed the strongest inhibitory activity against several enzymes, including AChE (2.49 mg GALAE per g), BChE (3.36 mg GALAE per g), tyrosinase (65.07 mg KAE per g), and lipase (109.48 mg OE per g), outperforming all solvent extracts. The strongest anti-enzymatic activity of EO suggested that non-phenolic volatile compounds could play a major role in the inhibition of enzymes. EOs possessed high amounts of phytol, myristicin, and piperitenone oxide. Published literature suggested that certain EOs with bioactive components, such as phytol and myristicin, showed promising inhibition towards AChE and BChE enzymes.^104,105^ In addition, phytol demonstrates inhibitory potential towards tyrosinase enzyme inhibition.^106^ Similarly, the EO rich in piperitenone oxide displayed its activity as an AChE inhibitor.^107^ These results emphasize that, although phenolic and flavonoid compounds contribute significantly to enzyme inhibition, other classes of bioactive compounds, such as terpenoids, in the EO could play a synergistic or additive inhibitory role.

### 3.5. Carbonic anhydrase inhibitory effects

The carbonic anhydrase (CA) inhibitory activity of plant extracts has been correlated with the bioavailability of different secondary metabolites, which include phenolics, flavonoids, alkaloids, and terpenoids. These compounds may also contribute to the diuretic properties of these plants.^108,109^

In the current study, the results of carbonic anhydrase (CA I and CA II) inhibition showed moderate activity for most of the extracts, with IC_50_ values of 29.74–38.71 µg mL^−1^ for CA I and 49.50–182.36 µg mL^−1^ for CA II. The results are given in Table 6. Among the extracts, the EA extract of the aerial parts showed the highest CA I inhibitory activity (IC_50_ = 29.74 µg mL^−1^), while its root extract showed the highest CA II inhibitory activity (IC_50_ = 49.50 µg mL^−1^). Conversely, the root ethanol/water extract had the lowest CA II inhibitory activity (IC_50_ = 182.36 µg mL^−1^). However, the EO exhibited significantly greater inhibition of both CA I (IC_50_ = 10.93 µg mL^−1^) and CA II (IC_50_ = 27.07 µg mL^−1^) than all solvent extracts, although it remained less active than the standard drug, acetazolamide. These results indicate that CA inhibition may be more complex and influenced by specific active phytochemicals rather than by total phenolic or flavonoid content, especially in the EO, suggesting that *S. limbata* has potential as a natural source of CA inhibitors for the management of CA-related disorders. This was also confirmed by the correlation analysis (Fig. 1), which showed no correlation between total phenolic content and carbonic anhydrase inhibitory effects. According to Jia *et al.*, the CA inhibitory activity of *S. miltiorrhiza* has been correlated with the bioavailability of phenolic acids and diterpenoids, which include salvianolic acid C and tanshinone IIa.^108^ Similarly, the EO of *S. hortensis* has been reported to exhibit notable CA inhibitory activity.^110^ In addition, Karakaya *et al.* (2020) demonstrated the inhibitory effects of *Angelica purpurascens* EO constituents on CA through enzyme assays.^111^ Furthermore, Costa *et al.* identified ten compounds commonly present in EOs as promising CA inhibitors based on molecular docking studies.^112^

**Table 6:** Carbonic anhydrase inhibitory effects of the tested samples

Parts	Extracts	CA I		CA II	
		IC_50_ (µg mL^−1^)	*R* ^2^	IC_50_ (µg mL^−1^)	*R* ^2^
Aerial parts	*n*-Hexane	30.26	0.9374	66.00	0.9555
	Ethyl acetate	29.74	0.9645	100.43	0.9909
	Ethanol	32.68	0.9678	123.75	0.9439
	Ethanol/water	30.52	0.9240	86.62	0.9226
	Water	30.93	0.9333	113.60	0.9368
Roots	*n*-Hexane	30.80	0.9664	79.65	0.9268
	Ethyl acetate	33.00	0.9668	49.50	0.9793
	Ethanol	36.09	0.9852	73.72	0.9422
	Ethanol/water	30.39	0.9186	182.36	0.9540
	Water	38.71	0.9294	94.93	0.9475
Essential oil		10.93	0.9157	27.07	0.9593
Standard inhibitor	Asetazolamid (ng mL^−1^)	4.23	0.9836	4.81	0.9940

### 3.6. Cytotoxicity and anticancer selectivity

The evaluation of the cytotoxicity results was conducted based on the classification of plant extract cytotoxicity proposed by the National Cancer Institute (NCI)^113^ and information from earlier studies.^114,115^ Considering the proposed classification, *S. limbata* leaf hexane (SLA_Hex), ethyl acetate (SLA_EtOAc) and ethanolic extracts (SlEtOH) were moderately cytotoxic (CC_50_ in the range of 21–200 µg mL^−1^) to non-cancerous and all cancer-derived cell lines (Table 7). The extract obtained from the leaves with 70% ethanol (SLA_AqEtOH) was not toxic (CC_50_ > 500 µg mL^−1^) to VERO cells, weakly cytotoxic (CC_50_ in the range of 201–500 µg mL^−1^) to AGS and RKO, and moderately cytotoxic to FaDu. Finally, the *S. limbata* leaf aqueous extract (SLA_Aq) was found to have a weak effect on the viability of FaDu cells while being non-cytotoxic to the other tested cell lines. The evaluation of anticancer selectivity revealed that SLA_Hex and SLA_EtOAc exhibited selective toxicity towards FaDu and RKO, with a selectivity index (SI) ranging from 2.62 to 3.48. This was also supported by dose–response curves, as presented in Fig. 2A and C. SLA_EtOH and SLA_AqEtOH exerted selective anticancer effects not only on FaDu and RKO but also towards AGS cells, with SI between 2.55 and 5.1. Interestingly, Fig. 2F shows that in the case of FaDu cells, the selectivity of *S. limbata* leaf extracts increases with the polarity of the solvent used for the extraction, starting with 2.62 for hexane, through 3.48, 4.33, up to 5.10 for ethyl acetate, ethanol, and 70% ethanol, respectively. However, when water was used as an extraction solvent, the selectivity decreased.

**Table 7:** Cytotoxicity of the *S. limbata* extracts on non-cancerous and cancer-derived cells^a^

*Salvia limbata*		VERO	AGS		FaDu		RKO	
		CC_50_	CC_50_	SI	CC_50_	SI	CC_50_	SI
Aerial	Hexane	108.45 ± 6.85^e^	104.08 ± 3.50^e^	1.04	41.46 ± 1.86^g^	2.62	37.52 ± 1.57^g^	2.89
	Ethyl acetate	135.38 ± 5.46^d^	79.22 ± 4.28^f^	1.71	38.91 ± 3.18^g^	3.48	45.73 ± 1.56^g^	2.96
	Ethanol	175.73 ± 9.85^c^	57.55 ± 5.07^f,g^	3.05	40.61 ± 3.03^g^	4.33	56.07 ± 2.72^f,g^	3.13
	70% ethanol	711.20 ± 26.86^a^	279.33 ± 4.68^b^	2.55	139.45 ± 17.12^d^	5.10	263.35 ± 12.81^b^	2.70
	Water	>1000	>1000	na	274.35 ± 15.35	>3.64	>1000	na
Root	Hexane	3.33 ± 0.23^e,g^	2.53 ± 0.26^f,g^	1.31	2.01 ± 0.07^g^	1.66	4.41 ± 0.38^d,e^	0.75
	Ethyl acetate	3.03 ± 0.24^e,g^	2.97 ± 0.4^e,g^	1.02	2.56 ± 0.20^f,g^	1.19	4.20 ± 0.18^e^	0.72
	Ethanol	3.65 ± 0.29^e,f^	3.72 ± 0.5^e,f^	0.98	3.77 ± 0.24^e,f^	0.97	6.00 ± 0.39^d^	0.61
	70% ethanol	8.15 ± 0.55^c^	11.40 ± 1.81^b^	0.71	9.20 ± 0.64^c^	0.89	23.28 ± 1.22^a^	0.35
	Water	557.05 ± 42.1^a^	495.73 ± 53.49^a^	1.12	190.88 ± 20.64^b^	2.92	>1000	<0.56
Essential oil		83.24 ± 2.47^c^	35.86 ± 2.07^d^	2.32	22.24 ± 1.48^e^	3.74	41.21 ± 2.52^d^	2.02

a CC_50_ (µg mL^−1^, mean ± SD, *n* = 4). Different letters indicate significant differences among the tested extracts (*p* < 0.05, one-way ANOVA by Tukey's test).

**Fig. 2:**
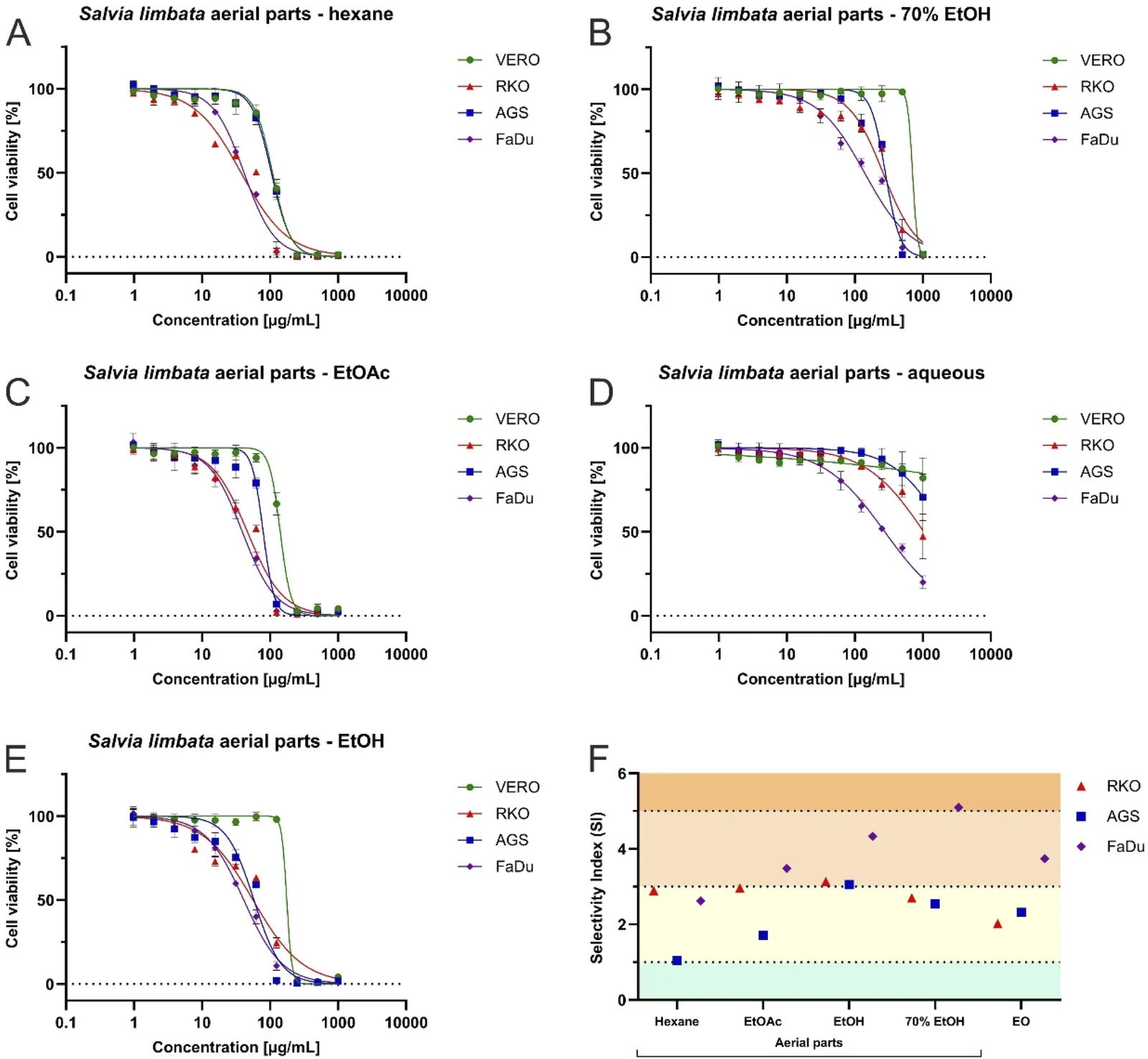
Dose–response effect of *S. limbata* aerial part extracts on cell lines. (A) *n*-Hexane extract; (B) 70% EtOH extract; (C) ethyl acetate extract; (D) aqueous extract; (E) EtOH extract; (F) selectivity index.

The *S. limbata* root aqueous extract (SLR_Aq) was not cytotoxic to most of the cell lines included in our study, except FaDu cells (Fig. 3D). Interestingly, the remaining extracts obtained from the roots were highly cytotoxic (CC_50_ < 20 µg mL^−1^) to all cell lines, but no anticancer selectivity was observed for these extracts. Moreover, RKO cells were found to be the most resistant cells to all extracts obtained from the roots of *S. limbata* (Fig. 3A–E). As shown by the correlation analysis (Fig. 1), there was a moderate positive correlation with the TPC content, while there was no correlation with TFC.

**Fig. 3:**
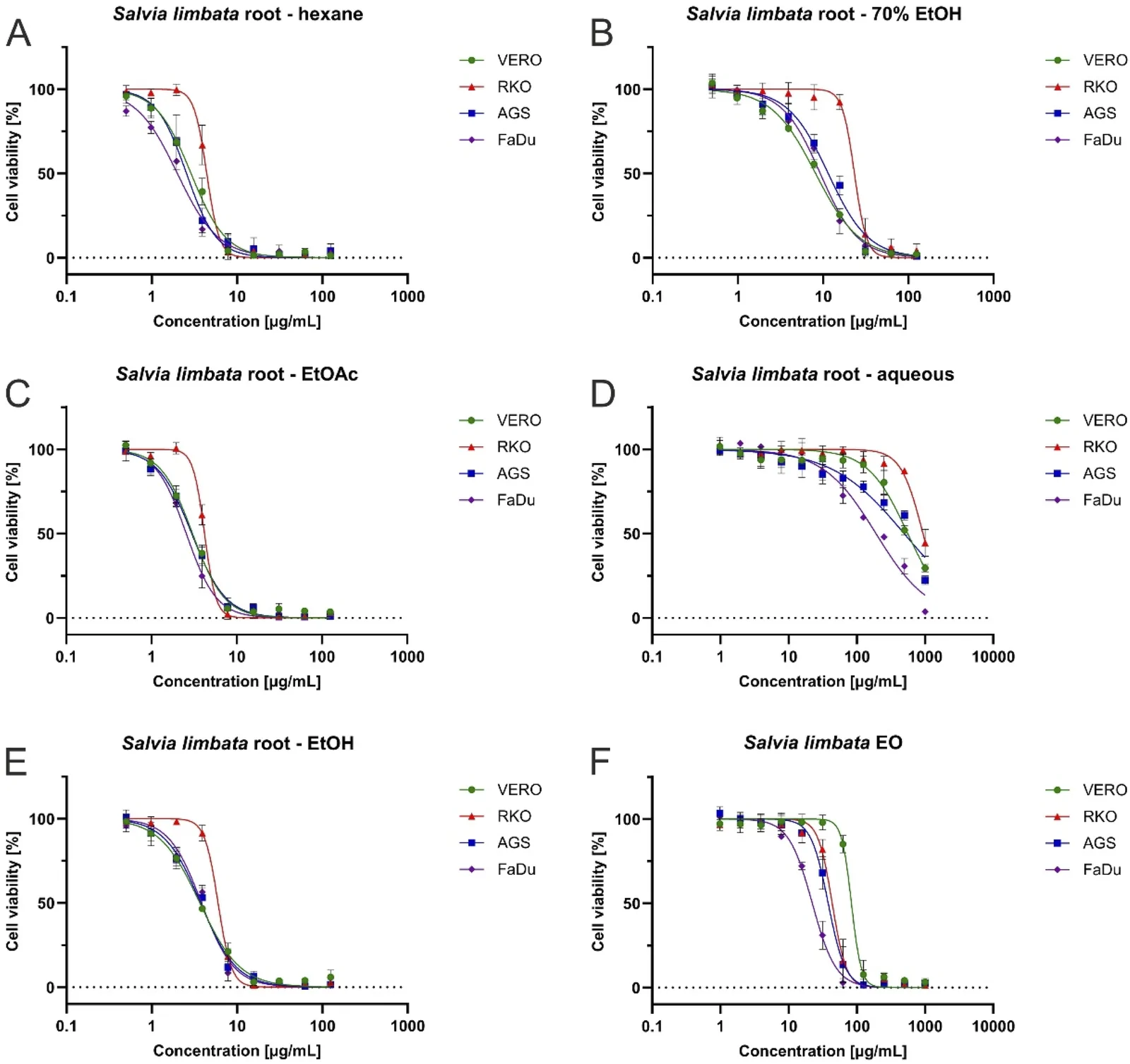
Dose–response effect of *S. limbata* root extract on cell lines. (A) *n*-Hexane extract; (B) 70% EtOH extract; (C) ethyl acetate extract; (D) aqueous extract; (E) EtOH extract; (F) essential oil.

The essential oil obtained from *S. limbata* (SLEO) exhibited moderate cytotoxicity against the tested cells (Fig. 2F), with the highest selectivity observed for FaDu (3.74). In the case of AGS and RKO cells, the SI was 2.32 and 2.02, respectively. When DMSO was used as a solvent for the extracts and essential oil, it did not exert any cytotoxicity at the concentrations present in the dilutions of the tested samples.

### 3.7. Antiviral activity

The incubation of SLA_EtOH (100 µg mL^−1^; Fig. 3E), SLA_AqEtOH (250 µg mL^−1^; Fig. 4G), and SLA_EO (40 µg mL^−1^; Fig. 3F) with HHV-1-infected VERO cells showed that virus-induced CPE was noticeably reduced compared with that of the virus control (Fig. 4A). However, none of these extracts was able to fully suppress CPE formation. Subsequent virus titration assay (Fig. 5A and B) showed that treatment with SLA_EtOH, SLA_AqEtOH, and SLA_EO reduced the infectious titer of HHV-1. In fact, in the cells treated with SLA_AqEtOH (250 µg mL^−1^), the infectious titer could not be evaluated, as no signs of HHV-1-induced cytopathic effects (CPE) were observed, even at the lowest tested dilution. Considering the infectious titer of the virus control, SLA_AqEtOH reduced the HHV-1 infectious titer by at least 5 log (Table 8), indicating a significant antiviral potential against HHV-1. A similar effect was observed for acyclovir (Fig. 4A). At low concentrations of SLA_AqEtOH, 200 and 125 µg mL^−1^, the titration assay also did not reveal signs of CPE at 10-fold dilution (Fig. 5B). However, when the titration of SLA_AqEtOH at 100 µg mL^−1^ was performed at a 10-fold dilution, there were some noticeable signs of HHV-1-induced CPE. This resulted in approximately a 35% reduction in cellular viability, and the calculated HHV-1 infectious titer reduction was 3.93 log compared with that of the virus control. SLA_EtOH and SLA_EO also dose-dependently reduced the HHV-1 infectious titer. SLA_EtOH, at the concentrations of 100 µg mL^−1^ and 80 µg mL^−1^, reduced the infectious titer by 2.39 and 1.31 log, while SLA_EO (40 µg mL^−1^) reduced the infectious titer by 1.22 log, as compared to the virus control. The rest of the *S. limbata* extracts did not influence the virus-induced CPE, and subsequent end-point titration revealed a similar HHV-1 infectious titer in the extract-treated infected cells and the virus control. The HHV-1-infected VERO cells treated with DMSO at a dose equal to the one present in the highest concentration of tested samples showed a CPE resembling that of the HHV-1 virus control, indicating the lack of any noticeable antiviral effect.

**Fig. 4:**
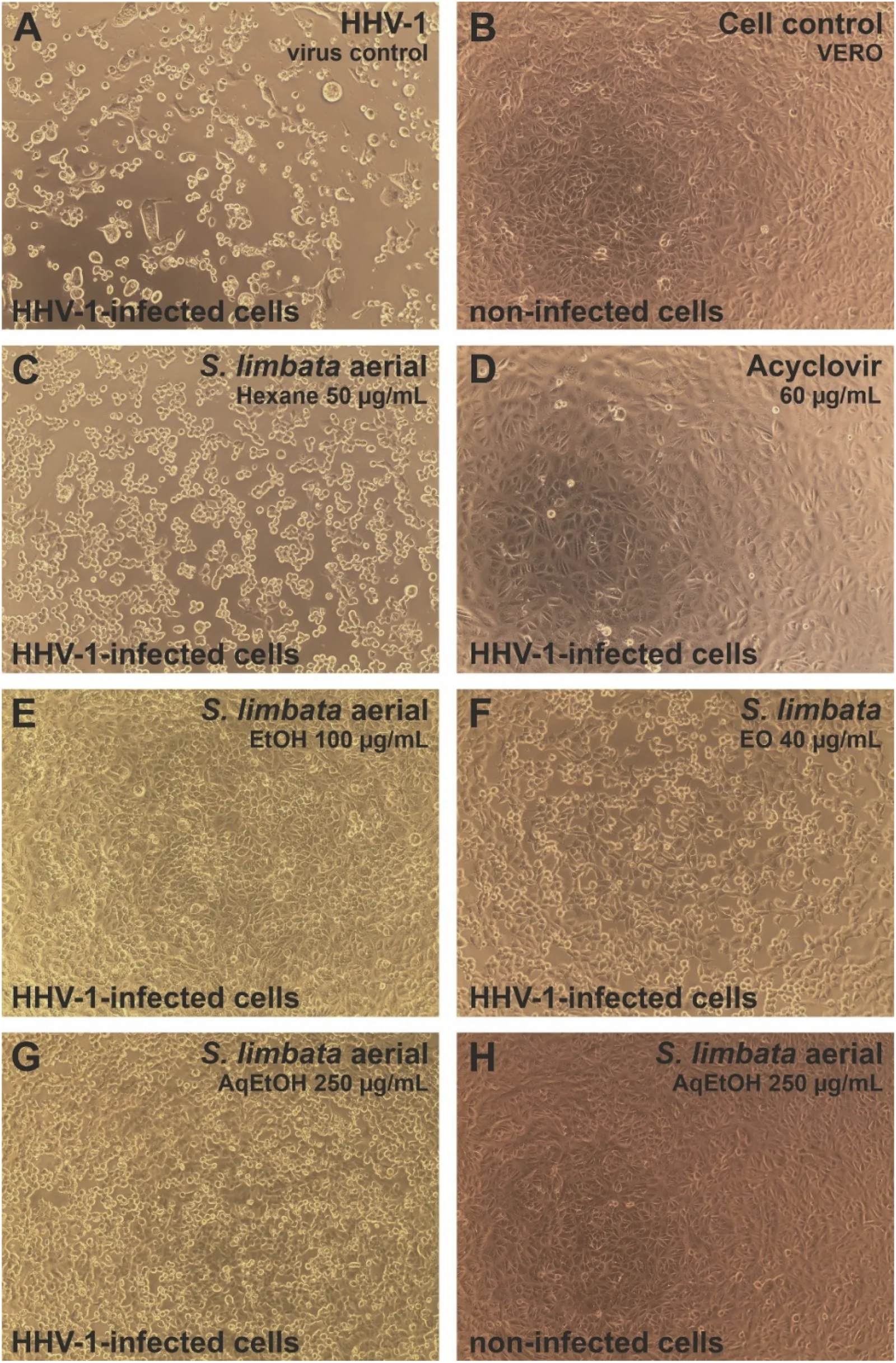
Influence of *S. limbata* extracts on the HHV-1 induced cytopathic effect in virus-infected VERO cells. (A) HHV-1 virus control; (B) cell control; (C) *n*-hexane extract of aerial parts; (D) acyclovir; (E) the EtOH extract of aerial part; (F) the essential oil; (G) 70% ethanol extract of aerial part with HHV-1-infected cells; (H) 70% ethanol extract of aerial part with non-infected cells.

**Fig. 5:**
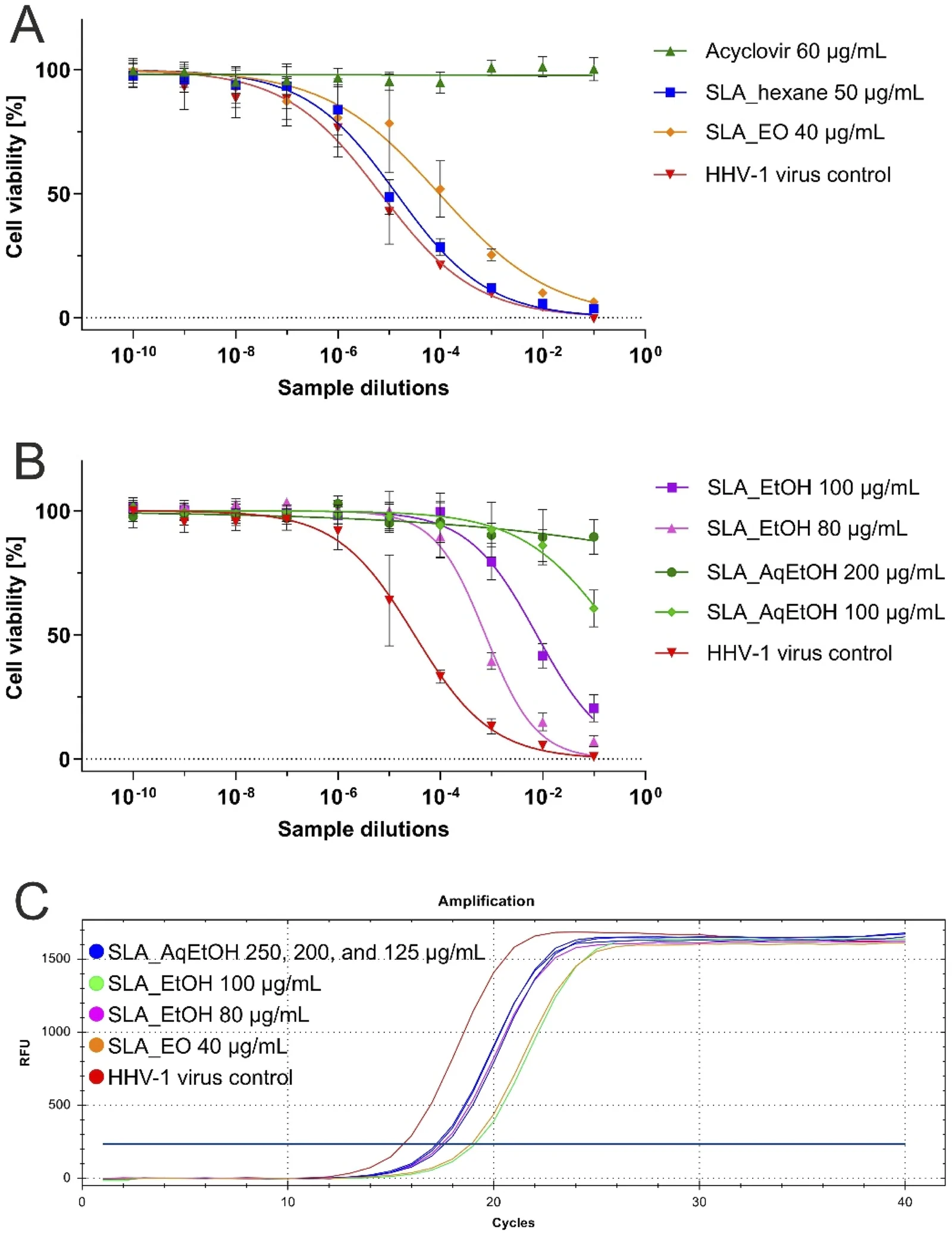
Evaluation of HHV-1 viral infectious titer and viral DNA reduction ((A) and (B) end-point virus titration for the reduction of HHV-1 infectious titer; (C) qPCR amplification for the reduction of HHV-1 DNA).

**Table 8:** Reduction of the HHV-1 infectious titer

*Salvia limbata*	Concentration (µg mL^−1^)	HHV-1 infectious titer	
		Δlog^a^	
Aerial	Hexane	50	0.29
	Ethyl acetate	60	0.08
	Ethanol	100	2.39 ± 0.1
		80	1.31 ± 0.15
	70% ethanol	250, 200, 125	>5
		100	3.93 ± 0.16
	Water	500	0.07
Root	Hexane		−0.02
	Ethyl acetate	1	0.04
	Ethanol		0.02
	70% ethanol	2	0.07
	Water	120	0.24
Essential oil		40	1.22 ± 0.11
		30	0.94 ± 0.08
Acyclovir		60	>5

a Δlog = log CCID_50_VC − log CCID_50_ET.

To evaluate whether the observed anti-herpesviral effects of selected *S. limbata* extracts resulted from the inhibition of the nucleic acid synthesis of HHV-1 during replication in the virus-infected VERO cells, the relative amount of HHV-1 DNA (viral load) was measured using qPCR and primers for the UL54 coding region, encoding ICP27—a regulatory protein required for HHV-1 infection (Fig. 5C). The highest reduction of HHV-1 DNA, by 1.1 log, was observed for SLA_EtOH at a concentration of 100 µg mL^−1^, while at a lower concentration of 80 µg mL^−1^, the viral load was reduced by 0.56 log. Additionally, SLA_EO (40 µg mL^−1^) was able to reduce the viral load by 1 log. Interestingly, SLA_AqEtOH, at all tested concentrations, was able to reduce the HHV-1 DNA by only 0.48–0.6 log. The infectious viral titer showed significantly greater inhibition, but it is important to note that the direct comparisons of these values can be challenging. The end-point dilution assay measured the infectious titer, focusing only on viral particles that could infect permissive cells. In contrast, qPCR quantified specific target DNA sequences for HHV-1 within the collected samples. This target DNA could exist in both infectious and non-infectious viral particles and could also be released from virus-infected cells. Taken together, the noticeable, yet low impact of *S. limbata* extracts on the reduction of HHV-1 viral load may suggest that the abovementioned significant reduction of HHV-1 infectious titer may be the result of the inhibition of late stages of HHV-1 replication, possibly during the production of structural proteins or the assembly and maturation of viral progeny.

The promising inhibition of HHV-1 replication prompted us to continue evaluating the antiviral potential of *S. limbata* extracts. Thus, the antiviral experiments were conducted against the CVB3-infected VERO cells. The typical CPE induced by CVB3 in VERO cells (CVB3 virus control) is shown in Fig. 6A. The treatment with SLA_EtOH at a concentration of 100 µg mL^−1^ noticeably decreased the intensity of CPE (Fig. 6E). Interestingly, even more profound inhibition of CPE was observed for SLA_EtOAc at a concentration of 60 µg mL^−1^ (Fig. 6C), where a monolayer of VERO cells was present, resembling the non-infected VERO cell control (Fig. 6B). Notably, the monolayer of VERO cells, as shown in Fig. 5C, had a lower density than the cell control, and there were evident signs of CVB3-induced CPE, such as cell rounding or cell lysis, resulting in viral plaque formation. This effect was dose-dependent, and at 50 µg mL^−1^ of SLA_EtOAc, the signs of CVB3-induced CPE were more profound (data not shown). In contrast, the broad-spectrum antiviral drug, ribavirin (RBV), decreased CPE, as shown in Fig. 6D, but the density of the VERO monolayer was much lower than in the non-infected cell control. There are currently no approved antiviral agents against CVB3, so using a more effective reference antiviral drug was not possible. Importantly, in the case of using SLA_AqEtOH (Fig. 6F), there was no effect on the CPE in CVB3-infected VERO cells. DMSO-supplemented media did not inhibit CVB3-induced CPE. Subsequent CVB3 end-point titration (Fig. 6G) revealed that SLA_EtOAc at concentrations of 60 and 50 µg mL^−1^ reduced the CVB3 infectious titer by 0.98 and 0.36 log, respectively, compared to the CVB3 virus control. Thus, despite the evident inhibition of virus-induced CPE, SLA_EtOAc did not exert significant antiviral activity against CVB3 replication in VERO cells. Other tested extracts also did not show a substantial reduction in the infectious titer of CVB3. Ribavirin managed to reduce the CVB3 infectious titer by 1.66 log, compared to the CVB3 virus control.

**Fig. 6:**
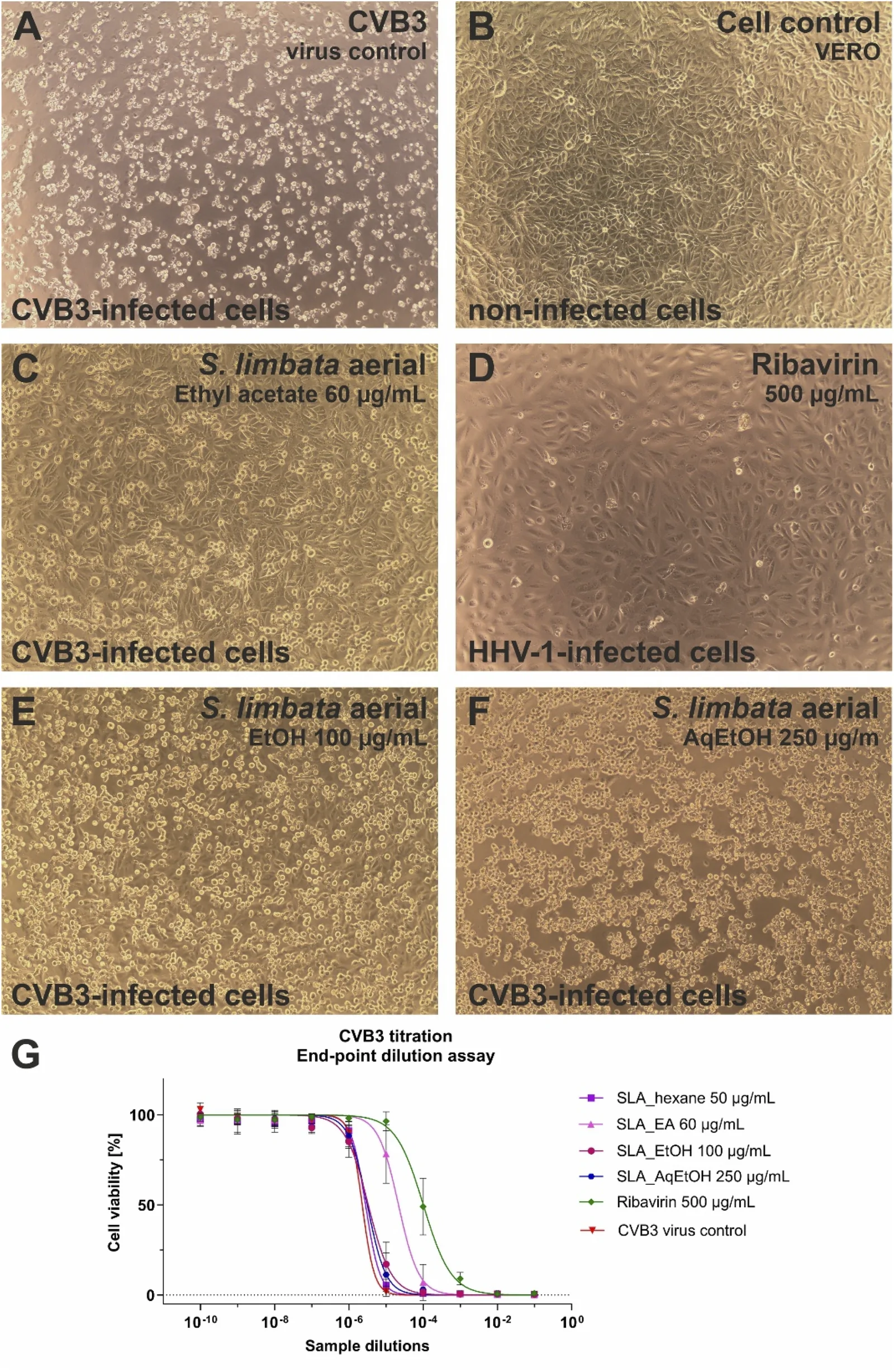
Evaluation of the antiviral potential of *S. limbata* extracts against CVB3-infected VERO cells. (A) CVB3 virus control; (B) cell control; (C) the ethyl acetate extract of aerial part; (D) ribavirin; (E) the EtOH extract of aerial part with 100 µg mL^−1^; (F) the EtOH extract of aerial part with 250 µg mL^−1^; (G) end-point dilution assay.

### 3.8. Network pharmacological analysis of metabolites in *Salvia limbata*

Twenty-three major metabolites identified in the extracts were subjected to network pharmacology analysis using SMILES data retrieved from the PubChem database. A total of 470 putative targets were initially predicted, and after the removal of duplicates, 214 unique targets were retained for further evaluation (Fig. 7A). These targets were used to construct a protein–protein interaction (PPI) network in Cytoscape. Topological analysis using the Maximal Clique Centrality (MCC) algorithm identified the top-ranked hub genes within the global network (Fig. 7E). The integration of MCC results enabled the identification of nine core targets within the overall network structure. Disease enrichment analysis demonstrated associations with several cancer-related pathways. For gastric cancer and colorectal cancer, only two overlapping targets (HRAS and EGFR) were identified (Fig. 7B). Due to the limited number of nodes in these disease-specific subnetworks, further hub prioritization or clustering analysis was not conducted, as meaningful centrality scoring and module detection required sufficient network complexity. Accordingly, HRAS and EGFR were retained directly as candidate targets. In contrast, head and neck squamous cell carcinoma involved a broad set of targets, including ERCC4, HRAS, ESR1, NFE2L2, NSD2, PTPN11, EP300, EGFR, and PPARG (Fig. 7C). Overall, the findings indicate a multi-target interaction profile for *Salvia limbata* metabolites and provide a foundation for subsequent molecular validation studies.

**Fig. 7:**
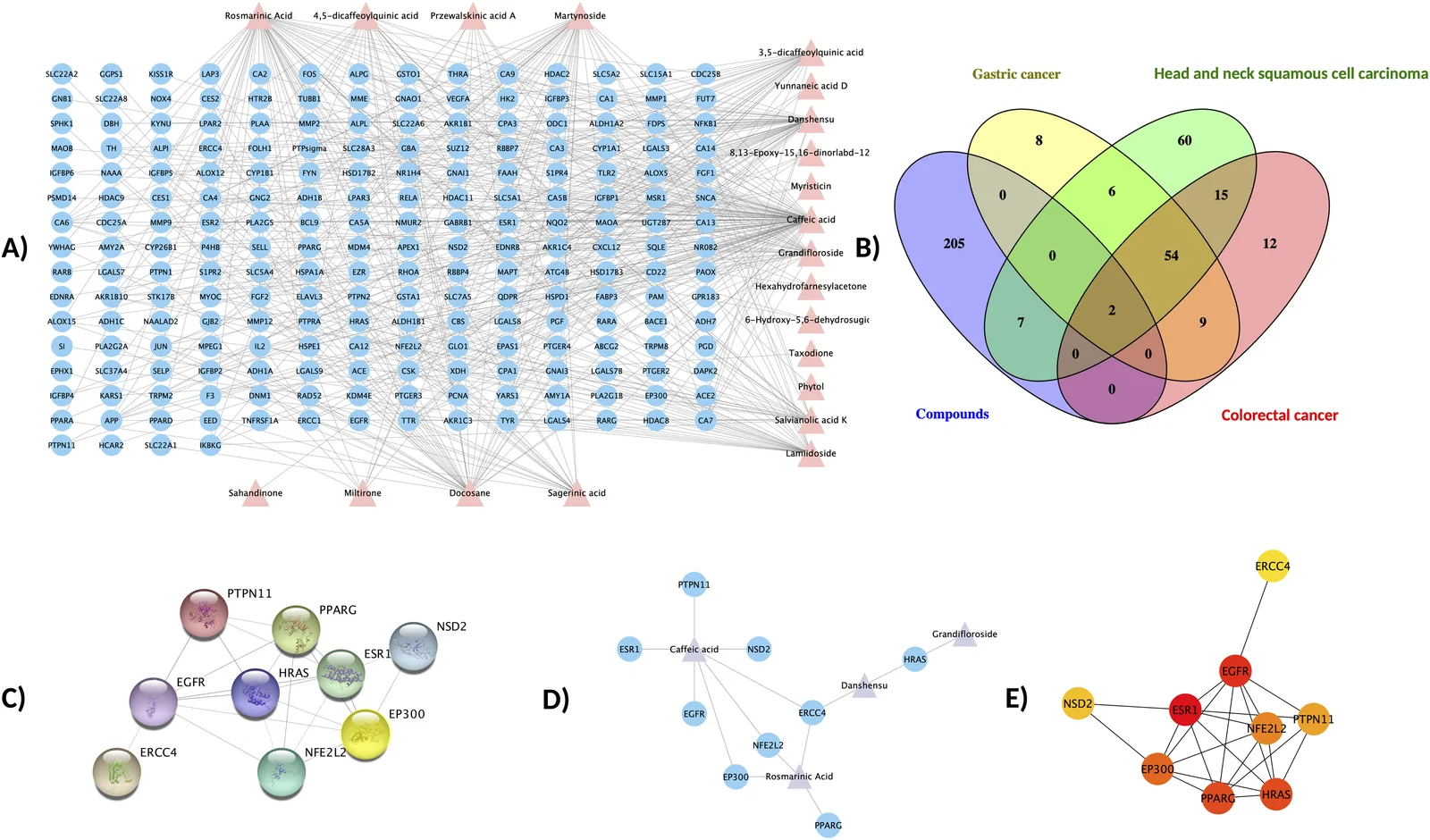
Integrated network analysis of *Salvia limbata*. (A) Compound–target interaction network. (B) Venn diagram showing overlapping targets between compounds and gastric, head and neck squamous cell, and colorectal cancers. (C) Protein–protein interaction (PPI) network of common targets. (D) Compound–hub gene interaction subnetwork. (E) STRING-based hub gene network ranked by the MCC method.

### 3.9. GO and KEGG analysis

GO and KEGG enrichment analyses were performed to investigate the potential biological functions of the identified targets. GO terms were classified into biological process (BP), cellular component (CC), and molecular function (MF). Based on *P* values, the top 10 enriched terms in each GO category and the top 20 KEGG pathways were selected (Fig. 8). A total of 84 significantly enriched terms (*P* < 0.05) were obtained for the 9 potential targets of *Salvia limbata*, including 35 BPs, 11 CCs, 16 MFs, and 22 KEGG pathways. In the BP category, the targets were mainly associated with the positive regulation of transcription by RNA polymerase II, the regulation of DNA-templated transcription, the response to estrogen, and the positive regulation of the ERK1 and ERK2 cascade. In the CC category, the enriched terms were primarily nucleoplasm, nucleus, chromatin, and a protein-containing complex. For MF, the targets were mainly involved in transcription coregulator binding, chromatin binding, DNA binding, transcription coactivator binding, and STAT family protein binding (Fig. 8A). KEGG pathway analysis indicated that the targets were mainly enriched in cancer-related pathways, particularly “Pathways in cancer,” “Breast cancer,” and “Prostate cancer,” as well as in signaling pathways, such as “JAK-STAT signaling pathway,” “Ras signaling pathway,” and “Estrogen signaling pathway” (Fig. 8B).

**Fig. 8:**
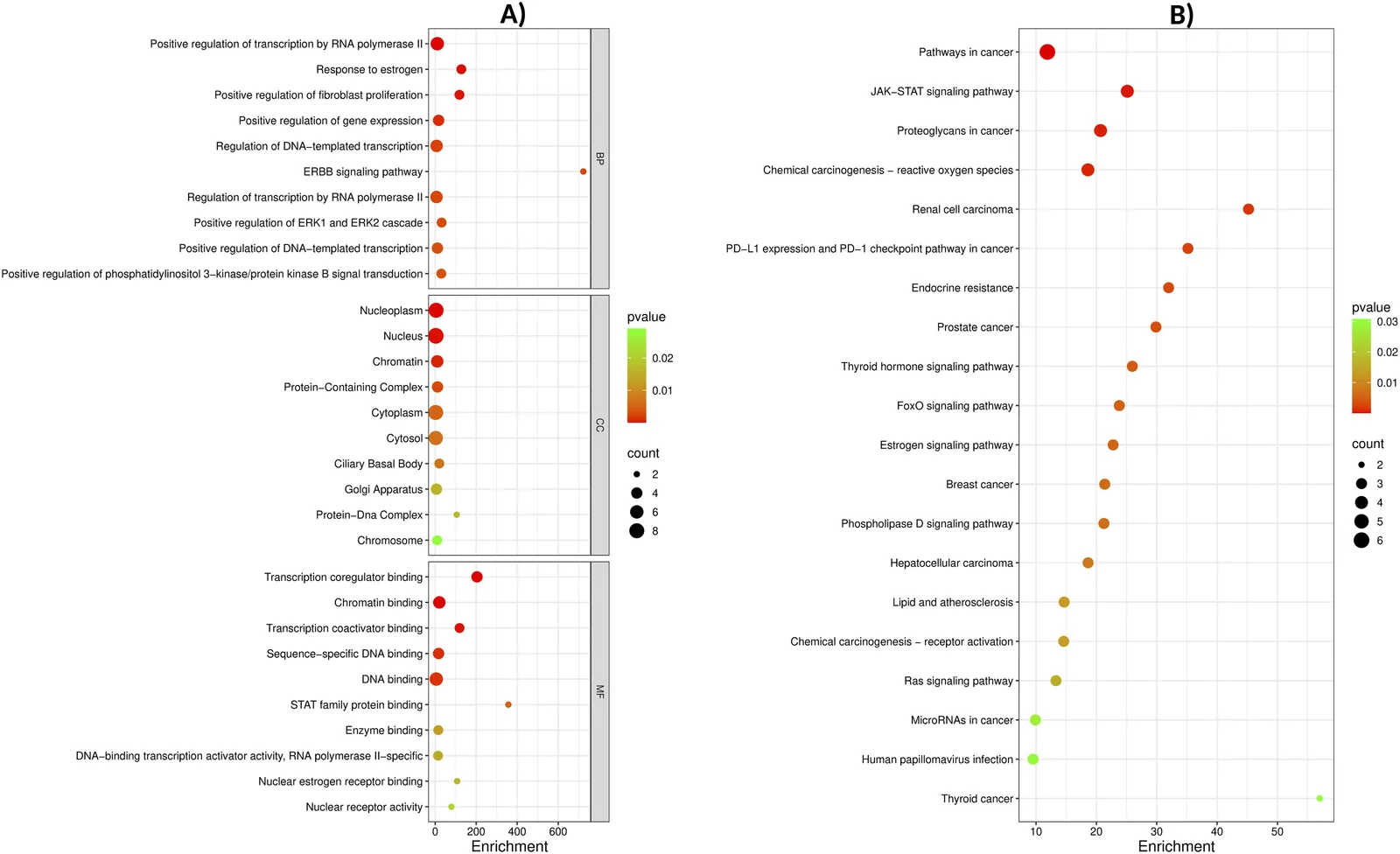
GO and KEGG enrichment analysis. (A) Top enriched GO terms. (B) Top enriched KEGG pathways.

### 3.10. Pathway-target-component network analysis based on MCC-derived ligand–protein interactions

The compound-target-pathway network constructed using MCC-prioritized proteins showed that ligand distribution was not uniform across the network (Fig. 7D). Caffeic acid had the highest number of direct protein connections, interacting with ESR1, NSD2, PTPN11, EGFR, ERCC4, and NFE2L2. This placed it at the structural center of the network. Rosmarinic acid was the second most connected compound, binding to EP300, NFE2L2, PPARG, and ERCC4. Both compounds shared ERCC4 and NFE2L2 as common targets, indicating partial overlap in their interaction profiles. Danshensu interacted mainly with ERCC4 and showed indirect linkage toward HRAS, whereas grandifloroside displayed a single but specific association with HRAS. Therefore, the interaction pattern reflected the concentration of activity within a limited group of metabolites rather than broad distribution across all compounds. For gastric and colorectal cancer categories, only HRAS and EGFR were retained as MCC-ranked targets, and ligand associations were limited accordingly. In the head and neck squamous cell carcinoma network, additional proteins expanded the interaction framework, with ERCC4 acting as a connective node between multiple ligands and HRAS-related signaling components. The recurrence of HRAS and EGFR across disease groups indicated their stable positioning within the network structure. Overall, the network highlighted a restricted set of ligand–protein interactions dominated by caffeic acid and rosmarinic acid. The clustering of associations around ERCC4, HRAS, EGFR, and NFE2L2 provided a focused set of protein–ligand pairs for subsequent docking and molecular dynamics investigations.

### 3.11. Protein–ligand interaction

The selected phytochemicals showed strong and consistent binding tendencies across the examined oncogenic targets, indicating favorable fitting within crucial catalytic and functional regions. Notably, strong interactions were seen with HRAS and EP300, reflecting stable ligand binding within their active sites. EGFR, NSD2, and PPARγ also displayed high binding affinities. The ligands also demonstrated favorable binding affinities toward AChE and BChE, suggesting promising inhibitory potential against cholinesterases. Comparable interactions were also observed with amylase and α-glucosidase, indicating possible dual activity relevant to carbohydrate metabolism. In contrast, binding affinities toward hCA I and hCA II were comparatively moderate, reflecting differences in active-site architecture and interaction patterns. The selected compounds demonstrated energetically favorable binding affinities toward UL26 protease and pUL15-CTD, supporting stable and thermodynamically plausible complex formation within these viral targets. The docking scores and detailed protein–ligand interaction profiles for these targets are summarized in Table S3 and briefly discussed below.

#### Cancer targets

##### HRAS

A strong docking score of −10.3 kcal mol^−1^ was obtained for 3,5-dicaffeoylquinic acid against HRAS. In addition to hydrogen bonds with Gly13, Gly15, Ser17, Ala18, Glu31, and Thr35, the ligand formed electrostatic interactions near the phosphate-binding loop (P-loop). Multiple hydrophobic contacts with residues such as Ile21 and Val29, together with salt bridges with Lys16 and Lys117, contributed to stabilization (Fig. 9A). The combined polar and hydrophobic interactions suggested potential interference with GTP-binding and conformational switching dynamics.^116^

**Fig. 9:**
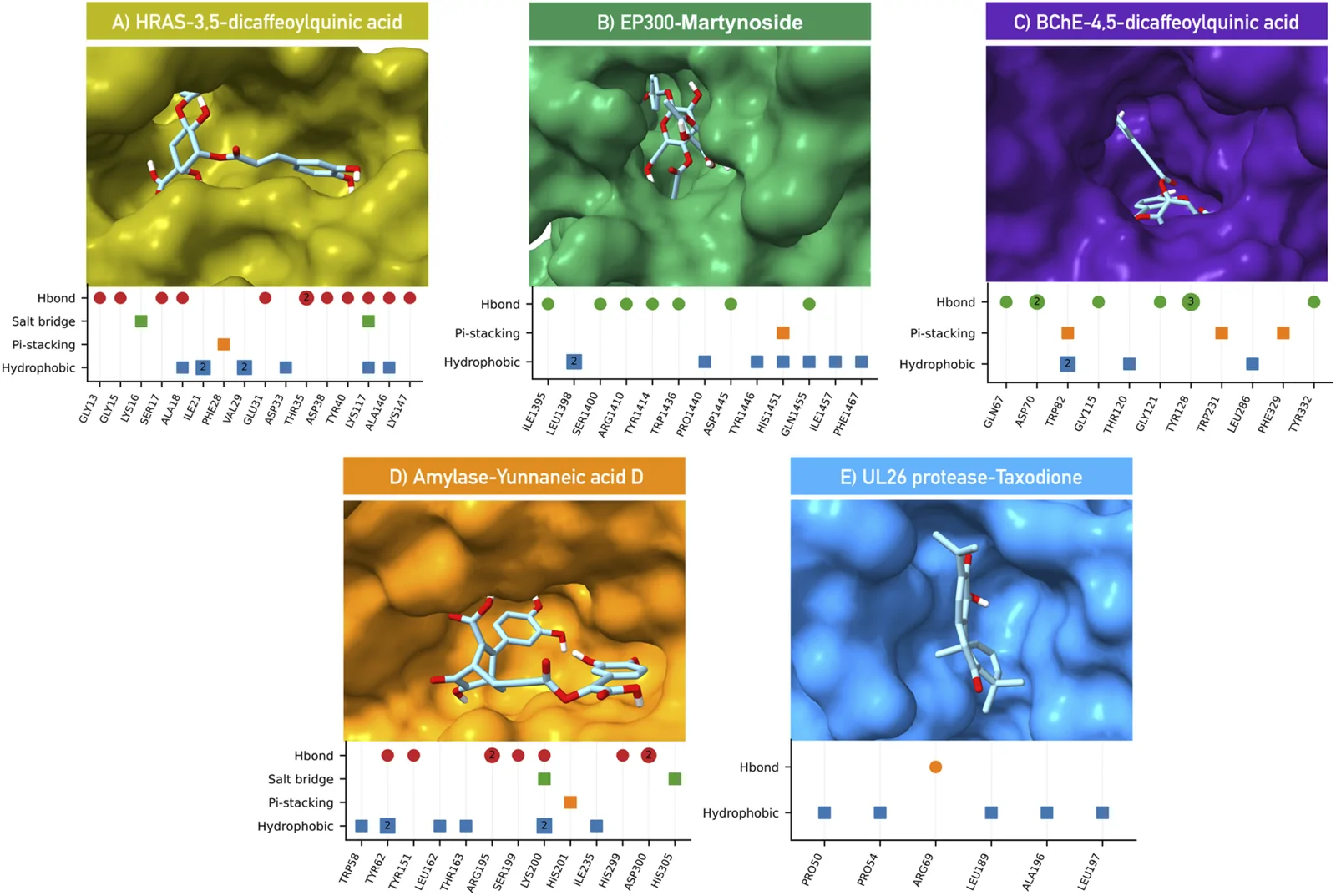
Protein–ligand interaction maps of selected complexes: (A) HRAS–3,5-dicaffeoylquinic acid. (B) EP300–martynoside; (C) BChE–4,5-dicaffeoylquinic acid; (D) amylase–yunnaneic acid D; and (E) Ul26 protease–taxodione.

##### EP300

Martynoside exhibited a strong docking score of −10.3 kcal mol^−1^ against EP300. The interaction network was anchored by multiple hydrogen bonds with Ile1395, Ser1400, Arg1410, Tyr1414, Trp1436, Asp1445, and Gln1455, suggesting strong polar stabilization within the catalytic pocket. Hydrophobic contacts with Leu1398, Pro1440, Tyr1446, His1451, Gln1455, Ile1457, and Phe1467 contributed to effective pocket enclosure and steric complementarity.^117^ Notably, a π-stacking interaction with His1451 further reinforced aromatic stabilization (Fig. 9B).

##### PPARγ

4,5-Dicaffeoylquinic acid displayed a docking score of −9.9 kcal mol^−1^ against PPARγ. Hydrogen bonds with Arg288, Tyr327, Glu343, and His449 were complemented by π–π interactions with Phe363 and π–alkyl contacts with Leu330 and Ile341 within the ligand-binding domain. Additionally, electrostatic interactions with Arg288 reinforced anchoring at the activation helix interface.^118^

##### NSD2

3,5-Dicaffeoylquinic acid docked into NSD2 with a score of −9.4 kcal mol^−1^. Besides hydrogen bonding with Phe266, Ala270, Glu272, and Phe277, the ligand exhibited π–π stacking interactions with aromatic Phe residues within the methyltransferase domain. Electrostatic stabilization involving Glu272 and hydrophobic packing with adjacent aliphatic residues further enhanced binding complementarity. The network of polar, aromatic, and van der Waals interactions suggested the stable occupation of the catalytic groove.

##### EGFR

Yunnaneic acid D demonstrated a docking score of −9.3 kcal mol^−1^ within the ATP-binding cleft of EGFR, indicating strong predicted affinity. In addition to hydrogen bonding with Met769 (hinge region), Arg817, Asn818, and Asp831, the ligand established π–alkyl interactions with Leu694 and Val702, contributing hydrophobic stabilization along the adenine-binding pocket. A π–π stacking interaction with Phe699 further reinforced aromatic complementarity within the catalytic site. Moreover, van der Waals contacts with surrounding residues enhanced steric accommodation, collectively supporting a stable ATP-competitive binding mode.

#### Enzyme targets

##### AChE

The strongest binder, 8,13-epoxy-15,16-dinorlabd-12-ene (−9.7 kcal mol^−1^), was stabilized predominantly through multiple hydrophobic interactions with Trp86, a central aromatic residue of the catalytic anionic subsite. The repeated contacts with Trp86 indicated tight packing within the hydrophobic gorge, favoring other hydrophobic interactions with Tyr124, Tyr337, Phe338, and Tyr341. This deep hydrophobic accommodation suggested the effective obstruction of the substrate access channel, consistent with inhibition through the steric occupation of the active-site cavity.^101,119^

##### BChE

The top-ranked ligand, 4,5-dicaffeoylquinic acid (−10.4 kcal mol^−1^), established multiple hydrogen bonds with Gln67, Asp70, Gly115, Gly121, and Tyr332. The hydrogen bonding with Asp70 suggested electrostatic stabilization near the peripheral anionic site, while interactions with Gly115 and Gly121 indicated backbone-mediated hydrogen bonding within the catalytic gorge. Contact with Tyr332 contributed additional stabilization through polar interactions (Fig. 9C), collectively promoting strong anchoring across both peripheral and active-site regions.^120^

##### α-Amylase

Yunnaneic acid D (−9.9 kcal mol^−1^) formed an extensive hydrogen-bonding network with Tyr62, Tyr151, Arg195, and Ser199 within the catalytic cleft. The hydrogen bonds with Arg195 likely involved electrostatic and directional stabilization through its guanidinium group, while interactions with Tyr151 and Tyr62 suggested additional stabilization *via* hydroxyl-mediated hydrogen bonding. The engagement of Ser199, located near the catalytic residues (Fig. 9D), indicated direct positioning within the active site, supporting a competitive inhibitory mechanism reinforced by polar complementarity.^121^

##### α-Glucosidase

3,5-Dicaffeoylquinic acid (−8.9 kcal mol^−1^) was stabilized by hydrogen bonds with Tyr246, Gln247, Lys283, Leu344, and Ala345. The hydrogen bonding with Lys283 likely involved electrostatic interactions between the ligand's hydroxyl groups and the ε-amino group of lysine, enhancing binding strength. Contacts with Tyr246 and Gln247 supported positioning within the substrate-binding pocket, while interactions with Leu344 and Ala345 suggested additional stabilization through backbone hydrogen bonding and hydrophobic packing within the catalytic region.^122^

##### Tyrosinase

8,13-Epoxy-15,16-dinorlabd-12-ene (−7.7 kcal mol^−1^) interacted mainly *via* hydrophobic contacts with Phe197, His208, and Val218. The hydrophobic packing against Phe197 supported van der Waals stabilization within the active pocket, while interaction with His208, positioned near the catalytic copper-binding environment, suggested potential interference with substrate orientation. Contact with Val218 further reinforced stabilization through nonpolar interactions within the enzyme's catalytic vicinity.^123^

##### hCA I

The highest affinity ligand, 3,5-dicaffeoylquinic acid (−8.8 kcal mol^−1^), formed hydrogen bonds with Ser65 and Gln92, indicating stabilization within the active-site cavity. The interaction with Ser65 likely involved side-chain hydroxyl-mediated hydrogen bonding, while Gln92 may contribute through amide-based hydrogen bonding. These polar contacts suggested proper orientation within the catalytic pocket, potentially perturbing substrate positioning.^124^

##### hCA II

In hCA II, 3,5-dicaffeoylquinic acid (−9.1 kcal mol^−1^) established a dense hydrogen-bonding network involving Tyr7, Asn67, Glu69, Gln92, Glu106, and Thr199. Notably, hydrogen bonding with Thr199, a residue directly involved in substrate orientation and proton transfer, provided strong support for catalytic interference. Additional interactions with Glu69 and Glu106 suggested electrostatic stabilization, while Asn67 and Gln92 contributed amide-mediated hydrogen bonding, collectively forming a multi-point anchoring system within the active-site region.^124,125^

#### Herpes virus targets

The UL26 protease exhibited favorable binding affinities with taxodione, achieving a docking score of −7.8 kcal mol^−1^ (RMSD 0.7 Å), indicative of stable complex formation within the catalytic region. Taxodione formed a hydrogen bond with Arg69, while hydrophobic contacts were established with Pro50, Pro54, Leu189, Ala196, and Leu197. These interactions suggested the deep accommodation of the diterpenoid scaffold within the hydrophobic substrate-binding cavity. The predominance of nonpolar interactions indicated that steric complementarity and van der Waals stabilization were major contributors to complex formation.^126,127^ Similarly, martynoside demonstrated a docking score of −7.0 kcal mol^−1^ (RMSD 1.0 Å) and formed hydrogen bonds with Leu49, Val73, and Gly185. These polar interactions were supported by hydrophobic contacts involving Val74, Asp75, and Glu192, reinforcing ligand stabilization within the catalytic cleft. The coexistence of hydrogen bonding and hydrophobic enclosure suggested a balanced interaction profile, potentially interfering with proteolytic maturation processes.^128^ Sahandinone displayed a highly stable binding pose (RMSD ≈ 0.0 Å; −6.6 kcal mol^−1^), with interactions dominated by hydrophobic contacts involving Pro54, Leu189, Ala193, Ala196, and Leu197. The clustering of interactions around Leu189–Leu197 highlighted this region as a key hydrophobic hotspot within the UL26 protease active site. Collectively, these findings indicated that the diterpenoid-type compounds and phenylethanoid glycosides present in the antiviral-active extracts may interfere with viral proteolytic processing.^129^ For the pUL15-CTD DNA-packaging domain, martynoside achieved a docking score of −7.2 kcal mol^−1^ (RMSD 1.3 Å). Hydrogen bonds were observed with Phe542, Arg668, Leu669, Gln670, and Thr671, while hydrophobic interactions and π-stacking were primarily mediated by Phe542. The repeated engagement of Phe542 underscored its central role in ligand stabilization within the internal cavity. Taxodione (−7.0 kcal mol^−1^; RMSD 1.1 Å) exhibited hydrophobic interactions predominantly with Phe542, accompanied by π-stacking interactions involving the same aromatic residue. This interaction profile indicated that ligand affinity was largely governed by nonpolar pocket complementarity and aromatic stacking within the binding cavity.^130^ Likewise, sahandinone (−6.8 kcal mol^−1^; RMSD 0.1 Å) formed hydrophobic contacts with Arg517, Phe542, Val667, and Tyr676, with additional π-associated stabilization involving Phe542. The recurrent involvement of Phe542 and Tyr676 suggested that aromatic residues played a decisive role in ligand accommodation within the pUL15-CTD domain.

Taken together, the stable and residue-specific interactions observed for martynoside, taxodione, and sahandinone within both UL26 protease and pUL15-CTD provided a structurally coherent explanation for the inhibition of viral replication. The simultaneous targeting of proteolytic maturation and genome packaging was consistent with the strong reduction in infectious viral titer and the more modest reduction in viral DNA levels observed *in vitro*.

### 3.12. Dynamic profile of protein–ligand complexes

#### Conformational stability

The RMSD profiles showed distinct stability patterns among the complexes. C1, C2, and C3 displayed relatively low and steady deviations (∼0.3–0.6 nm) throughout the 100 ns simulation, indicating structurally stable systems. In contrast, C4 underwent a noticeable conformational change around ∼15–20 ns, followed by stabilization at a high RMSD plateau (∼3 nm) before relaxing toward ∼1 nm after ∼50 ns. This suggested an early structural rearrangement, followed by equilibration. C5 demonstrated the highest instability, with multiple sharp spikes reaching ∼5–6 nm, especially after ∼50 ns and again near ∼80–100 ns (Fig. 10A). These sudden deviations pointed to significant conformational transitions or potential ligand-induced destabilization events.^131^

**Fig. 10:**
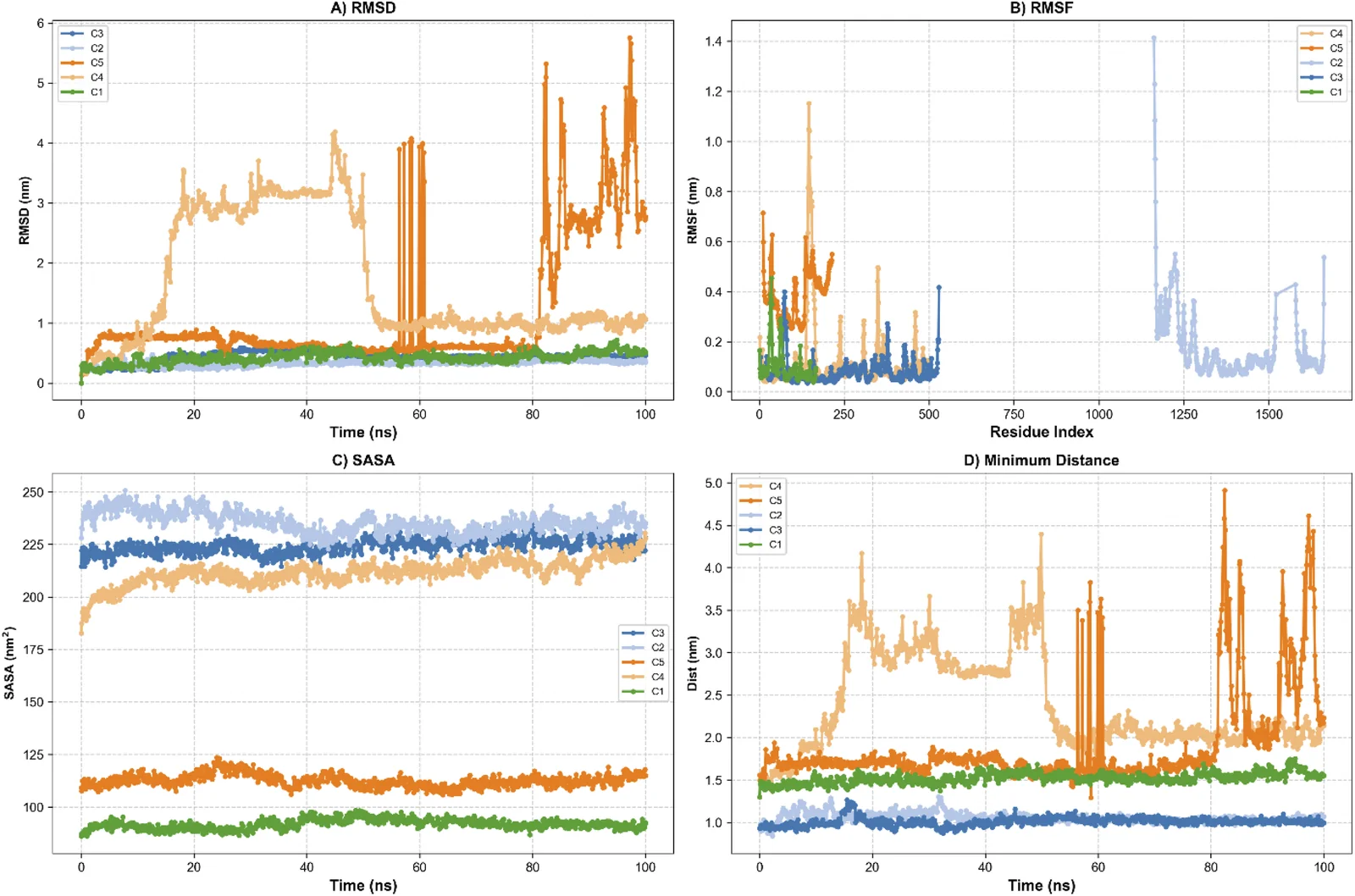
Structural and dynamic stability analysis of selected complexes *via* a 100 ns MD simulation: HRAS–3,5-dicaffeoylquinic (C1); EP300–martynoside (C2); BChE–4,5-dicaffeoylquinic acid (C3); amylase–yunnaneic acid D (C4); UL26 protease–taxodione (C5). (A) RMSD profiles show stable convergence for C1, C2, and C3, while C4 and especially C5 exhibit pronounced fluctuations. (B) RMSF analysis indicates limited residue flexibility overall, with localized higher fluctuations in C2 and C5. (C) SASA trends remain relatively steady for C1–C3, whereas C4 and C5 display moderate variations. (D) Minimum distance analysis confirms sustained ligand–protein proximity in C1–C3, while C4 and C5 show intermittent increases, suggesting comparatively weaker binding stability.

#### Residual fluctuations

The RMSF profiles highlighted residue-level flexibility. For C1 and C3, fluctuations remained low across most residues (<0.3–0.4 nm), indicating restricted backbone motion and stable local environments.^132^ C4 and C5 showed localized spikes, suggesting flexible loop regions or surface-exposed segments, which contributed to increased mobility.^133^ However, these fluctuations appeared to be region-specific rather than globally distributed. C2 presents a pronounced peak in a terminal region, reaching ∼1.4 nm (Fig. 10B), indicating significant localized flexibility. Importantly, the rest of the structure remained comparatively stable, implying that flexibility was confined rather than systemic.

#### Surface accessibility and compactness

SASA profiles revealed insights into compactness and solvent exposure. C1 maintained the lowest and most stable SASA (∼85–95 nm^2^), indicating a compact structure with minimal solvent exposure.^134^ C3 and C2 remained relatively stable (∼220–240 nm^2^), reflecting consistent structural packing over time. Slight oscillations represented normal dynamic fluctuations rather than unfolding events. C4 showed a gradual rise early in the simulation, aligning with the RMSD-observed conformational change, and then stabilized. C5 remained moderately stable (∼110–120 nm^2^) with minor oscillations (Fig. 10C), suggesting no significant unfolding despite RMSD spikes.

#### Intermolecular distance

The minimum distance plots reflected ligand–protein proximity stability. C1, C2, and C3 maintained consistent distances (∼1.0–1.6 nm), supporting persistent ligand binding throughout the simulation. C4 showed a notable increase in distance at ∼15–50 ns, corresponding to its RMSD shift, before returning to a more stable range.^135^ This aligned with a conformational rearrangement rather than permanent dissociation. C5 demonstrated multiple sharp distance increases (up to ∼5 nm), particularly after 50 ns and near 80–100 ns (Fig. 10D). These spikes strongly suggested transient ligand displacement or partial unbinding events.^136^

#### Hydrogen bond dynamics

C1 maintained a relatively consistent hydrogen bonding (H-bond) pattern throughout the simulation, fluctuating mainly between 3 and 6 H-bonds, with occasional peaks up to 7–8. Although brief drops occurred, the overall profile suggested persistent polar interactions (Fig. 11A). The sustained mid-range H-bond count supported stable ligand anchoring,^137^ consistent with the stable RMSD and distance trends observed.

**Fig. 11:**
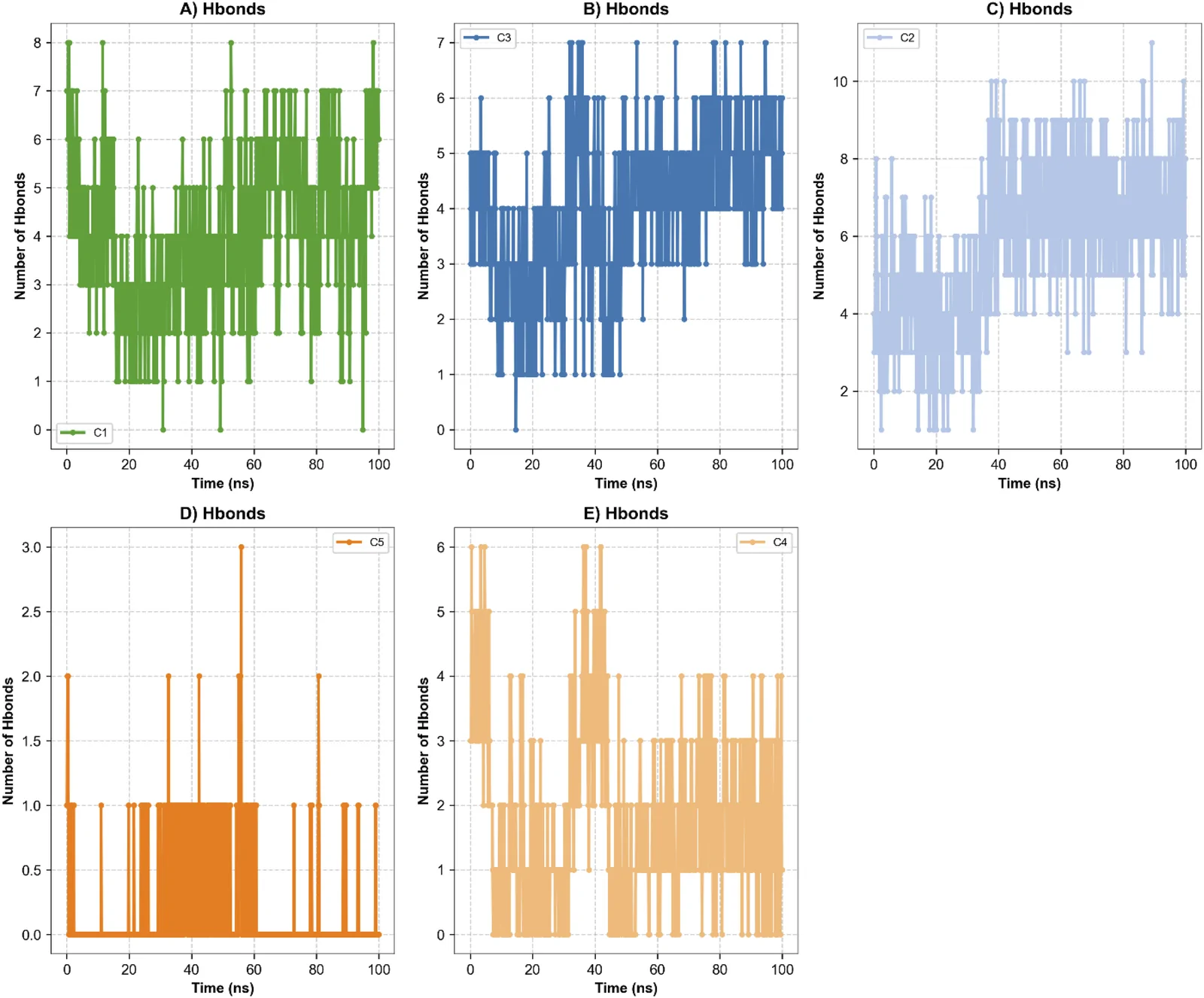
Time evolution of hydrogen bonds during 100 ns MD simulations for (A) HRAS-3,5-dicaffeoylquinic acid (C1), (B) EP300–martynoside (C2), (C) BChE-4,5-dicaffeoylquinic acid (C3), (D) amylase–yunnaneic acid D (C4), and (E) UL26 protease–taxodione (C5). C2 exhibits the highest and most persistent hydrogen-bond occupancy, followed by C3 and C1. C4 shows moderate but fluctuating hydrogen-bond interactions, whereas C5 displays fewer and more transient hydrogen bonds throughout the simulation.

C3 showed a gradual stabilization pattern. During the early phase (0–30 ns), fluctuations between 1 and 4 H-bonds were evident, but after ∼40 ns, the system consistently maintained 4–6 H-bonds (Fig. 11B). This shift indicated progressive binding optimization and stronger stabilization over time. The relatively steady mid-to-high H-bond count suggested durable polar interactions within the binding pocket.^138^

C2 exhibited the highest and most persistent H-bond occupancy among all systems. After ∼35–40 ns, the complex consistently maintained 6–9 H-bonds, with occasional peaks reaching 10–11 (Fig. 11C). This dense and sustained H-bonding network indicated a strong and stable polar engagement,^137,138^ reinforcing the structural stability observed in RMSD and minimum distance analyses.

C5 displayed a weak H-bonding behavior, with most of the simulations showing 0–1 H-bonds, and only rare transient peaks reaching up to 2–3 (Fig. 11D). The intermittent nature of these interactions suggested unstable or short-lived polar contacts.^139^ This profile aligned with the previously observed RMSD spikes and increased minimum distances, indicating reduced binding stability.^140^

Finally, C4 demonstrated moderate but unstable H-bonding. Early in the simulation (0–10 ns), 3–6 H-bonds were observed; however, a substantial decline occurred at ∼10–40 ns, with frequent zero-interaction periods. After ∼50 ns, the system partially recovered, fluctuating between 1 and 3 H-bonds (Fig. 11E). This pattern suggested an initial strong interaction phase, followed by rearrangement and partial re-stabilization.^141^

## 4. Conclusion

The *Salvia* genus has received considerable attention from the scientific community because of its richness in phenolic compounds, flavonoids, and terpenoids. There has been a growing need in recent years to look for new natural compounds with bioactive properties. The chemical compositions of the EO and extracts of *S. limbata* analyzed by GC-MS and HPLC-MS/MS, respectively, revealed that the plant was a rich source of EO and secondary metabolites. The EA extract had the highest antioxidant activity, followed by ethanol/water and water extracts. The EO had strong enzyme inhibitory activity, particularly against AChE, BChE, tyrosinase, lipase, and carbonic anhydrase. Network pharmacology analysis indicated that the major metabolites of *S. limbata* were associated with multiple biologically relevant targets, reflecting a multi-target interaction profile. Docking studies demonstrated the strong binding affinities of selected complexes (C1–C3) toward cancer-related proteins, metabolic enzymes, carbonic anhydrase isoforms, and viral proteins. Molecular dynamics simulations further confirmed the dynamic stability of these top-ranked complexes over 100 ns, with C2 and C3 exhibiting the most consistent structural behavior, supporting their potential as promising *in silico* drug candidates. The results of this study emphasized the high medicinal value of the plant, indicating its strong potential as an antioxidant and enzyme inhibitor; thus, further studies should explore its therapeutic uses and isolate new bioactive compounds for future drug development.

## Conflicts of interest

There are no conflicts to declare.

## Supplementary Material

RA-016-D6RA05137E-s001

## Data Availability

Data will be made available on request from the authors. Supplementary information (SI): Table S1. Grid center and size used for docking the most abundant phytochemicals in the extracts of *Salvia limbata* into the selected enzyme, cancer, and HBV targets. Table S2. Secondary metabolites in *S. limbata* extracts. Table S3. Docking scores and protein–ligand interaction of major compounds in extracts of *Salvia limbata* into the selected enzyme, cancer, and HBV target. Fig. S1. ESI-MS/MS spectrum of protocatechuic acid (2) at *m*/*z* 153.0193. Fig. S2. ESI-MS/MS spectrum of *p*-hydroxybenzoic (6) at *m*/*z* 137.0244. Fig. S3. ESI-MS/MS spectrum of chlorogenic acid (9) at *m*/*z* 353.0878. Fig. S4. ESI-MS/MS spectrum of caffeic acid (13) at *m*/*z* 179.0350. Fig. S5. ESI-MS/MS spectrum of *o*-coumaric acid (16) at *m*/*z* 163.0401. Fig. S6. ESI-MS/MS spectrum of ferulic acid (17) at *m*/*z* 193.0506. Fig. S7. ESI-MS/MS spectrum of 4,5-dicaffeoylquinic acid (19) at *m*/*z* 515.1195. Fig. S8. ESI-MS/MS spectrum of verbascoside (29) at *m*/*z* 623.1981. Fig. S9. ESI-MS/MS spectrum of isovitexin (41) at *m*/*z* 431.0984. Fig. S10. ESI-MS/MS spectrum of hyperoside (42) at *m*/*z* 463.0882. Fig. S11. ESI-MS/MS spectrum of isoquercitrin (43) at *m*/*z* 463.0882. Fig. S12. ESI-MS/MS spectrum of luteolin 7-*O*-glucoside (45) at *m*/*z* 447.0933. Fig. S13. ESI-MS/MS spectrum of apigenin 7-*O*-glucoside (46) at *m*/*z* 431.0984. See DOI: https://doi.org/10.1039/d6ra05137e.
