# Supplementary material for: Salvia limbata extracts and essential oil as a novel source of bioactive agents: metabolomic fingerprint, in vitro, in silico and network pharmacological perspectives
Source: RSC Adv. 2026 Sep 14;16(44):54922–49. doi: 10.1039/d6ra05137e (PMC13573238; doi:10.1039/d6ra05137e)
Supplement: Supplementary file 1 [file RA-016-D6RA05137E-s001.pdf]

Supplementary Information (SI) for RSC Advances.
This journal is © The Royal Society of Chemistry 2026

Analysis of the essential oils

The oils were analyzed by gas chromatography-flame ionization detector (GC-FID) and gas chromatography/mass spectrophotometry (GC/MS) techniques. GC/MS analysis was carried out by an Agilent 5975 GC-MSD system coupled to an Agilent 7890A GC (Agilent Technologies Inc., Santa Clara, CA). HP-Innowax FSC column (60x0.25 mm, 0.25 µm film thickness) was used with helium (purity 99.99%) as carrier gas (1.2 mL/minute). The experimental details are given in the supplemental materials.

GC oven temperature was kept at 60°C for 10 minutes (min) and programmed to 220°C at a rate of 4°C/min, and kept constant at 220°C for 10 min and then programmed to 240°C at a rate of 1 /min. The split ratio was used at 40:1. The injector temperature was adjusted to 250°C and mass spectra were recorded at 70 eV. Mass range was adjusted from 35 to 450 m/z. GC-FID analysis was carried out by simultaneous auto-injection using Agilent 7693A series auto sampler; 1 µL injections were affected.

GC analysis was carried out using an Agilent 7890A GC system. In order to obtain the same elution order with GC/MS, simultaneous triplicate injections were done by using the same column and same operational conditions. FID temperature was 300°C.

Identification of constituents was achieved on the basis of retention index (RI) determined by co-injection with reference to a homologous series of *n*-alkanes (C_8-C_{30}), under same experimental conditions. Further identifications were carried out by comparison of their mass spectra with those from NIST 05 and Wiley 8th version and home-made MS library built up from pure substances and components of known EOs, as well as by comparison of their RIs with literature values [1].

Table S1. Grid center and size used for docking the most abundant phytochemicals in the extracts of *Salvia Limbata* into the selected enzyme, cancer, and HBV targets

Target Protein	PDB ID	Center X, Y, Z (Å)	Grid Size X, Y, Z (Å)
AChE	7E3H	-54.44, 32.90, -28.65	25, 25, 25
BChE	6EQP	32.16, -16.33, 40.73	25, 25, 25
Amylase	2QV4	14.188, 48.964, 22.886	28, 28, 24
Glucosidase	7KBJ	-18.79, 2.18, 15.75	25, 25, 25
Tyrosinase	6QXD	21.81, 12.22, 91.40	25, 25, 25
hCAI	3LXE	-19.049, 36.885, 44.812	40, 40, 40
HCA-II	4IWZ	14.059, 4.757, 14.338	40, 40, 40
EGFR	1M17	25.126, 1.027, 51.326	64, 34, 28
EP300	7VHY	-20.433, -17.216, 8.376	114, 98, 82
ESR1	4ZNH	5.681, 1.756, 21.987	94, 64, 64
HRAS	1P2S	8.731, 35.93, 19.722	50, 30, 36
NSD2	6XCG	3.888, 15.858, 18.378	82, 82, 68
PPARγ	7E0A	44.501, -0.358, 86.52	76, 68, 86
SHP2	3B7O	17.069, 9.849, 56.550	112, 126, 108
UL15-CTD	4IOX	74.309, 35.477, -2.541	126, 76, 98
UL26 Protease	8BQ1	26.842, 11.926, 38.872	126, 88, 78

Table S2. Secondary metabolites in *S. limbata* extracts.

№	Identified/tentatively annotated compound	Molecular formula	Exact mass [M-H]⁻	MS/MS fragments	t_R (min)	Δ ppm	Level of confidence	Distribution
**Hydroxybenzoic, hydroxycinnamic, acylquinic acids and derivatives**								
**1.**	danshensu	$C_9H_{10}O_5$	197.0455	197.0448 (16.9), 179.0341 (70.6), 135.0439 (100), 123.0438 (78.3)	1.78	-3.536	D1	3,5,6,7,8,9,10
**2.**	protocatechuic acid[a]	$C_7H_6O_4$	153.0193	153.0182 (16.6), 109.0281 (100), 81.0331 (2.8)	2.04	-7.397	B	2,4,7,8,9,10
**3.**	caffeoyltartaric acid	$C_{13}H_{12}O_9$	311.0409	311.0424 (0.5), 179.0342 (73.5), 149.0080 (100), 135.0439 (46.4)	2.37	5.127	D1	5,6,10
**4.**	p-hydroxyphenyllactic acid[a]	$C_9H_{10}O_4$	181.0506	181.0498 (61.2), 163.0391 (100), 135.0440 (66.2), 119.0489 (43.8)	2.54	-4.762	B	1,2,3,4,5,8,9,10
**5.**	hydroxybenzoic acid	$C_7H_6O_3$	137.0244	137.0232 (100), 108 0203 (9.7), 93.0331 (13.9)	2.84	-8.592	D1	1,2,3,4,5,7,8,9,10
**6.**	*p*-hydroxybenzoic acid[a]	$C_7H_6O_3$	137.0244	137.0234 (9.4), 108.0203 (3.5), 93.0331 (100)	2.94	-7.133	B	1,3,4,5,7,8,9,10
**7.**	*p*-coumaric acid *O*-dihexoside	$C_{21}H_{28}O_{13}$	487.1457	487.1461 (60.0), 163.0391 (100), 145.0284 (91.0), 119.0490 (34.0), 323.0988 (4.2)	2.95	0.772	D1	5,10
**8.**	*p*-coumaroyltartaric acid	$C_{13}H_{12}O_8$	295.0459	295.0461 (1.5), 163.0390 (100), 135.0439 (0.5), 119.0489 (29.9)	3.11	0.371	D1	1,5,8
**9.**	chlorogenic acid[a]	$C_{16}H_{18}O_9$	353.0878	353.0878 (4.2), 191.0544 (100), 179.0343 (1.0), 161.0232 (1.6), 135.0439 (0.9)	3.20	0.070	B	5,10
**10.**	*p*-coumaric acid *O*-hexoside	$C_{15}H_{18}O_8$	325.0929	325.0924 (12.6), 163.0390 (100), 145.0284 (93.4), 119.0489 (76.2)	3.35	-1.479	D1	2,4,5,7,10
**11.**	4-caffeoylquinic acid[a]	$C_{16}H_{18}O_9$	353.0878	353.0879 (35.6), 191.0554 (100), 179.0343 (1.0), 161.0232 (1.6),	3.38	0.155	B	5,10

				135.0439 (51.1)				
**12.**	feruloyltartaric acid	$C_{14}H_{14}O_9$	325.0565	325.0566 (1.8), 193.0500 (100), 178.0263 (2.1), 161.0598 (6.3), 163.0390 (17.9), 149.0080 (4.9), 134.0361 (50.2), 133.0646 (2.4)	3.55	0.415	D1	2,4,5,10
**13.**	caffeic acid[a]	$C_9H_8O_4$	179.0350	179.0341 (20.4), 135.0439 (100), 117.0334 (0.7), 107.0488 (1.55)	3.57	-5.094	B	2,3,4,5,6,7,8,9,10
**14.**	*p*-coumaric acid *O*-hexoside isomer	$C_{15}H_{18}O_8$	325.0929	325.0938 (9.6), 265.0718 (100), 235.0611 (45.4), 205.0500 (59.3), 163.0392 (77.5), 145.0283 (79.7), 119.0489 (65.4)	3.82	2.735	D1	2,4,5,7,10
**15.**	ferulic acid *O*-hexoside	$C_{16}H_{20}O_9$	355.1035		3.76	0.182	D1	2,5,10
**16.**	*o*-coumaric acid[a]	$C_9H_8O_3$	163.0401	163.0390 (9.2), 135.0436 (0.9), 119.0489 (0.9)	4.55	-6.792	B	2,3,4,5,10
**17.**	ferulic acid[a]	$C_{10}H_{10}O_4$	193.0506	193.0499 (17.7), 178.0263 (29.8), 165.0181 (0.2), 149.0596 (12.3)134.0360 (100)	5.41	-3.585	B	5,9,10
**18.**	3,5-dicaffeoylquinic acid	$C_{25}H_{24}O_{12}$	515.1195	515.1187 (21.5), 353.0882 (89.0), 191.0554 (100), 179.0341 (37.8), 161.0234 (4.5), 135.0439 (1.5)	5.88	-1.532	B	3,5,10
**19.**	4,5-dicaffeoylquinic acid[a]	$C_{25}H_{24}O_{12}$	515.1195	515.1196 (93.1), 353.0880 (50.5), 335.0807 (0.6), 191.0553 (32.7), 179.0341 (62.5), 173.0446 (100), 135.0439 (59.9)	6.23	0.137	B	3,5,10
**Caffeic acid oligomers**								
**20.**	przewalskinic acid A	$C_{18}H_{14}O_8$	357.0616	357.0621 (53.1), 313.0721 (28.1), 269.0821 (70.1), 203.0344 (30.2), 175.0388 (6.2), 159.0440 (26.8), 161.0231 (3.5), 135.0438 (5.6), 133.0281 (9.9), 123.0436 (1.5), 109.0281 (100)	4.43	1.343	D1	5,10

**21.**	yunnaneic acid D	$C_{27}H_{24}O_{12}$	539.1195	539.1198 (85.7), 495.1349 (0.6), 359.0775 (8.1), 341.0666 (0.8), 315.0878 (3.9), 297.0768 (20.8), 271.0971 (4.5), 197.0448 (19.3), 179.0341 (29.6), 161.0234 (46.0), 135.0439 (100), 123.0438 (7.0), 109.0281 (8.5)	4.95	0.465	D1	5,10
**22.**	rosmarinic acid *O*-hexoside	$C_{24}H_{26}O_{13}$	521.1301	521.1327 (44.6), 503.1557 (27.5), 359.0775 (33.4), 197.0447 (18.5), 179.0340 (24.90), 161.0234 (100), 135.0439 (64.4), 133.0283 (16.8), 123.0444 (4.2)	5.15	5.039	D1	5,8,9,10
**23.**	rosmarinic acid *O*-hexuronide	$C_{24}H_{24}O_{14}$	535.1093	535.1096 (61.8), 359.0778 (10.0), 203.0344 (0.5), 197.0450 (19.3), 179.0342 (9.9), 161.0234 (100), 135.0439 (19.5), 133.0283 (24.5), 123.0438 (5.4)	5.16	0.433	D1	5
**24.**	yunnaneic acid F	$C_{29}H_{26}O_{14}$	597.1250	597.1252 (56.2), 553.1342 (2.7), 509.1459 (2.7), 491.1362 (3.7), 329.1032 (7.4), 311.0930 (14.5), 267.1028 (16.7), 197.0448 (18.), 179.0343 (13.6), 135.0439 (100)	5.23	0.337	D1	5,10
**25.**	sagerinic acid	$C_{36}H_{32}O_{16}$	719.1618	719.1624 (40.7), 539.1207 (1.0), 359.0776 (12.6), 323.0557 (0.2), 341.0663 (2.4), 297.0769 (0.6), 281.0440 (0.4), 255.0663 (1.2), 197.0449 (18.9), 179.0342 (11.2), 161.0234 (100), 135.0439 (22.7), 133.0283 (27.7)	5.94	0.907	D1	3,4,5,8,9,10
**26.**	salvianolic acid K	$C_{27}H_{24}O_{13}$	555.1144	555.1150 (10), 537.1055 (2.1), 493.1145 (31.2), 401.0880 (9.5), 359.0775 (78.3), 295.0609 (4.9), 197.0449 (24.2), 179.0341 (15.3),	6.30	1.038	D1	5,6,7,9,10

				161.0234 (94.8)				
**27.**	rosmarinic acid[a]	$C_{18}H_{16}O_8$	359.0772	359.0774 (14.3), 197.0448 (30.5), 179.0341 (12.2), 161.0233 (100), 133.0282 (21.3), 135.0439 (15.9), 109.0281 (0.2)	6.34	0.416	B	2,3,4,5,6,7,8,9,10
**28.**	methylrosmarinate	$C_{19}H_{18}O_8$	373.0929	373.0927 (8.7), 197.30448 (43.8), 193.0502 (3.7), 179.0340 (22.7), 175.0391 (50.1), 161.0235 (24.6), 135.0439 (100)	7.47	-0.404	D1	2,3,4,5,10
**Phenylethanoid, iridoid, and lignan glycosides**								
**29.**	verbascoside[a]	$C_{29}H_{36}O_{15}$	623.1981	623.01984 (66.6), 461.1681 (9.1), 161.0234 (100), 133.0283 (33.8)	5.50	0.347	C	5
**30.**	martynoside	$C_{31}H_{40}O_{15}$	651.2294	651.2301 (100), 475.1815 (0.9), 329.1235 (0.8), 193.0499 (13.5), 175.0392 (98.5), 167.0704 (2.7), 161.0444 (1.1), 160.0156 (63.9), 152.0467 (1.2), 149.0598 (2.9), 134.0361 (15.9), 132.0205 (21.1), 132.0205 (21.1)	7.26	1.008	D1	2,3,4,5,8,9,10
**31.**	caffeoyl-hexosyl-iridoid I	$C_{26}H_{32}O_{14}$	567.1719	567.1705 (94.9), 549.1614 (21.4), 341.0885 (0.9), 281.0667 (13.4), 251.0565 (6.9), 221.0449 (9.5), 193.0499 (5.7), 179.0341 (22.9), 161.0234 (100), 135.0439 (21.4), 133.0283 (45.6)	4.46	-2.572	D1	5,8,9,10
**32.**	caffeoyl-hexosyl-iridoid II	$C_{25}H_{30}O_{13}$	537.1614	537.1617 (89.1), 519.1496 (16.4), 341.0869 (1.5), 281.0667 (13.3), 251.0559 (6.6), 221.0450 (6.7), 195.0649 (1.9), 177.0548 (5.2), 161.0234 (100), 179.0341 (21.7), 149.0599 (2.6), 133.0283 (37.5)	4.51	0.588	E	4,5,8,10

**33.**	syringaresinol *O*-hexoside	$C_{28}H_{36}O_{13}$	579.2083	579.2068 (4.1), 417.1558 (100), 402.1326 (9.9), 387.1089 (2.4), 359.1107 (1.2), 211.0011 (1.0), 181.0497 (61.0), 166.0263 (32.9), 151.0025 (12.6)	6.07	-2.632	D1	5,8,9,10
**Flavonoids**								
**34.**	quercetin 3-O-hexuronylhexoside	$C_{27}H_{28}O_{18}$	639.1203	639.1208 (100), 463.0890 (19.7), 301.0354 (74.8), 300.0277 (35.6), 271.0235 (1.5), 151.0027 (19.7), 107.0124 (8.1)	3.53	0.787	D1	5
**35.**	naringenin 6,8-di*C*- hexoside	$C_{27}H_{32}O_{15}$	595.1668	595.1670 (100), 475.1229 (2.1), 457.1170 (1.2), 415.1055 (8.2), 355.0828 (39.4), 385.0935 (34.0), 151.0028 (1.2), 119.0491 (12.2), 107.0126 (1.2)	3.65	0.246	D1	5
**36.**	apigenin 6,8-*C*-hexosyl hexoside	$C_{27}H_{30}O_{15}$	593.1512	593.1520 (100), 503.1201 (4.3), 473.1094 (12.5), 413.0873 (2.6), 383.0770 (15.7), 323.0575 (0.7), 297.0765 (9.4), 117.0334 (2.4)	4.05	1.326	D2	5
**37.**	luteolin *O*-dihexuronide	$C_{27}H_{26}O_{18}$	637.1046	637.1049 (100), 285.0406 (71.9), 151.0025 (5.0), 133.0138 (0.8), 107.0124 (2.6)	4.37	0.444	D1	4,5,10
**38.**	luteolin 7-*O*-hexuronyl-hexoside	$C_{27}H_{28}O_{17}$	487.0882	623.1258 (65.9), 447.0925 (1.7), 285.0406 (100), 284.0327 (2.4), 151.0026 (4.4), 133.0282 (7.1), 107.0124 (2.5)	4.64	0.670	D1	5
**39.**	eriodictyol 7-*O*-rutinoside	$C_{27}H_{32}O_{15}$	595.1668	595.1671 (76.6), 287.0562 (80.7), 151.0025 (100), 135.0439 (55.9), 109.0280 (1.7), 107.0124 (22.1)	5.13	0.347	D2	2,3,5
**40.**	apigenin 7-O-hexuronyl-hexoside	$C_{27}H_{28}O_{16}$	607.1305	607.1306 (38.3), 269.0457 (100), 225.0555 (2.0), 151.0036 (0.6), 117.0333 (5.0), 107.0122 (1.8)	5.15	0.267	D1	5

**41.**	isovitexin[a]	$C_{21}H_{20}O_{10}$	431.0984	431.0984 (100), 341.0669 (23.0), 323.0565 (4.4), 311.0564 (78.7), 283.0613 (25.9), 269.0461 (4.1), 117.0333 (11.0), 161.0234 (4.5), 135.0436 (2.1)	5.17	-0.023	B	2,3,10
**42.**	quercetin 3-*O*-galactoside (hyperoside)[a]	$C_{21}H_{20}O_{12}$	463.0882	463.0884 (100), 301.0347 (36.8), 300.0274 (79.3), 271.0250 (42.3), 255.0298 (17.7), 243.0301 (10.4), 227.0347 (2.3), 211.0392 (0.8), 178.9976 (2.5), 151.0025 (5.7), 121.0282 (0.7), 107.0125 (2.6)	5.20	0.390	B	2,3,4,5,8
**43.**	quercetin 3-*O*-glucoside (isoquercitrin)[a]	$C_{21}H_{20}O_{12}$	463.0882	463.0884 (100), 301.0348 (37.5), 300.0276 (69.3), 271.0250 (34.3), 255.0305 (14.5), 243.0301 (8.7), 227.0347 (1.3), 178.9974 (2.7), 151.0305 (14.5), 107.0122 (2.7)	5.31	0.455	B	2,3,4,5,8
**44.**	luteolin 7-*O*-hexuronide	$C_{21}H_{18}O_{12}$	461.0725	461.0727 (59.8), 285.0406 (100), 151.0046 (5.2), 133.0282 (9.9), 107.0123 (2.6)	5.39	0.241	B	2,3,4,5,9,10
**45.**	luteolin 7-*O*-glucoside[a]	$C_{21}H_{20}O_{11}$	447.0933	447.0933 (100), 285.0404 (85.1), 284.0327 (34.1), 227.0342 (1.7), 151.0025 (3.8), 133.0283 (3.9), 107.0124 (2.5)	5.41	0.079	B	2,3,4,8
**46.**	apigenin 7-*O*-glucoside[a]	$C_{21}H_{20}O_{10}$	431.0984	431.0982 (100), 269.0450 (25.4), 268.0378 (52.6), 211.0393 (1.6), 151.0026 (2.7), 117.0332 (1.4), 107.0125 (2.2), 149.0230 (1.2)	6.10	-0.371	B	2,3,4,5
**47.**	apigenin 7-*O*-hexuronide	$C_{21}H_{18}O_{12}$	445.0776	445.0764 (26.2), 269.0457 (100), 225.0559 (1.6), 175.0239 (14.8), 151.0028 (1.5), 149.0235 (1.4), 117.0332 (6.6), 107.0125 (2.4)	6.15	-2.661	D1	1,3,4,5,9,10
**48.**	luteolin 7-*O*-malonylhexoside	$C_{17}H_{26}O_{19}$	533.0996	533.0986 (0.6), 489.1041 (100), 447.0955 (1.6), 327.0513 (1.4),	6.20	-1.729	D1	5

				285.0406 (60.7), 284.0329 (32.3), 227.0343 (0.9), 151.0029 (3.9), 133.0279 (4.1), 107.0124 (2.9)				
**49.**	hispidulin *O*-hexoside	$C_{22}H_{22}O_{11}$	461.1089	461.1087 (100), 299.0560 (9.1), 284.0319 (4.9), 283.0250 (16.1), 255.0300 (55.7), 117.0330 (0.8)	6.31	-0.574	D1	2,3,4,5
**50.**	chrysoeriol *O*-hexuronide	$C_{22}H_{20}O_{12}$	475.0882	475.0874 (96.3), 300.0595 (8.7), 299.0561 (100), 284.0325 (63.9), 151.0023 (1.6), 117.0180 (1.1), 107.0120 (1.1)	6.35	-1.682	D1	2,3,4,5,10
**51.**	luteolin 7-O-acetylhexuronide	$C_{23}H_{20}O_{13}$	503.0831	503.0836 (43.9), 443.0601 (0.2), 399.0724 (0.7), 381.0617 (0.2), 353.0637 (0.3), 339.0511 (0.8), 297.0386 (0.3), 285.0406 (100), 257.0455 (0.3), 151.0024 (4.7), 133.0282 (9.9), 107.0124 (2.7)	6.48	1.006	D1	2,3,4,5
**52.**	methylhypolaetin	$C_{16}H_{12}O_7$	315.0510	315.0513 (100), 300.0276 (50.5), 271.0240 (0.8), 243.0295 (1.6), 227.0345 (2.3), 136.9868 (8.8), 133.0282 (3.6), 109.9995 (0.6)	6.85	0.775	D1	2
**53.**	apigenin 7-*O*-acetylhexoside	$C_{23}H_{22}O_{11}$	473.1089	473.1090 (100), 413.0877 (0.5), 311.0557 (0.4), 269.0450 (24.8), 268.0379 (55.6), 211.0391 (1.6), 151.0029 (3.1), 117.0332 (1.7), 107.0125 (3.3)	6.93	0.159	D1	5
**54.**	apigenin 7-*O*-acetylhexuronide	$C_{23}H_{20}O_{12}$	487.0882	487.0883 (10.7), 269.0458 (100), 151.0026 (2.5), 117.0332 (5.8), 107.0123 (2.7)	7.26	0.310	D1	3,4,2
**55.**	luteolin 7-O-acetylhexuronide isomer	$C_{23}H_{20}O_{13}$	503.0831	503.0835 (90.1), 443.0621 (20.2), 399.0723 (1.5), 381.0628 (0.5), 339.0522 (0.3), 297.0408 (1.2), 285.0406 (100), 257.0462 (0.4), 151.0025 (5.4), 133.0283 (9.7),	7.27	1.006	D1	2,3,4,5,10

				107.0124				
**56.**	chrysoeriol O-malonylpentoside	$C_{24}H_{22}O_{13}$	517.0988	517.0989 (61.0), 413.0893 (0.5), 300.0597 (10.4), 299.0563 (100), 284.0328 (51.3), 256.0376 (8.1), 227.0349 0.7), 175.0236 (0.6), 151.0021 (2.3), 139.0025 (1.9), 113.0230 (31.2), 107.0126 (1.3)	7.48	0.225	D1	3,4,5
**57.**	luteolin[a]	$C_{15}H_{10}O_6$	285.0405	285.0405 (100), 151.0025 (5.8), 117.0332 (19.1), 107.0124 (4.7), 149.0233 (5.2)	7.62	0.241	B	1,2,3,4,5,7,8,9
**58.**	quercetin[a]	$C_{15}H_{10}O_7$	301.0354	301.0354 (100), 283.0244 (0.4), 273.0406 (2.6), 257.0462 (1.4), 178.9977 (21.7), 161.0237 (0.9), 151.0025 (43.0), 121.0124 (12.9), 107.0124 (14.6)	7.62	0.080	B	2,3,8,9
**59.**	methylisoscutellarein	$C_{16}H_{12}O_6$	299.0561	299.0561 (100), 284.0326 (61.8), 255.0289 (0.9), 227.0347 (3.4), 163.0024 (0.3), 136.9868 (14.4), 117.0333 (2.9)	7.87	0.029	D1	2,3
**60.**	apigenin 7-*O*-acetylhexuronide	$C_{23}H_{20}O_{12}$	487.0882	487.0884 (63.6), 427.0670 (49.3), 383.0772 (0.6), 309.0409 (1.5), 281.0452 (1.7), 269.0457 (100), 151.0020 (2.0), 117.0332 (7.7), 107.0124 (2.5)	8.06	0.433	D1	5
**61.**	cirsiliol[a]	$C_{17}H_{14}O_7$	329.0667	329.0668 (68.8), 314.0434 (100), 29920199 (22.5), 271.0250 (51.1), 285.0377 (0.3), 243.0300 (3.6), 227.0343 (1.3), 199.0391 (4.3), 151.0488 (0.6), 133.0279 (0.6)	8.29	0.468	B	2
**62.**	narinngenin[a]	$C_{15}H_{12}O_5$	271.0612	271.0612 (100), 151.0024 (64.6), 119.0488 (54.5), 107.0125 (23.7)	8.55	0.197	B	7,8,9
**63.**	apigenin[a]	$C_{15}H_{10}O_5$	269.0455	269.0456 (100), 151.0025 (5.8), 117.0332 (19.1), 107.0124 (4.7),	8.65	0.161	B	2,3,4,5,7,8,9

				149.0233 (5.2)				
**64.**	chrysoeriol[a]	$C_{16}H_{12}O_6$	299.0561	299.0561 (100), 284.0327 (91.5), 256.0376 (18.6), 227.0343 (2.7), 151.0026 (3.6), 107.0125 (2.2)	8.94	0.029	B	2,3,4,5,9
**65.**	jaceosidin[a]	$C_{17}H_{14}O_7$	329.0667	329.0668 (100), 314.0433 (38.9), 299.0197 (33.4), 271.0252 (4.6), 243.0293 (4.2), 227.0343 (4.2), 215.0335 (0.6), 199.0393 (13.7), 164.9815 (2.1), 163.0026 (3.6), 136.9867 (1.0), 133.0282 (15.6)	9.17	0.286	B	2,3,6,7,8,9
**66.**	luteolin 7-methyleter/diosmetin	$C_{16}H_{12}O_6$	299.0561	299.0560 (100), 284.0325 (44.6), 256.0373 (4.0), 151.0026 (2.9), 107.0124 (2.3), 133.0282 (4.2)	10.28	-0.272	D1	2,3,4
**67.**	pectolinarigenin	$C_{17}H_{14}O_6$	313.0718	313.0719 (100), 298.0482 (51.9), 283.0249 (51.8), 269.0457 (3.4), 255.0298 (12.5), 227.0345 (4.4), 211.0394 (1.6), 183.0442 (4.2), 178.09979 (0.8), 163.0026 (13.4), 135.0075 (5.1), 117.0332 (12.0)	10.37	0.347	C	2,3,4,5,7,8,9
**68.**	eupatilin[a]	$C_{18}H_{16}O_7$	343.0823	343.0824 (80.6), 328.0590 (100), 313.0356 (22.5), 298.0121 (17.7), 285.0405 (5.7), 270.0171 (25.5), 257.0083 (1.6), 133.0275 (1.4)	10.67	0.128	B	2,3,4,5,7,8,9
**69.**	genkwanin[a]	$C_{16}H_{12}O_5$	283.0611	283.0612 (100), 268.0378 (58.4), 239.0347 (3.8), 240.0422 (4.1), 211.0394 (2.0), 151.0025 (2.9), 117.0331 (3.9), 107.0124 (2.5)	11.62	0.082	B	2,3,4,7,8,9
**№**	**Identified/tentatively annotated compound**	**Molecular formula**	**Exact mass [M+H]⁺**		**t_R (min)**	**Δ ppm**	**Level of confide nce**	**Distribution**
**Diterpenes**								

**70.**	taxodione	$C_{20}H_{26}O_3$	315.1954	315.1951 (84.6), 297.1848 (6.6), 273.1471 (2.1), 259.1324 (8.9), 245.1170 (79.5), 217.1220 (4.3), 203.0702 (18.9), 83.0861 (100)	14.22	-2.822	D2	1,2,3,4,5,6,7,8,9, 10
**71.**	20-deoxocarnosol	$C_{20}H_{28}O_3$	317.2111	317.2108 (100), 243.1387 (0.3), 229.1229 (1.2), 215.1065 (0.4), 197.0959 (0.3), 191.0701 (0.3), 173.0600 (0.6)	15.60	-0.950	D2	2,3,6,7,8,9,10
**72.**	sugiol	$C_{20}H_{28}O_2$	301.2162	301.2156 (100), 259.1689 (3.6), 241.1582 (0.7), 227.1426 (0.6), 213.1273 (3.9), 205.1223 (2.7), 185.0959 (1.9), 171.0803 (4.1), 163.0752 (5.9)	15.98	-1.881	D2	2,3,6,7,8,9,10
**73.**	6-hydroxysalvonolone/carnosol	$C_{20}H_{26}O_4$	331.1904	331.1897 (100), 295.1690 (7.5), 285.1844 (16.7), 271.1323 (15.8), 261.1118 (86.8), 289.1431 (2.4), 267.1741 (3.7), 249.1126 (1.9), 225.1273 (3.8), 83.0861 (97.9)	16.74	-3.817	D2	1,2,3,4, 6,7,8,9,10
**74.**	viridone	$C_{21}H_{28}O_3$	329.2111	329.2105 (63.6), 297.1844 (6.5), 259.1325 (69.8), 244.1094 (3.8), 231.1384 (4.9), 189.0905 (2.9), 174.0676 (1.9), 83.0861 (100)	17.07	-3.523	D2	2,3,6,7,8,9,10
**75.**	miltirone	$C_{19}H_{22}O_2$	283.1693	283.1689 (100), 265.1583 (10.4), 250.1350 (0.9), 237.1639 (2.6), 223.1115 (16.8), 241.1217 (7.6), 209.0952 (0.6), 178.0791 (0.2)	19.47	-3.372	D2	6,7,8,9
**76.**	sahandinone	$C_{20}H_{24}O_2$	297.1849	297.1845 (30.2), 279.1738 (41.7), 264.1509 (2.5), 249.1266 (3.2), 239.1427 (100), 221.0958 (17.6), 197.0958 (16.9), 179.0853 (20.2)	20.78	-3.079	D2	1,3,4,5,6,7,8,9,10
**77.**	dehydrosugiol	$C_{20}H_{26}O_2$	299.2006	299.2002 (100), 281.1890 (69.7), 257.1533 (22.7), 243.1378 (3.6), 229.1221 (59.5), 187.0753 (17.3)	21.91	-1.326	D2	1,2,3,4,5,6,7,8,9, 10

a-compare to reference standard Confidence level: B: confirmed structure except for one or more stereochemical aspects; C: tentative identification matched with a standard compound, match of at least tR, MS and MS/MS with an actual authentic standard analyzed in parallel, preferably supported by other online data; D: Tentative identification based on libraries, model compounds etc.; D1: relatively reliable evidence; D2: relatively poor evidence; E: tentative candidate or tentative identification of metabolite class [16]; [a]-compare to reference standard;

Table S3. Docking scores and protein-ligand interaction of major compounds in extracts of *Salvia Limbata* into the selected enzyme, cancer, and HBV target

Compound	Target	PDB ID	Binding energy (kcal/mol)	RMSD (Å)	Interaction type and binding site residues
3,5-dicaffeoylquinic acid	AChE	7E3H	-7.5	0.5	Hbond: GLN:71;GLN:71;TRP:86; GLY:126;TYR:337<br>Hydrophobic: TYR:72;ASP:74;TRP:86;TYR:124; PHE:338;TYR:341<br>Salt bridge: HIS:447
4,5-dicaffeoylquinic acid	AChE	7E3H	-8.0	0.9	Hbond: ASP:74;ASN:87;GLY:120;GLY:120; GLY:121;GLY:121;GLY:122;TYR:133; TYR:133;TYR:337<br>Hydrophobic: ASP:74;TRP:86;TRP:86;TRP:86;TYR:124; PHE:297;PHE:338;PHE:338;TYR:341<br>Pi-stacking: TRP:86
6-Hydroxy-5,6-dehydrosugiol	AChE	7E3H	-8.3	0.7	Hbond: GLY:121;GLY:122;SER:203<br>Hydrophobic: TRP:86;TRP:86;TRP:86;TYR:124; TRP:286;PHE:297;TYR:337;TYR:337; PHE:338;PHE:338;PHE:338;HIS:447
8,13-Epoxy-15,16-dinorlabd-12-ene	AChE	7E3H	-9.7	0.0	Hydrophobic: TRP:86;TRP:86;TRP:86;TRP:86; TYR:124;TYR:337;PHE:338;TYR:341;TYR:341
Caffeic acid	AChE	7E3H	-7.5	1.0	Hbond: GLY:120;TYR:124;TYR:133;TYR:133;

					TYR:337;TYR:341<br>Hydrophobic: TRP:86;TRP:86<br>Pi-stacking: TRP:86
Danshensu	AChE	7E3H	-7.7	0.8	Hbond: ASP:74;ASP:74;GLY:120;<br>TYR:124;GLU:202;TYR:337<br>Hydrophobic: TRP:86;TYR:337<br>Pi-stacking: TRP:86;TRP:86
Docosane	AChE	7E3H	-7.0	5.9	Hydrophobic:<br>ASP:74;TRP:86;TRP:86;TRP:86;<br>TRP:86;TRP:86;TRP:86;TYR:124;T<br>YR:124;<br>PHE:297;PHE:297;TYR:337;TYR:33<br>7;<br>TYR:337;PHE:338;TYR:341
Grandifloroside	AChE	7E3H	-5.7	0.2	Hbond:<br>ASP:74;GLY:120;GLY:121;GLY:12<br>2;<br>ALA:127;GLU:202;SER:203<br>Hydrophobic:<br>TRP:86;TRP:86;TRP:86<br>Pi-stacking:<br>TYR:337;TYR:341;HIS:447<br>Salt bridge: HIS:447
Lamiidoside	AChE	7E3H	-7.4	0.4	Hbond:<br>TYR:72;GLY:120;GLY:121;TYR:12<br>4;<br>SER:125;TYR:133;TYR:133;TYR:33<br>7;HIS:447<br>Hydrophobic:<br>TYR:72;ASP:74;TRP:86;TYR:124;T<br>YR:337;PHE:338<br>Salt bridge: HIS:447
Miltirone	AChE	7E3H	-9.0	0.0	Hydrophobic:<br>TRP:86;TRP:86;TRP:86;TRP:286;<br>PHE:297;TYR:337;PHE:338;PHE:33<br>8

					Pi-stacking: TYR:341;HIS:447
Myristicin	AChE	7E3H	-6.9	0.1	Hbond: TYR:337<br>Hydrophobic:<br>PHE:297;PHE:338;TYR:341<br>Pi-stacking: TRP:86
Phytol	AChE	7E3H	-3.9	0.5	Hbond: TYR:124<br>Hydrophobic:<br>ASP:74;TRP:86;TRP:86;TYR:124;<br>LEU:130;TYR:337;TYR:337;PHE:33<br>8;<br>PHE:338;PHE:338;TYR:341;TYR:34<br>1;HIS:447
Piperitenone oxide	AChE	7E3H	-7.5	0.0	Hbond: TYR:124<br>Hydrophobic:<br>TRP:86;TRP:86;TRP:86;TRP:86;TR<br>P:86;TYR:337
Przewalskinic acid A	AChE	7E3H	-7.0	0.6	Hbond:<br>ASN:87;GLY:120;TYR:124;TYR:13<br>3;<br>TYR:133;GLU:202<br>Hydrophobic:<br>TRP:86;TRP:86;TYR:337;TYR:337;<br>PHE:338;TYR:341<br>Salt bridge: HIS:447
Rosmarinic acid	AChE	7E3H	-9.6	0.8	Hbond:<br>ASP:74;GLY:121;GLY:122;TYR:13<br>3;<br>TYR:133;SER:203;TYR:337<br>Hydrophobic:<br>TRP:86;TRP:86;TYR:124;PHE:297;<br>PHE:297;PHE:338;PHE:338
Sagerinic acid	AChE	7E3H	-6.9	0.0	Hbond: GLY:121;GLY:122<br>Pi-stacking: HIS:447
Sahandinone	AChE	7E3H	-6.0	0.1	Hbond: TYR:124<br>Hydrophobic:<br>TRP:86;TRP:86;TRP:86;TYR:119;

					LEU:130;TYR:133;TYR:337;TYR:337; PHE:338;PHE:338;PHE:338;TYR:341; TYR:341;TYR:341;HIS:447
Salvianolic acid K	AChE	7E3H	-6.9	0.4	Hbond: ASP:74;TRP:86;ASN:87;GLY:120; GLY:121;GLY:122;TYR:124;GLY:126; TYR:133;GLU:202;GLU:202;ALA:204 Hydrophobic: TRP:86;TYR:124;PHE:297;PHE:297; PHE:338;TYR:341 Salt bridge: HIS:447
Taxodione	AChE	7E3H	-8.7	1.1	Hbond: TYR:124;SER:125 Hydrophobic: TRP:86;LEU:130;TYR:337;TYR:337; PHE:338;PHE:338;TYR:341;TYR:341 Pi-stacking: TRP:86
Viridone	AChE	7E3H	-7.6	0.2	Hbond: TYR:124;TYR:341 Hydrophobic: TYR:72;ASP:74; TRP:86;TRP:86;TYR:124; TYR:337;PHE:338
Yunnaneic acid D	AChE	7E3H	-9.1	0.4	Hbond: ASP:74;TRP:86;GLY:120;GLY:121; GLY:121;TYR:124;GLY:126;GLU:202; GLU:202;SER:203 Hydrophobic: ASP:74;TRP:86;TRP:86;TRP:86; TYR:337;TYR:337 Pi-stacking: HIS:447
3,5-dicaffeoylquinic	Amylase	2qv4	-8.7	0.3	Hbond:

acid					GLN:63;ARG:195;ARG:195;GLU:233;<br>GLU:233;GLU:233;HIS:299<br>Hydrophobic:<br>TRP:58;TRP:59;TYR:62;<br>TYR:62;LEU:162<br>Salt bridge: HIS:305;HIS:305
4,5-dicaffeoylquinic acid	Amylase	2qv4	-8.9	1.1	Hbond: GLN:63;ARG:195;<br>ARG:195;ASP:197;<br>ASP:197;ALA:198;GLU:233;HIS:299<br>Hydrophobic: TRP:58;TRP:59;<br>TYR:62;LEU:162;LEU:165<br>Pi-stacking: TYR:62<br>Salt bridge: HIS:305
6-Hydroxy-5,6-dehydrosugiol	Amylase	2qv4	-9.3	0.8	Hbond: THR:163;GLU:233<br>Hydrophobic:<br>TRP:58;TRP:58;TRP:59;TYR:62;<br>TYR:62;LEU:162;LEU:162;ILE:235
8,13-Epoxy-15,16-dinorlabd-12-ene	Amylase	2qv4	-8.5	0.0	Hydrophobic: TYR:62;TYR:62
Caffeic acid	Amylase	2qv4	-6.7	0.7	Hbond: ARG:195;HIS:299;ASP:300<br>Hydrophobic: TYR:62;TYR:62;<br>LEU:162;LEU:165
Danshensu	Amylase	2qv4	-6.6	0.1	Hbond:<br>GLN:63;ARG:195;ARG:195;ASP:197;<br>ALA:198;HIS:299<br>Hydrophobic: TYR:62;TYR:62
Docosane	Amylase	2qv4	-5.0	4.1	Hydrophobic:<br>TRP:58;TRP:58;TRP:59;TRP:59;<br>TYR:62;TYR:62;LEU:162;ILE:235
Grandifloroside	Amylase	2qv4	-8.6	1.1	Hbond:<br>ASN:53;ASN:53;GLN:63;HIS:101;<br>GLU:233;ASP:300<br>Hydrophobic:

					ILE:51;ILE:51;TRP:59; TRP:59;TRP:59; TRP:59;TYR:62;VAL:107;LEU:165 Salt bridge: HIS:305
Hexahydrofarnesylacetone	Amylase	2qv4	-6.0	0.0	Hbond: HIS:299 Hydrophobic: ILE:51;ILE:51;ILE:51; TRP:58;TRP:58; TRP:59;VAL:107;LEU:165
Lamiidoside	Amylase	2qv4	-8.6	1.1	Hbond: HIS:101;ASP:197;ASP:197; ALA:198; LYS:200;GLU:233;ILE:235;HIS:305 Hydrophobic: LYS:200;ILE:235;ILE:235 Pi-stacking: HIS:201 Salt bridge: HIS:305;HIS:305
Martynoside	Amylase	2qv4	-8.7	1.0	Hbond: GLN:63;GLY:104;LYS:200; ILE:235;ASP:300 Hydrophobic: TRP:59;TYR:62;LEU:162;LEU:165 Pi-stacking: HIS:201 Salt bridge: HIS:305
Miltirone	Amylase	2qv4	-8.9	0.1	Hbond: ASP:300 Hydrophobic: TRP:58;TRP:59;TYR:62;LEU:165 Pi-stacking: TRP:59
Myristicin	Amylase	2qv4	-5.7	0.0	Hbond: GLN:63 Hydrophobic: TRP:59;TYR:62
Phytol	Amylase	2qv4	-6.3	0.4	Hbond: ARG:195;ARG:195;HIS:299 Hydrophobic: ILE:51;TRP:59;TYR:62; VAL:107;LEU:162
Piperitenone oxide	Amylase	2qv4	-5.9	0.0	Hydrophobic: TRP:58;TRP:59;TYR:62
Przewalskinic acid A	Amylase	2qv4	-8.7	0.2	Hbond: TYR:62;ARG:195;ASP:197;GLU:233

					Hydrophobic:<br>TRP:58;TRP:59;TYR:62<br>Salt bridge: HIS:305
Rosmarinic acid	Amylase	2qv4	-8.3	1.0	Hbond:<br>GLN:63;THR:163;ARG:195;GLU:23<br>3;<br>HIS:299;ASP:300;HIS:305<br>Hydrophobic:<br>TRP:58;TRP:59;TYR:62;<br>LEU:162;LEU:165
Sagerinic acid	Amylase	2qv4	-5.5	0.0	Hbond: HIS:101
Sahandinone	Amylase	2qv4	-8.2	2.5	Hydrophobic:<br>TRP:58;TRP:59;TYR:62;<br>LEU:165;TRP:357
Salvianolic acid K	Amylase	2qv4	-9.1	0.8	Hbond:<br>TRP:59;GLN:63;TYR:;ARG:195;<br>ASP:197;ALA:198;LYS:200;<br>HIS:201;GLU:233;HIS:299;HIS:305<br>Hydrophobic:<br>TRP:58;TYR:62;LYS:200;ILE:235<br>Pi-stacking: TYR:62;HIS:201
Taxodione	Amylase	2qv4	-8.3	5.4	Hbond: ASP:300<br>Hydrophobic:<br>TRP:59;TYR:62;LEU:162;LEU:165;<br>ILE:235;ASP:300
Viridone	Amylase	2qv4	-8.7	1.0	Hbond: ASP:197;ALA:198;HIS:201<br>Hydrophobic:<br>TYR:62;TYR:151;LEU:162;<br>LEU:165;LYS:200;ILE:235
Yunnaneic acid D	Amylase	2qv4	-9.9	1.0	Hbond:<br>TYR:62;TYR:151;ARG:195;ARG:19<br>5;<br>SER:199;LYS:200;HIS:299;ASP:300<br>;ASP:300<br>Hydrophobic:<br>TRP:58;TYR:62;TYR:62;LEU:162;

					THR:163;LYS:200;LYS:200;ILE:235<br>Pi-stacking: HIS:201<br>Salt bridge: LYS:200;HIS:305
3,5-dicaffeoylquinic acid	BChE	6EQP	-9.8	1.1	Hbond:<br>ASP:70;GLY:115;GLY:115;THR:120;<br>THR:120;TYR:128;SER:198<br>Hydrophobic: ASP:70;TRP:82<br>Pi-stacking: TRP:82;PHE:329
4,5-dicaffeoylquinic acid	BChE	6EQP	-10.4	0.4	Hbond:<br>GLN:67;ASP:70;ASP:70;GLY:115;<br>GLY:121;TYR:128;TYR:128;TYR:128;TYR:332<br>Hydrophobic:<br>TRP:82;TRP:82;THR:120;<br>LEU:286<br>Pi-stacking:<br>TRP:82;TRP:231;PHE:329
6-Hydroxy-5,6-dehydrosugiol	BChE	6EQP	-9.3	0.9	Hbond:<br>GLY:116;GLY:117;GLU:197;PRO:285<br>Hydrophobic:<br>TRP:82;TRP:82;TRP:82;VAL:288;<br>PHE:329
8,13-Epoxy-15,16-dinorlabd-12-ene	BChE	6EQP	-8.6	0.1	Hydrophobic:<br>TRP:82;TRP:82;TRP:82;TRP:82;<br>ALA:328;TYR:332;TRP:430;TYR:440
Caffeic acid	BChE	6EQP	-6.6	2.8	Hbond:<br>GLY:115;GLY:115;TYR:128;TYR:128<br>Hydrophobic: TRP:82;TRP:82<br>Pi-stacking: TRP:82
Danshensu	BChE	6EQP	-6.8	1.0	Hbond: GLY:78;TRP:82;GLY:115;<br>TYR:128;TRP:430<br>Hydrophobic: TRP:82

					Pi-stacking: TRP:82;TRP:82
Docosane	BChE	6EQP	-6.2	1.8	Hydrophobic: TRP:82;TRP:82; TRP:231;TRP:231; LEU:286;VAL:288;ALA:328;PHE:329; TYR:332;PHE:398
Grandifloroside	BChE	6EQP	-9.3	0.0	Hbond: ASP:70;GLY:115;GLY:115;THR:120; THR:120;GLU:197;PRO:285;SER:287 Hydrophobic: TRP:82;PHE:329; TYR:332 Pi-stacking: TRP:82
Hexahydrofarnesylacetone	BChE	6EQP	-1.6	8.8	Hbond: GLY:121 Hydrophobic: ILE:69;ASP:70;TRP:82;PRO:84; THR:120; TRP:231;TRP:231;LEU:286;ALA:328; PHE:329;HIS:438
Lamiidoside	BChE	6EQP	-9.7	1.1	Hbond: ASP:70;GLY:115;GLY:116;GLY:117; GLU:197;SER:198;LEU:286 Hydrophobic: TRP:82;TYR:332 Pi-stacking: TRP:82;PHE:329
Martynoside	BChE	6EQP	-6.6	0.5	Hbond: ASP:70;ASP:70;TRP:82; TRP:82;GLY:117;THR:120;GLU:197; SER:198;LEU:286;TRP:430 Hydrophobic: PHE:329 Pi-stacking: TRP:82
Miltirone	BChE	6EQP	-9.4	0.0	Hbond: GLY:116;GLY:117;SER:198 Hydrophobic: TRP:231;LEU:286; VAL:288;PHE:329

					Pi-stacking: TRP:82
Myristicin	BChE	6EQP	-6.3	0.0	Hbond: ASP:70<br>Hydrophobic: TRP:82;TRP:82<br>Pi-stacking: TRP:82
Phytol	BChE	6EQP	-5.2	0.8	Hydrophobic: ASP:70;TRP:82;<br>TRP:82;GLN:119;<br>THR:120;TRP:231;TRP:231;LEU:28<br>6;<br>PHE:329;PHE:398;PHE:398
Piperitenone oxide	BChE	6EQP	-6.6	0.0	Hydrophobic:<br>TRP:82;TRP:82;TRP:82;TRP:82;<br>ALA:328;TYR:332;TRP:430
Przewalskinic acid A	BChE	6EQP	-9.7	1.1	Hbond:<br>GLY:78;TRP:82;GLY:115;PRO:285;<br>LEU:286;TRP:430<br>Hydrophobic: PHE:398<br>Pi-stacking:<br>TRP:82;TRP:231;TRP:231;<br>PHE:329;HIS:438
Rosmarinic acid	BChE	6EQP	-8.7	0.4	Hbond: ASP:70;ASP:70;SER:79;<br>TRP:82;ASN:83;TYR:128;<br>TYR:128;TRP:430<br>Hydrophobic: ASP:70;THR:120<br>Pi-stacking: TRP:82;TRP:82
Sagerinic acid	BChE	6EQP	-6.3	0.1	Pi-stacking: TRP:82;TRP:82
Sahandinone	BChE	6EQP	-8.3	0.0	Hbond: ASP:70<br>Hydrophobic:<br>TRP:231;TRP:231;VAL:288;<br>ALA:328;<br>PHE:329;TYR:332;PHE:398;<br>TRP:430;TYR:440
Salvianolic acid K	BChE	6EQP	-8.6	1.4	Hbond: GLN:119;SER:198;LEU:286;<br>SER:287;HIS:438<br>Hydrophobic:<br>TRP:82;TRP:82;TRP:82;TRP:82;<br>PHE:329;TYR:332

Taxodione	BChE	6EQP	-9.7	0.7	Hbond: GLY:117;SER:198<br>Hydrophobic:<br>TRP:82;TRP:82;TRP:82;TRP:231;<br>VAL:288;PHE:329;PHE:329<br>Pi-stacking: PHE:329
Viridone	BChE	6EQP	-9.1	0.7	Hbond: GLY:117;SER:198<br>Hydrophobic:<br>TRP:82;TRP:82;TRP:82;TRP:231;<br>LEU:286;PHE:329;PHE:329
Yunnaneic acid D	BChE	6EQP	-9.2	0.1	Hbond:<br>GLY:78;TRP:82;GLY:117;THR:120;<br>TYR:128;TRP:430<br>Hydrophobic: TRP:82;VAL:288;<br>PHE:329<br>Pi-stacking: TRP:82<br>Salt bridge: HIS:438
3,5-dicaffeoylquinic acid	Glucosid ase	7KBJ	-8.9	4.5	Hbond:<br>TYR:246;GLN:247;LYS:283;LEU:34 4;<br>ALA:345;TYR:348;TYR:348<br>Hydrophobic: LEU:252;<br>LEU:344;PHE:350<br>Salt bridge: ARG:352;ARG:352
4,5-dicaffeoylquinic acid	Glucosid ase	7KBJ	-8.8	5.7	Hbond:<br>PHE:237;PHE:237;GLY:240;ARG:2 67;<br>ARG:267;PHE:271;LYS:283;SER:29 0;<br>TYR:291;TYR:291;ARG:334<br>Hydrophobic: PHE:237;LEU:252;<br>LEU:252;GLU:270<br>Salt bridge: LYS:283;ARG:352
6-Hydroxy-5,6-dehydrosugiol	Glucosid ase	7KBJ	-8.0	0.8	Hbond: ARG:334<br>Hydrophobic: ILE:257;TYR:291;<br>TYR:291;LEU:344
8,13-Epoxy-15,16-	Glucosid	7KBJ	-7.8	0.0	Hydrophobic:

dinorlabd-12-ene	ase				TYR:291;ILE:327;PHE:330;TRP:331;<br>LEU:344;PHE:350
Caffeic acid	Glucosidase	7KBJ	-6.1	0.4	Hbond:<br>GLU:248;LYS:283;TYR:291;ARG:352<br>Hydrophobic: LEU:252
Danshensu	Glucosidase	7KBJ	-6.0	0.7	Hbond:<br>GLN:247;GLN:247;LYS:283;<br>ARG:352;ARG:352<br>Hydrophobic: LEU:252
Docosane	Glucosidase	7KBJ	-5.2	8.5	Hydrophobic:<br>LEU:252;LEU:252;TYR:291;<br>PHE:330;TRP:331;TYR:348;PHE:350
Grandifloroside	Glucosidase	7KBJ	-6.3	0.7	Hbond:<br>TYR:246;GLU:248;GLY:264;<br>LYS:283;GLU:287;SER:290;ARG:334;<br>ARG:352;ARG:352<br>Hydrophobic: TYR:291;TYR:291<br>Salt bridge: ARG:352;ARG:352<br>Pi-cation: ARG:352
Hexahydrofarnesylacetone	Glucosidase	7KBJ	-5.1	0.0	Hbond: LYS:283<br>Hydrophobic:<br>LEU:252;ILE:257;PHE:330;<br>PHE:330;<br>ALA:345;TYR:348
Lamiidoside	Glucosidase	7KBJ	-7.3	3.8	Hbond:<br>GLY:240;GLN:247;GLY:253;<br>ARG:267;PHE:271;SER:290;<br>PHE:350;ARG:352<br>Hydrophobic:<br>PHE:237;LEU:252;GLU:270<br>Salt bridge: LYS:283;ARG:352
Martynoside	Glucosid	7KBJ	-6.0	4.1	Hbond:

	ase				PHE:237;PHE:237;GLY:240;GLU:248;<br>ASN:265;ARG:267;ALA:345<br>Hydrophobic: PHE:237;LEU:252;<br>GLU:270;TRP:331;<br>LEU:344;ARG:352<br>Pi-stacking: TYR:291<br>Salt bridge: LYS:283;ARG:352;<br>ARG:352<br>Pi-cation: ARG:352
Miltirone	Glucosidase	7KBJ	-7.9	0.0	Hbond: TYR:291<br>Hydrophobic:<br>LEU:252;LEU:344;PHE:350<br>Pi-cation: ARG:352
Myristicin	Glucosidase	7KBJ	-5.5	1.9	Hydrophobic:<br>PHE:237;LEU:252;LEU:252
Phytol	Glucosidase	7KBJ	-5.5	4.3	Hydrophobic:<br>PHE:237;TYR:246;LEU:252;<br>GLU:270;TYR:291;LEU:344
Piperitenone oxide	Glucosidase	7KBJ	-6.0	0.1	Hbond: ARG:334;ALA:345<br>Hydrophobic: ILE:327;TRP:331;<br>TYR:348
Przewalskinic acid A	Glucosidase	7KBJ	-7.8	0.5	Hbond:<br>MET:289;TYR:291;ARG:334;<br>ALA:345<br>Hydrophobic: TYR:291;LEU:344<br>Salt bridge: ARG:352
Rosmarinic acid	Glucosidase	7KBJ	-8.5	1.0	Hbond: ASP:251;LEU:252;GLY:253;<br>GLY:254;LYS:283;TYR:348<br>Hydrophobic: LEU:252;TYR:291;<br>TRP:331;LEU:344<br>Salt bridge: ARG:352
Sagerinic acid	Glucosidase	7KBJ	-6.0	0.0	NA
Sahandinone	Glucosidase	7KBJ	-7.2	0.1	Hbond: TYR:291<br>Hydrophobic:

					ILE:327;PHE:330;TYR:348;<br>PHE:350<br>Pi-cation: ARG:352
Salvianolic acid K	Glucosidase	7KBJ	-7.8	3.1	Hbond:<br>GLY:264;MET:289;TYR:291;TYR:291;<br>ALA:345;TYR:348;PHE:350;ARG:352<br>Hydrophobic:<br>LEU:252;LEU:252;TRP:331;LEU:344<br>Salt bridge: LYS:283;ARG:352<br>Pi-cation: ARG:334
Taxodione	Glucosidase	7KBJ	-8.1	0.7	Hbond: PHE:350;ARG:352<br>Hydrophobic:<br>TYR:291;TYR:291;TRP:331;<br>PHE:350;ARG:352
Viridone	Glucosidase	7KBJ	-8.2	1.1	Hbond: ARG:352<br>Hydrophobic:<br>LEU:252;TYR:291;PHE:350
Yunnaneic acid D	Glucosidase	7KBJ	-6.9	8.7	Hbond: GLY:264;TYR:291;<br>TYR:348;ARG:352<br>Hydrophobic: LEU:252;TYR:291;<br>TRP:331;LEU:344<br>Salt bridge: ARG:352
3,5-dicaffeoylquinic acid	HCAI	3LXE	-8.8	0.8	Hbond: SER:65<br>PHE:66;GLN:92;HIS:94;HIS:96;<br>HIS:119;THR:199;HIS:200<br>Hydrophobic:<br>VAL:143;VAL:143;LEU:198;<br>HIS:200;TRP:209<br>Pi-stacking: HIS:64;HIS:94;HIS:200<br>Pi-cation: HIS:94
4,5-dicaffeoylquinic acid	HCAI	3LXE	-8.1	0.4	Hbond: ASN:69;GLN:92;HIS:96;<br>HIS:119;THR:199<br>Hydrophobic:

					LEU:131;LEU:198;HIS:200<br>Pi-stacking: HIS:94<br>Salt bridge: HIS:67<br>Pi-cation: HIS:64
6-Hydroxy-5,6-dehydrosugiol	HCAI	3LXE	-8.2	0.9	Hbond: HIS:64;HIS:94;HIS:96<br>Hydrophobic: VAL:62;HIS:119;<br>VAL:143;LEU:198;VAL:207;TRP:209
8,13-Epoxy-15,16-dinorlabd-12-ene	HCAI	3LXE	-7.7	0.0	Hydrophobic:<br>PHE:91;LEU:131;ALA:135;<br>LEU:141;LEU:198;PRO:202;TRP:209
Caffeic acid	HCAI	3LXE	-6.4	0.7	Hbond:<br>TRP:5;TYR:7;ASN:11;HIS:64;<br>GLN:242;HIS:243<br>Hydrophobic: TYR:7;HIS:64<br>Salt bridge: LYS:170
Danshensu	HCAI	3LXE	-6.5	1.1	Hbond: TRP:5;ASN:11;SER:231;<br>GLN:242;HIS:243<br>Hydrophobic: TYR:7;TYR:7;HIS:64<br>Salt bridge: HIS:64;LYS:170
Docosane	HCAI	3LXE	-5.2	5.3	Hydrophobic:<br>PHE:91;ALA:121;LEU:131;<br>ALA:135;LEU:141;VAL:143;<br>LEU:198;HIS:200
Grandifloroside	HCAI	3LXE	-7.2	1.1	Hbond: SER:65;SER:65;PHE:66;<br>THR:199<br>Hydrophobic: LEU:198;HIS:200<br>Salt bridge:<br>HIS:64;HIS:64;HIS:67;HIS:67;<br>HIS:94; HIS:96;HIS:119;HIS:200<br>Pi-cation: HIS:64;HIS:67;HIS:200
Hexahydrofarnesylacetone	HCAI	3LXE	-5.1	0.0	Hbond: TYR:7;HIS:64<br>Hydrophobic: ALA:121;LEU:131;<br>LEU:131;ALA:132;ALA:135;LEU:198;

					LEU:198;HIS:200;PRO:202;TYR:20<br>4
Lamiidoside	HCAI	3LXE	-8.3	0.2	Hbond: HIS:64;HIS:67;PHE:70;<br>ASP:72;HIS:94;HIS:96;THR:199<br>Hydrophobic: PHE:91<br>Salt bridge: HIS:67;HIS:200
Martynoside	HCAI	3LXE	-8.1	0.4	Hbond: LYS:57;PHE:70;GLN:92;<br>HIS:96;THR:199<br>Hydrophobic:<br>TRP:5;VAL:62;VAL:62;PHE:91;<br>LEU:131;VAL:143;LEU:198<br>Salt bridge: HIS:64;HIS:94;HIS:200<br>Pi-cation: HIS:94
Miltirone	HCAI	3LXE	-8.4	0.0	Hydrophobic: PHE:91;HIS:119;<br>ALA:121;VAL:143;VAL:143;<br>LEU:198;VAL:207;TRP:209;TRP:20<br>9<br>Pi-stacking: HIS:94
Myristicin	HCAI	3LXE	-6.2	0.0	Hbond: HIS:94;HIS:96;HIS:119;<br>THR:199<br>Hydrophobic: LEU:198;HIS:200
Phytol	HCAI	3LXE	-5.2	5.0	Hbond: TYR:7<br>Hydrophobic: TRP:5;TRP:5;<br>LEU;TYR:20;HIS:64
Piperitenone oxide	HCAI	3LXE	-6.4	0.1	Hbond: THR:199;HIS:200<br>Hydrophobic: ALA:121;VAL:143;<br>LEU:198;TRP:209
Przewalskinic acid A	HCAI	3LXE	-7.8	0.1	Hbond: TRP:5;HIS:64;THR:199;<br>PRO:201<br>Hydrophobic:<br>TRP:5;VAL:62;LEU:198<br>Pi-stacking: HIS:94<br>Pi-cation: HIS:200
Rosmarinic acid	HCAI	3LXE	-8.3	0.8	Hbond: SER:65;SER:65;PHE:66;<br>HIS:96;THR:199;HIS:200<br>Hydrophobic:

					PHE:91;VAL:143;VAL:143; LEU:198;HIS:200;TRP:209<br>Pi-stacking: HIS:64;HIS:94;HIS:94
Sagerinic acid	HCAI	3LXE	-6.4	0.0	Hbond: HIS:64;GLN:92;HIS:94; HIS:96<br>Pi-cation: HIS:94;HIS:94
Sahandinone	HCAI	3LXE	-7.6	0.0	Hbond: GLN:92;THR:199;HIS:200<br>Hydrophobic: PHE:91;HIS:119; LEU:131;VAL:143;LEU:198;HIS:200; VAL:207;TRP:209<br>Pi-stacking: HIS:94
Salvianolic acid K	HCAI	3LXE	-8.3	1.0	Hbond: HIS:64;HIS:67;ASN:69; ASP:72;GLN:92;THR:199;THR:199<br>Hydrophobic: PHE:91;PHE:91; LEU:131;LEU:198;HIS:200<br>Pi-stacking: HIS:94<br>Salt bridge: HIS:67;HIS:94;HIS:96;HIS:119
Taxodione	HCAI	3LXE	-8.3	1.1	Hbond: THR:199;HIS:200<br>Hydrophobic: VAL:143;LEU:198;TRP:209
Viridone	HCAI	3LXE	-8.0	1.0	Hbond: HIS:94;HIS:96;HIS:119<br>Hydrophobic: HIS:119;VAL:143;LEU:198; HIS:200;VAL:207;TRP:209
Yunnaneic acid D	HCAI	3LXE	-8.5	1.1	Hbond: HIS:64;HIS:67;THR:199<br>Hydrophobic: PHE:91;LEU:131; LEU:198<br>Pi-stacking: HIS:200<br>Salt bridge: HIS:94;HIS:94;HIS:96;HIS:119
3,5-dicaffeoylquinic acid	hCAII	4IWZ	-9.1	1.1	Hbond: TYR:7;ASN:67;GLU:69; GLN:92;GLU:106;THR:199;THR:200<br>Hydrophobic:

					HIS:64;ILE:91;GLN:92;PHE:131<br>Pi-stacking: PHE:131<br>Salt bridge: HIS:94;HIS:96;HIS:119
4,5-dicaffeoylquinic acid	hCAII	4IWZ	-8.4	0.9	Hbond: ARG:58;GLN:92;GLU:106;<br>THR:199;THR:200<br>Hydrophobic: TRP:5;LEU:198<br>Salt bridge: HIS:94;HIS:96;HIS:119
6-Hydroxy-5,6-dehydrosugiol	hCAII	4IWZ	-7.8	0.4	Hbond: GLN:92;HIS:94;HIS:96;<br>HIS:119;THR:199<br>Hydrophobic:<br>TRP:5;HIS:119;VAL:121;<br>VAL:143;<br>TRP:209;TRP:209<br>Pi-cation: HIS:94
8,13-Epoxy-15,16-dinorlabd-12-ene	hCAII	4IWZ	-7.0	0.0	Hydrophobic:<br>HIS:119;VAL:121;PHE:131;<br>VAL:143; LEU:198;TRP:209
Caffeic acid	hCAII	4IWZ	-6.4	1.0	Hbond:<br>GLN:92;HIS:119;THR:199;THR:200<br>Hydrophobic:<br>VAL:121;VAL:143;LEU:198<br>Pi-stacking: HIS:94
Danshensu	hCAII	4IWZ	-6.2	1.1	Hbond:<br>TYR:7;TYR:7;GLY:63;LYS:170<br>Hydrophobic: TYR:7;PHE:231<br>Pi-stacking: PHE:231<br>Salt bridge: HIS:64;LYS:170
Docosane	hCAII	4IWZ	-4.8	7.7	Hydrophobic:<br>TRP:5;TRP:5;HIS:64;VAL:121;<br>PHE:131;VAL:143;LEU:198;THR:20<br>0;<br>TRP:209
Grandifloroside	hCAII	4IWZ	-8.3	0.3	Hbond:<br>HIS:4;HIS:4;TRP:5;ASN:62;ASN:62<br>;<br>HIS:64;GLN:92;THR:199;THR:199;

					THR:200;PRO:201<br>Hydrophobic: VAL:121;PHE:131;<br>VAL:143;LEU:198<br>Pi-stacking: HIS:94<br>Salt bridge: HIS:64;HIS:64;HIS:94
Hexahydrofarnesylacetone	hCAII	4IWZ	-5.2	0.0	Hbond: THR:199<br>Hydrophobic: LEU:57;GLU:69;<br>ILE:91HIS:119;PHE:131;VAL:143;<br>LEU:198;TRP:209
Lamiidoside	hCAII	4IWZ	-8.8	0.4	Hbond:<br>HIS:4;TRP:5;ASN:62;HIS:64;ASN:67<br>Hydrophobic: PHE:131;PHE:131;<br>VAL:135;LEU:198;<br>THR:200;PRO:202;LEU:204<br>Salt bridge:<br>HIS:64;HIS:64;HIS:94;HIS:94;HIS:96
Martynoside	hCAII	4IWZ	-7.8	0.8	Hbond:<br>HIS:4;HIS:4;HIS:64;ASN:67;LYS:170;<br>THR:200;THR:200<br>Hydrophobic: PHE:131;LYS:170<br>Salt bridge: HIS:64;HIS:64<br>Pi-cation: LYS:170
Miltirone	hCAII	4IWZ	-8.4	0.0	Hydrophobic:<br>HIS:119;VAL:121;PHE:131;<br>VAL:143;LEU:198;THR:200;TRP:209<br>Pi-cation: HIS:94
Myristicin	hCAII	4IWZ	-5.8	2.9	Hbond: HIS:94;HIS:96;HIS:119;<br>THR:199<br>Hydrophobic: PHE:131;LEU:19
Phytol	hCAII	4IWZ	-5.8	0.6	Hbond: TYR:7;TYR:7;HIS:96<br>Hydrophobic:<br>TRP:5;HIS:64;ALA:65;LYS:170;

					THR:200;PHE:231
Piperitenone oxide	hCAII	4IWZ	-6.5	0.0	Hbond: THR:199;THR:200<br>Hydrophobic:<br>HIS:119;PHE:131;VAL:143;<br>LEU:198;THR:199;TRP:209
Przewalskinic acid A	hCAII	4IWZ	-7.4	0.5	Hbond: GLN:92;HIS:94;HIS:96;<br>HIS:119;THR:199<br>Hydrophobic: LEU:198;THR:200<br>Salt bridge: HIS:64
Rosmarinic acid	hCAII	4IWZ	-8.0	1.7	Hbond: ASN:62;ASN:67;GLN:92;<br>THR:199;THR:200<br>Hydrophobic:<br>TRP:5;HIS:64;VAL:121;VAL:143;<br>LEU:198;THR:200<br>Pi-stacking: HIS:94
Sagerinic acid	hCAII	4IWZ	-5.9	0.0	Hbond: GLN:92;HIS:94;HIS:96<br>Pi-cation: HIS:94;HIS:94
Sahandinone	hCAII	4IWZ	-7.6	0.0	Hbond: GLN:92;THR:199;THR:200<br>Hydrophobic:<br>ILE:91;VAL:121;PHE:131;VAL:143;<br>LEU:198;LEU:198;TRP:209
Salvianolic acid K	hCAII	4IWZ	-8.4	2.4	Hbond: ASN:62;ASN:67;PHE:70;<br>ASP:72;GLN:92;HIS:119;PHE:131;<br>GLY:132;THR:199;THR:200<br>Hydrophobic:<br>GLU:69;ILE:91;GLN:92;VAL:121;<br>PHE:131;LEU:198<br>Pi-stacking: HIS:94<br>Salt bridge: HIS:94
Taxodione	hCAII	4IWZ	-8.2	0.4	Hbond: GLN:92;THR:199;THR:200<br>Hydrophobic:<br>VAL:143;LEU:198;TRP:209
Viridone	hCAII	4IWZ	-8.1	0.3	Hbond:<br>GLN:92;THR:199;THR:200;PRO:20<br>1<br>Hydrophobic:

					PHE:131;VAL:143;LEU:198;TRP:209
Yunnaneic acid D	hCAII	4IWZ	-8.3	1.0	Hbond: TRP:5;ASN:62;ASN:67; GLN:92;THR:199;;THR:200<br>Hydrophobic: VAL:121;PHE:131;LEU:198<br>Pi-stacking: HIS:94<br>Salt bridge: HIS:4;HIS:64;HIS:94
3,5-dicaffeoylquinic acid	Tyrosinase	6QXD	-6.8	1.7	Hbond: HIS:208;ARG:209;MET:215<br>Hydrophobic: ASN:205;VAL:218;VAL:218;VAL:218<br>Pi-stacking: HIS:208<br>Salt bridge: ARG:209
4,5-dicaffeoylquinic acid	Tyrosinase	6QXD	-7.0	0.4	Hbond: HIS:42;ARG:209<br>Hydrophobic: VAL:218;VAL:218;VAL:218<br>Pi-stacking: HIS:208<br>Salt bridge: ARG:209
6-Hydroxy-5,6-dehydrosugiol	Tyrosinase	6QXD	-6.0	0.1	Hbond: ASN:205<br>Hydrophobic: ASN:205;ARG:209;VAL:218; ALA:221
8,13-Epoxy-15,16-dinorlabd-12-ene	Tyrosinase	6QXD	-7.7	0.0	Hydrophobic: PHE:197;ASN:205;HIS:208; VAL:218;VAL:218
Caffeic acid	Tyrosinase	6QXD	-6.2	1.0	Hbond: ASN:205;VAL:218<br>Hydrophobic: HIS:208;VAL:218<br>Pi-stacking: HIS:208<br>Salt bridge: ARG:209
Danshensu	Tyrosinase	6QXD	-6.3	0.4	Hbond: ARG:209;VAL:217; VAL:218<br>Hydrophobic: HIS:208;VAL:218<br>Pi-stacking: HIS:208<br>Salt bridge: ARG:209
Docosane	Tyrosinas	6QXD	-4.1	2.8	Hydrophobic: HIS:60;PHE:197;

	Tyrosinase				PHE:197; ASN:205;HIS:208;VAL:218
Grandifloroside	Tyrosinase	6QXD	-5.4	4.8	Hbond: ASN:57;ASN:205;GLY:216<br>Hydrophobic: ASN:205;VAL:218<br>Pi-stacking: HIS:208<br>Salt bridge: ARG:209
Lamiidoside	Tyrosinase	6QXD	-6.0	1.1	Hbond: GLY:46;ASN:57;HIS:60; VAL:218<br>Hydrophobic: HIS:208;VAL:218;ALA:221<br>Pi-stacking: HIS:208<br>Salt bridge: ARG:209
Martynoside	Tyrosinase	6QXD	-5.0	0.7	Hbond: GLY:46<br>Hydrophobic: VAL:217;VAL:218
Miltirone	Tyrosinase	6QXD	-6.9	0.1	Hydrophobic: PHE:197;VAL:218; ALA:221;PHE:227<br>Pi-stacking: HIS:208
Myristicin	Tyrosinase	6QXD	-5.7	0.0	Hydrophobic: VAL:218<br>Pi-stacking: HIS:208;HIS:208
Phytol	Tyrosinase	6QXD	-4.0	4.4	Hbond: HIS:42<br>Hydrophobic: PHE:197;ASN:205; HIS:208;VAL:218;VAL:218; PRO:219
Piperitenone oxide	Tyrosinase	6QXD	-5.7	0.0	Hydrophobic: PHE:197;VAL:218;VAL:218;ALA:221
Przewalskinic acid A	Tyrosinase	6QXD	-5.0	0.5	Hbond: ARG:209;MET:215<br>Hydrophobic: VAL:218
Rosmarinic acid	Tyrosinase	6QXD	-6.6	0.3	Hbond: HIS:42;ASN:205;ARG:209;MET:215<br>Hydrophobic: VAL:217;VAL:218;VAL:218<br>Pi-stacking: HIS:208<br>Salt bridge: ARG:209;ARG:209
Sagerinic acid	Tyrosinase	6QXD	-5.7	0.0	Pi-stacking: HIS:208

Sahandinone	Tyrosinase	6QXD	-4.3	8.3	Hbond: VAL:218<br>Hydrophobic: PHE:197;PHE:197;VAL:218; ALA:221
Salvianolic acid K	Tyrosinase	6QXD	-5.9	7.5	Hbond: GLY:46;MET:61;GLU:195; ASN:205;GLY:216<br>Hydrophobic: HIS:208;VAL:218;VAL:218;VAL:218<br>Pi-stacking: HIS:208<br>Salt bridge: HIS:60;HIS:204
Taxodione	Tyrosinase	6QXD	-6.1	0.9	Hydrophobic: HIS:60;PHE:197; VAL:218
Viridone	Tyrosinase	6QXD	-5.7	0.9	Hbond: ARG:209<br>Hydrophobic: HIS:60;VAL:218
Yunnaneic acid D	Tyrosinase	6QXD	-7.0	7.8	Hbond: HIS:42;GLY:46;GLY:46; ASN:205;ARG:209<br>Hydrophobic: VAL:218;VAL:218;VAL:218<br>Pi-stacking: HIS:208

Compound	Target	PDB ID	Binding energy (kcal/mol)	RMSD (Å)	Interaction type and binding site residues
3,5-dicaffeoylquinic acid	EGFR	1m17	-8.8	0.1	Hbond: LYS:721;MET:769;ARG:817<br>Hydrophobic: LEU:694;PHE:699;PHE:699;VAL:702;ALA:719;LEU:820<br>Salt bridge: LYS:721
4,5-dicaffeoylquinic acid	EGFR	1m17	-9.0	1.0	Hbond: ALA:719;LYS:721;THR:766;THR:766;MET:769;MET:769;ARG:817<br>Hydrophobic: LEU:694;VAL:702;VAL:702;VAL:702;LYS:721;LYS:721;ARG:817;LEU:820<br>Salt bridge: LYS:721
6-Hydroxy-5,6-dehydrosugiol	EGFR	1m17	-8.9	0.8	Hbond: LYS:721<br>Hydrophobic: LEU:694;PHE:699;VAL:702;VAL:702;LYS:721;LYS:721;LEU:820
8,13-Epoxy-15,16-dinorlabd-12-ene	EGFR	1m17	-8.2	0.0	Hydrophobic: LEU:694;VAL:702;VAL:702;ALA:719;LYS:721;LYS:721;LEU:764;THR:766;LEU:820;THR:830
Caffeic acid	EGFR	1m17	-6.3	0.9	Hbond: MET:769;THR:830;ASP:831;ASP:831<br>Hydrophobic: LEU:694;VAL:702;ALA:719;LEU:820<br>Salt bridge: LYS:721
Danshensu	EGFR	1m17	-6.3	0.8	Hbond: LYS:721;GLU:738;GLN:767;ME

					T:769;ASP:831;ASP:831;ASP:831 Hydrophobic: LYS:721
Docosane	EGFR	1m17	-5.3	4.3	Hydrophobic: PHE:699;PHE:699;PHE:699;VAL:702;VAL:702;VAL:702;ALA:719;LYS:721;LEU:764;THR:766;LEU:820
Grandifloroside	EGFR	1m17	-8.3	0.9	Hbond: LYS:721;LYS:721;GLU:738;THR:766;THR:766;MET:769;GLY:772;ARG:817;ASP:831;ASP:831;ASP:831 Hydrophobic: VAL:702;VAL:702 Salt bridge: LYS:721
Hexahydrofarnesylacetone	EGFR	1m17	-5.5	0.0	Hydrophobic: PHE:699;PHE:699;PHE:699;PHE:699;VAL:702;VAL:702;ALA:719;LEU:723;GLU:734;ILE:735;LEU:820
Lamiidoside	EGFR	1m17	-9.2	1.0	Hbond: MET:769;GLY:772;ASP:831;ASP:831 Hydrophobic: PHE:699;PHE:699;PHE:699;VAL:702;LEU:820 Salt bridge: LYS:721
Martynoside	EGFR	1m17	-8.9	1.0	Hbond: GLU:738;GLU:738;MET:769;CYS:773;ASP:831;ASP:831 Hydrophobic: LEU:694;LEU:694;PHE:699;PHE:699;LYS:721 Pi-stacking: PHE:699 Pi-cation: LYS:721
Miltirone	EGFR	1m17	-8.9	0.0	Hydrophobic: PHE:699;VAL:702;VAL:702;ASP

					:831
Myristicin	EGFR	1m17	-6.2	0.0	Hbond: LYS:721;GLU:738<br>Hydrophobic: LEU:694;LEU:820
Phytol	EGFR	1m17	-6.1	0.8	Hbond:<br>ALA:719;LYS:721;THR:766<br>Hydrophobic:<br>PHE:699;PHE:699;PHE:699;PHE:699;VAL:702;VAL:702;ALA:719;ALA:731;LEU:820
Piperitenone oxide	EGFR	1m17	-6.4	0.0	Hbond: ASP:831;ASP:831<br>Hydrophobic:<br>LEU:764;THR:766;LEU:820;THR:830
Przewalskinic acid A	EGFR	1m17	-8.5	0.4	Hbond:<br>GLU:738;MET:769;ASP:831;ASP:831<br>Hydrophobic:<br>LYS:721;LEU:820;LEU:820<br>Pi-cation: LYS:721
Rosmarinic acid	EGFR	1m17	-8.2	0.4	Hbond:<br>LYS:721;THR:766;GLN:767;MET:769;ASN:818;ASP:831;ASP:831;ASP:831;ASP:831<br>Hydrophobic:<br>PHE:699;VAL:702;VAL:702;ALA:719;LEU:820
Sagerinic acid	EGFR	1m17	-6.2	0.0	Hbond: LYS:721
Sahandinone	EGFR	1m17	-8.2	0.1	Hydrophobic:<br>LEU:694;PHE:699;PHE:699;VAL:702;VAL:702;ALA:719;LEU:820
Salvianolic acid K	EGFR	1m17	-9.0	0.3	Hbond:<br>LYS:721;GLU:738;GLU:738;ASP:813;ASP:831;ASP:831;ASP:831;ASP:831<br>Hydrophobic:

					PHE:699;VAL:702;LYS:721;GLU:734<br>Pi-stacking: PHE:699
Taxodione	EGFR	1m17	-8.5	0.7	Hbond: LYS:721<br>Hydrophobic: PHE:699;VAL:702;VAL:702;LYS:721;LEU:820
Viridone	EGFR	1m17	-8.4	1.0	Hbond: LYS:721<br>Hydrophobic: LEU:694;PHE:699;VAL:702;LYS:721;THR:766;LEU:820
Yunnaneic acid D	EGFR	1m17	-9.3	1.0	Hbond: MET:769;ARG:817;ASN:818;ASN:818;ASP:831;ASP:831<br>Hydrophobic: PHE:699;VAL:702;VAL:702;ALA:719;LYS:721;MET:769;LEU:820<br>Salt bridge: LYS:721;LYS:721
3,5-dicaffeoylquinic acid	EP300	7vhy	-11.0	12.2	Hbond: ASP:1399;SER:1400;TRP:1436;TRP:1436;ASP:1445;ASP:1445;GLN:1455<br>Hydrophobic: LEU:1398;LEU:1398;ILE:1435;TYR:1446;ILE:1457;PHE:1467<br>Pi-stacking: HIS:1451
4,5-dicaffeoylquinic acid	EP300	7vhy	-9.3	16.2	Hbond: SER:1171;THR:1174;THR:1174;ASP:1399;ASP:1399;GLY:1443;TYR:1446;HIS:1451;HIS:1451;HIS:1451;SER:1642<br>Hydrophobic: PRO:1440;HIS:1451;PRO:1453;PRO:1453;ILE:1457;ILE:1457<br>Pi-stacking: HIS:1451

6-Hydroxy-5,6-dehydrosugiol	EP300	7vhy	-7.2	0.6	Hbond: ASP:1330;ARG:1356;GLY:1382 Hydrophobic: ASP:1330;PRO:1354;ARG:1356
8,13-Epoxy-15,16-dinorlabd-12-ene	EP300	7vhy	-8.6	4.6	Hydrophobic: LEU:1398;LEU:1398;PRO:1440;PRO:1458;TRP:1466
Caffeic acid	EP300	7vhy	-6.8	14.5	Hbond: TYR:1394;TYR:1397;LEU:1398;TRP:1436;CYS:1438;GLY:1506 Hydrophobic: TYR:1397;TRP:1436;TRP:1436;TYR:1446
Danshensu	EP300	7vhy	-6.8	1.1	Hbond: TYR:1394;SER:1396;TYR:1397;TYR:1397;LEU:1398;CYS:1438;TYR:1446;TYR:1446 Hydrophobic: TYR:1397;TRP:1436;TRP:1436
Docosane	EP300	7vhy	-5.8	5.1	Hydrophobic: PHE:1374;HIS:1402;TYR:1414;TYR:1414;TYR:1414;LEU:1418;PRO:1458;LEU:1463;LEU:1463;TRP:1466;TRP:1466;TRP:1466;TRP:1466;PHE:1467
Grandifloroside	EP300	7vhy	-8.2	7.6	Hbond: PRO:1172;THR:1174;THR:1174;THR:1174;HIS:1451 Hydrophobic: GLU:1442;HIS:1451;ILE:1457;ILE:1457
Hexahydrofarnesylacetone	EP300	7vhy	-6.3	8.3	Hbond: ASP:1445 Hydrophobic: ILE:1395;LEU:1398;LEU:1398;TYR:1446;HIS:1451;GLN:1455;LE

					U:1463;TRP:1466
Lamiidoside	EP300	7vhy	-8.9	4.6	Hbond: SER:1171;ARG:1187;GLY:1443; ASP:1444;HIS:1451;GLN:1455;SER:1642 Hydrophobic: PRO:1440;ILE:1457 Salt bridge: HIS:1451
Martynoside	EP300	7vhy	-10.3	1.0	Hbond: ILE:1395;SER:1400;ARG:1410;TYR:1414;TRP:1436;ASP:1445;GLN:1455 Hydrophobic: LEU:1398;LEU:1398;PRO:1440;TYR:1446;HIS:1451;GLN:1455;ILE:1457;PHE:1467 Pi-stacking: HIS:1451
Miltirone	EP300	7vhy	-9.4	0.0	Hydrophobic: LEU:1398;TYR:1414;TYR:1446;PRO:1458;LEU:1463;LEU:1463;PHE:1467;PHE:1467 Pi-stacking: TRP:1466
Myristicin	EP300	7vhy	-6.4	7.7	Hbond: ASP:1399;SER:1400;GLN:1455 Hydrophobic: LEU:1398;PHE:1467
Phytol	EP300	7vhy	-5.4	12.3	Hydrophobic: TYR:1397;TRP:1436;TRP:1436;TRP:1436;TYR:1446;PHE:1508;HIS:1591;PHE:1595
Piperitenone oxide	EP300	7vhy	-6.7	0.0	Hydrophobic: PHE:1374;PHE:1374;LEU:1398;TYR:1414;LEU:1418;LEU:1463;LEU:1463;TRP:1466
Przewalskinic acid A	EP300	7vhy	-9.0	9.7	Hbond: ASP:1399;ASP:1399;SER:1400;ASP:1445;TYR:1446;GLN:1455

Rosmarinic acid	EP300	7vhy	-9.2	17.6	Hydrophobic: PRO:1440;HIS:1451;GLN:1455;ILE:1457<br>Pi-stacking: HIS:1451<br>Salt bridge: HIS:1451<br>Hbond: TYR:1394;ASP:1399;TRP:1436;TRP:1436<br>Hydrophobic: LEU:1398;LEU:1398;TYR:1414;TYR:1446;LEU:1463;TRP:1466<br>Pi-stacking: TRP:1466;TRP:1466
Sagerinic acid	EP300	7vhy	-5.7	0.3	Hbond: GLN:1455
Sahandinone	EP300	7vhy	-8.2	7.2	Hbond: GLN:1455<br>Hydrophobic: PRO:1440;TYR:1446;TYR:1446;HIS:1451;ILE:1457;PRO:1458
Salvianolic acid K	EP300	7vhy	-9.7	4.9	Hbond: TRP:1436;TRP:1436;GLU:1442;GLY:1443;ASP:1444;ASP:1444;ASP:1445;ASP:1445;PRO:1453<br>Hydrophobic: LEU:1398;LEU:1398;PRO:1440;TYR:1446;ILE:1457;ILE:1457;PHE:1467<br>Pi-stacking: HIS:1451
Taxodione	EP300	7vhy	-7.2	35.8	Hbond: SER:1214<br>Hydrophobic: PHE:1200;ILE:1208;ILE:1208;GLN:1209;VAL:1213
Viridone	EP300	7vhy	-7.4	40.4	Hbond: SER:1214;SER:1214<br>Hydrophobic: PHE:1200;ILE:1208;ILE:1208;GLN:1209;VAL:1213
Yunnaneic	EP300	7vhy	-8.8	2.0	Hbond:

acid D					SER:1171;THR:1174;THR:1174;ASP:1399;GLY:1443;ASP:1445;GLN:1455<br>Hydrophobic: GLN:1173;PRO:1440;HIS:1451;PRO:1453;GLN:1455;ILE:1457<br>Pi-stacking: HIS:1451
3,5-dicaffeoylquinic acid	ESR1	4ZNH	-8.3	0.7	Hbond: LEU:306;ALA:307;ALA:307;ASN:359;ASN:359;LYS:362;ARG:363<br>Hydrophobic: LYS:362<br>Salt bridge: ARG:363
4,5-dicaffeoylquinic acid	ESR1	4ZNH	-8.0	1.1	Hbond: ALA:307;ARG:363;ARG:363;ARG:363;ARG:363;ARG:363;GLY:366;ASP:369;ASP:369<br>Hydrophobic: ALA:318;LYS:362;ARG:363;ARG:363;VAL:368
6-Hydroxy-5,6-dehydrosugiol	ESR1	4ZNH	-7.8	0.9	Hbond: ARG:363;ARG:363;ARG:363<br>Hydrophobic: ALA:318;LYS:362;ARG:363;ARG:363
8,13-Epoxy-15,16-dinorlabd-12-ene	ESR1	4ZNH	-8.5	6.0	Hydrophobic: LEU:346;LEU:346;ALA:350;TRP:383;LEU:384;LEU:387;LEU:391;PHE:404;PHE:425;LEU:536;LEU:540
Caffeic acid	ESR1	4ZNH	-6.2	1.0	Hbond: MET:343;THR:347;THR:347<br>Hydrophobic: LEU:346;ALA:350;TRP:383;TRP:383;LEU:384;LEU:525;LEU:540
Danshensu	ESR1	4ZNH	-6.1	1.1	Hbond:

					MET:343;THR:347;THR:347 Hydrophobic: LEU:346;ALA:350;TRP:383;TRP:383;LEU:384;LEU:525
Docosane	ESR1	4ZNH	-4.0	7.1	Hydrophobic: LYS:362;ARG:363;ARG:363;ARG:363;ARG:363;PRO:365;VAL:368
Grandifloroside	ESR1	4ZNH	-7.3	1.1	Hbond: GLU:385;ASN:455;ARG:515;HIS:516;ASN:519 Hydrophobic: LEU:508;LEU:508;LEU:511;LEU:511 Salt bridge: ARG:515
Hexahydrofarnesylacetone	ESR1	4ZNH	-4.9	7.4	Hydrophobic: LEU:320;PRO:324;ILE:326;ILE:386;LEU:387;TRP:393;ARG:394;LYS:449
Lamiidoside	ESR1	4ZNH	-7.8	1.0	Hbond: GLU:323;GLU:323;GLU:323;GLU:323;GLU:353 Hydrophobic: PRO:324;ILE:326;GLU:353
Martynoside	ESR1	4ZNH	-7.9	0.7	Hbond: LEU:320;LEU:320;GLU:323;GLU:353;ILE:386;GLY:390;GLY:442 Hydrophobic: LEU:320;PRO:324;PRO:325;TRP:393 Pi-cation: ARG:394
Miltirone	ESR1	4ZNH	-7.3	26.8	Hydrophobic: GLN:441;GLU:444;LEU:489;ALA:493
Myristicin	ESR1	4ZNH	-5.9	0.1	Hbond: THR:347 Hydrophobic:

					LEU:346;ALA:350;LEU:391;PHE:404;PHE:404
Phytol	ESR1	4ZNH	-5.4	17.9	Hbond: THR:483<br>Hydrophobic: ILE:451;ILE:452;LEU:508;LEU:511;LEU:511;SER:512;ARG:515;ARG:515;HIS:516
Piperitenone oxide	ESR1	4ZNH	-6.4	0.1	Hydrophobic: ALA:350;LEU:387;PHE:404;PHE:404
Przewalskinic acid A	ESR1	4ZNH	-8.0	34.2	Hbond: THR:347;GLY:420;GLY:420<br>Hydrophobic: LEU:346;ALA:350;PHE:404;VAL:418;MET:421;LEU:525
Rosmarinic acid	ESR1	4ZNH	-6.9	4.8	Hbond: LEU:306;LEU:306;LEU:306;ALA:307;ASP:369<br>Hydrophobic: LEU:310;ALA:318;PRO:365;VAL:368<br>Salt bridge: ARG:363
Sagerinic acid	ESR1	4ZNH	-5.6	3.7	
Sahandinone	ESR1	4ZNH	-6.7	0.1	Hydrophobic: LYS:362;ARG:363;ARG:363;ARG:363;VAL:368
Salvianolic acid K	ESR1	4ZNH	-7.1	22.8	Hbond: ASN:455;LEU:508;SER:512;SER:512;ARG:515<br>Hydrophobic: LEU:511;SER:512;ARG:515;ARG:515<br>Salt bridge: ARG:515;ARG:515
Taxodione	ESR1	4ZNH	-8.6	0.6	Hbond: LYS:362;ARG:363;ARG:363

Viridone	ESR1	4ZNH	-7.9	1.0	Hydrophobic: LYS:362;ARG:363;ARG:363;ARG:363;VAL:368<br>Hbond: ARG:363;ARG:363<br>Hydrophobic: ALA:318;LYS:362;ARG:363;VAL:368
Yunnaneic acid D	ESR1	4ZNH	-7.5	0.9	Hbond: ALA:307;ASP:321;ARG:363;ARG:363;GLY:366;ASP:369<br>Hydrophobic: LEU:310;ALA:318;ARG:363;ARG:363;VAL:368
3,5-dicaffeoylquinic acid	HRAS	1p2s	-10.3	0.9	Hbond: GLY:13;GLY:15;SER:17;ALA:18;GLU:31;THR:35;THR:35;ASP:38;TYR:40;LYS:117;ALA:146;LYS:147<br>Hydrophobic: ALA:18;ILE:21;ILE:21;VAL:29;VAL:29;ASP:33;LYS:117;ALA:146<br>Pi-stacking: PHE:28<br>Salt bridge: LYS:16;LYS:117
4,5-dicaffeoylquinic acid	HRAS	1p2s	-9.9	0.8	Hbond: GLY:13;VAL:14;GLY:15;LYS:16;SER:17;ALA:18;ASP:33;THR:35;ASP:119;ALA:146;LYS:147<br>Hydrophobic: ALA:18;GLN:61;ALA:146<br>Pi-stacking: PHE:28<br>Salt bridge: LYS:16;LYS:117
6-Hydroxy-5,6-dehydrosugiol	HRAS	1p2s	-8.1	0.3	Hbond: GLY:13;GLY:15;LYS:16;LYS:16;SER:17;GLU:31;ASP:33
8,13-Epoxy-	HRAS	1p2s	-6.9	0.0	Hydrophobic:

15,16-dinorlabd-12-ene					ALA:18;PHE:28;PHE:28;LYS:117
Caffeic acid	HRAS	1p2s	-6.6	0.6	Hbond: LYS:117;ASP:119;ASP:119;ALA:146;LYS:147<br>Hydrophobic: ALA:18;ALA:146<br>Pi-stacking: PHE:28
Danshensu	HRAS	1p2s	-6.8	0.8	Hbond: GLY:13;VAL:14;GLY:15;LYS:16;LYS:16;SER:17;GLU:31;GLU:31;GLU:31;GLY:60<br>Hydrophobic: ALA:18<br>Salt bridge: LYS:16
Docosane	HRAS	1p2s	-5.1	7.6	Hydrophobic: VAL:29;LYS:117;ALA:146;LYS:147
Grandifloroside	HRAS	1p2s	-9.3	1.1	Hbond: GLY:12;SER:17;THR:35;THR:58;GLY:60;GLY:60;ASN:116;LYS:117;ASP:119;ALA:146;LYS:147<br>Hydrophobic: ALA:18<br>Pi-stacking: PHE:28<br>Salt bridge: LYS:16<br>Pi-cation: LYS:117
Hexahydrofarnesylacetone	HRAS	1p2s	-5.5	0.1	Hbond: ASN:116;LYS:117;ALA:146;LYS:147<br>Hydrophobic: ALA:18;PHE:28;GLN:61;LYS:117;LYS:147
Lamiidoside	HRAS	1p2s	-10.2	0.3	Hbond: GLY:13;GLY:13;GLY:15;LYS:16;ALA:18;ASP:30;ASP:33;THR:35;GLY:60;GLN:61;LYS:117;ALA:146;LYS:147

Martynoside	HRAS	1p2s	-8.9	0.8	Hydrophobic: TYR:32;LYS:117;ALA:146;LYS:147<br>Pi-stacking: PHE:28<br>Salt bridge: LYS:117<br>Hbond: LYS:16;SER:17;GLU:31;THR:35;THR:35;ASN:116;LYS:117;ALA:146;LYS:147<br>Hydrophobic: PRO:34;GLN:61<br>Pi-stacking: PHE:28<br>Salt bridge: LYS:16
Miltirone	HRAS	1p2s	-8.4	0.0	Pi-cation: LYS:117<br>Hbond: SER:17;THR:35;THR:35<br>Hydrophobic: ASP:33;PRO:34;GLN:61
Myristicin	HRAS	1p2s	-5.9	0.0	Hbond: ASN:116;ALA:146;LYS:147<br>Hydrophobic: ALA:18<br>Pi-stacking: PHE:28;PHE:28<br>Pi-cation: LYS:117
Phytol	HRAS	1p2s	-6.2	0.4	Hbond: ASN:116;LYS:117;ALA:146;LYS:147<br>Hydrophobic: ALA:18;PHE:28;VAL:29;PRO:34;PRO:34;GLN:61;LYS:117
Piperitenone oxide	HRAS	1p2s	-6.4	0.0	Hbond: GLY:13;LYS:16;SER:17;THR:35;GLY:60<br>Hydrophobic: GLN:61
Przewalskinic acid A	HRAS	1p2s	-8.3	0.6	Hbond: GLY:15;LYS:16;SER:17;ALA:18;ASP:33;THR:35;THR:35;THR:35;GLY:60;GLN:61;GLN:61<br>Hydrophobic: ALA:18;PRO:34

Rosmarinic acid	HRAS	1p2s	-9.3	1.0	Hbond: GLY:13;VAL:14;GLY:15;LYS:16;LYS:16;ASP:33;GLY:60;ASN:116;LYS:117;ASP:119;ALA:146;LYS:147<br>Hydrophobic: ALA:18;ALA:146<br>Pi-stacking: PHE:28
Sagerinic acid	HRAS	1p2s	-6.5	0.2	Hbond: GLY:13;SER:17;ASP:33;THR:35;THR:35
Sahandinone	HRAS	1p2s	-7.4	0.0	Hbond: ASP:33<br>Hydrophobic: PHE:28;PHE:28;TYR:32;LYS:117;ALA:146
Salvianolic acid K	HRAS	1p2s	-9.9	0.1	Hbond: ASP:30;ASP:33;THR:35;THR:35;GLY:60;GLN:61;LYS:117;ASP:119;ALA:146;LYS:147<br>Hydrophobic: ALA:18;TYR:32;ALA:146<br>Pi-stacking: PHE:28<br>Salt bridge: LYS:16
Taxodione	HRAS	1p2s	-8.0	8.6	Hbond: LYS:16;THR:35;GLY:60;GLN:61<br>Hydrophobic: LYS:16;GLN:61
Viridone	HRAS	1p2s	-7.9	0.7	Hbond: LYS:16;THR:35;THR:35;GLY:60;GLN:61;GLN:61<br>Hydrophobic: LYS:16;GLN:61
Yunnaneic acid D	HRAS	1p2s	-9.8	1.0	Hbond: GLY:13;GLY:15;LYS:16;SER:17;ASP:30;ASP:30;THR:58;GLY:60;ASN:116;ASP:119<br>Hydrophobic: TYR:32;PRO:34;PRO:34<br>Pi-stacking: PHE:28

3,5-dicaffeoylquinic acid	NSD2	6XCG	-9.4	1.1	Salt bridge: LYS:16;LYS:16;LYS:117<br>Pi-cation: LYS:117<br>Hbond: PHE:266;ALA:270;GLU:272;GLU:272;PHE:277;SER:280;SER:280<br>Hydrophobic: VAL:230;TYR:233;PHE:266;PHE:266;PHE:266;GLU:272;ILE:276<br>Pi-stacking: TRP:236;TRP:236
4,5-dicaffeoylquinic acid	NSD2	6XCG	-9.0	2.3	Hbond: LYS:229;TYR:233;ALA:270;GLU:272;GLU:272;PHE:277;SER:280;GLN:321<br>Hydrophobic: VAL:230;VAL:230;TYR:233;TRP:236;TRP:236;PHE:266;PHE:266;PHE:266;GLU:272;GLU:272;ILE:276<br>Pi-stacking: TRP:236
6-Hydroxy-5,6-dehydrosugiol	NSD2	6XCG	-7.4	0.9	Hbond: GLU:287<br>Hydrophobic: PRO:234;PHE:290;GLU:291;ARG:319;TRP:322;TRP:322<br>Pi-stacking: PHE:290
8,13-Epoxy-15,16-dinorlabd-12-ene	NSD2	6XCG	-8.7	16.7	Hbond: GLU:272<br>Hydrophobic: TYR:233;TYR:233;TRP:236;TRP:236;PHE:266;PHE:266;ALA:270;GLU:272;LEU:318
Caffeic acid	NSD2	6XCG	-8.0	0.4	Hbond: GLU:272<br>Hydrophobic: TYR:233;TRP:236;PHE:266;GLU:272;GLU:272<br>Pi-stacking: TRP:236;PHE:266

Danshensu	NSD2	6XCG	-7.7	0.6	Hbond: TRP:236;ALA:270<br>Hydrophobic: TYR:233;TRP:236;GLU:272<br>Pi-stacking: TRP:236;TRP:236;PHE:266
Docosane	NSD2	6XCG	-6.0	4.6	Hydrophobic: VAL:230;TYR:233;TYR:233;TYR:233;TYR:233;TRP:236;TRP:236;TRP:236;TRP:236;PHE:266;PHE:266;PHE:266;ALA:270;GLU:272;ALA:274;ILE:276;LEU:318
Grandifloroside	NSD2	6XCG	-7.4	14.0	Hbond: SER:231;TRP:236;SER:280<br>Hydrophobic: VAL:230;VAL:230;PHE:266;GLU:272;ILE:276;PHE:277<br>Pi-stacking: PHE:266
Hexahydrofarnesylacetone	NSD2	6XCG	-6.1	9.9	Hbond: TRP:275<br>Hydrophobic: VAL:230;VAL:230;VAL:230;TYR:233;TRP:236;TRP:236;TRP:236;PHE:266;PHE:266;PHE:266;PHE:266;ALA:270;ALA:274;TRP:275;GLN:321
Lamiidoside	NSD2	6XCG	-8.3	1.1	Hbond: GLU:272;ARG:273;ARG:273;ARG:273;ARG:273;ALA:274;TRP:275<br>Hydrophobic: VAL:230;TYR:233;GLU:272<br>Pi-stacking: PHE:266<br>Salt bridge: ARG:273;ARG:273;ARG:273<br>Pi-cation: ARG:273
Martynoside	NSD2	6XCG	-8.3	0.9	Hbond: LYS:253;GLU:272;ARG:273;TRP

					:275;TRP:275<br>Hydrophobic:<br>LEU:246;PHE:266;GLU:272<br>Pi-stacking: PHE:266<br>Salt bridge: ARG:273
Miltirone	NSD2	6XCG	-8.4	5.7	Hydrophobic:<br>VAL:230;TYR:233;TYR:233;TRP<br>:236;TRP:236;PHE:266;PHE:266;<br>PHE:266;PHE:266;ALA:274<br>Pi-stacking: PHE:266
Myristicin	NSD2	6XCG	-5.8	1.9	Hbond: GLU:272<br>Hydrophobic:<br>TYR:233;TYR:233;TYR:233;TYR<br>:233;TRP:236;TRP:236;PHE:266
Phytol	NSD2	6XCG	-7.1	0.6	Hbond: GLU:272<br>Hydrophobic:<br>VAL:230;VAL:230;VAL:230;TY<br>R:233;TYR:233;TRP:236;TRP:23<br>6;TRP:236;TRP:236;PHE:266;PH<br>E:266;PHE:266;GLU:272;ILE:276<br>;ILE:276;GLN:321
Piperitenone oxide	NSD2	6XCG	-6.8	1.8	Hydrophobic:<br>VAL:230;VAL:230;TYR:233;TY<br>R:233;TYR:233;PHE:266
Przewalskinic acid A	NSD2	6XCG	-8.5	0.9	Hbond:<br>PHE:266;GLU:272;GLN:321<br>Hydrophobic:<br>TRP:236;TRP:236;PHE:266;GLU:<br>272<br>Pi-stacking: PHE:266
Rosmarinic acid	NSD2	6XCG	-7.5	8.2	Hbond:<br>PHE:266;GLU:272;SER:315;SER:<br>315<br>Hydrophobic:<br>PHE:266;LYS:317;LEU:318;LEU:<br>318;LEU:318

					Pi-stacking: TRP:236;TRP:236
Sagerinic acid	NSD2	6XCG	-4.8	15.6	Hbond: LYS:229;LYS:229
Sahandinone	NSD2	6XCG	-8.2	0.0	Hydrophobic: VAL:230;VAL:230;TYR:233;TYR:233;PHE:266;PHE:266;ALA:270;LEU:318<br>Pi-stacking: TYR:233;TRP:236
Salvianolic acid K	NSD2	6XCG	-8.6	0.9	Hbond: GLN:261;GLU:272;GLU:272;GLU:272;SER:280;SER:280<br>Hydrophobic: VAL:230;TYR:233;TRP:236;PHE:266;PHE:266;GLU:272;ALA:274;ILE:276;PHE:277;PHE:277;LEU:318<br>Pi-stacking: TRP:236<br>Salt bridge: LYS:253
Taxodione	NSD2	6XCG	-8.5	0.1	Hbond: GLU:272<br>Hydrophobic: VAL:230;TYR:233;TYR:233;TRP:236;TRP:236;PHE:266;PHE:266;PHE:266;ALA:274<br>Pi-stacking: PHE:266
Viridone	NSD2	6XCG	-8.1	1.0	Hbond: GLU:272<br>Hydrophobic: TYR:233;TRP:236;TRP:236;PHE:266;PHE:266;PHE:266
Yunnaneic acid D	NSD2	6XCG	-8.2	7.3	Hbond: TYR:233<br>Hydrophobic: VAL:230;TYR:233;TYR:233;GLU:272;GLU:272;ARG:273;ARG:273;ALA:274;PHE:346<br>Pi-stacking: PHE:266<br>Salt bridge: ARG:273
3,5-	PPARg	7e0a	-9.5	0.2	Hbond:

dicaffeoylquinic acid					LYS:263;LYS:263;LYS:263;ILE:281;ARG:288;GLU:291;GLN:294;GLN:345<br>Hydrophobic: ARG:288;GLU:291
4,5-dicaffeoylquinic acid	PPARg	7e0a	-9.9	17.2	Hbond: ARG:288;ARG:288;TYR:327;GLU:343;HIS:449<br>Hydrophobic: PHE:264;LYS:265;ILE:281;ARG:288;ILE:326;ILE:341<br>Salt bridge: HIS:266
6-Hydroxy-5,6-dehydrosugiol	PPARg	7e0a	-7.1	24.3	Hydrophobic: LEU:453;GLN:454;LYS:457;LYS:457;GLN:470
8,13-Epoxy-15,16-dinorlabd-12-ene	PPARg	7e0a	-7.2	0.0	Hydrophobic: VAL:293;VAL:315;LEU:318;LEU:318;LYS:319;VAL:322;LEU:468
Caffeic acid	PPARg	7e0a	-7.0	3.8	Hbond: GLU:343;GLU:343<br>Hydrophobic: PHE:264;LYS:265<br>Salt bridge: HIS:266
Danshensu	PPARg	7e0a	-7.4	0.9	Hbond: LEU:340;SER:342;SER:342;GLU:343;GLU:343;GLU:343<br>Hydrophobic: ARG:288;ARG:288;ILE:341<br>Salt bridge: ARG:288
Docosane	PPARg	7e0a	-6.6	7.8	Hydrophobic: PHE:264;PHE:264;LYS:265;ARG:288;ARG:288;ARG:288;LEU:330;LEU:333;GLU:343
Grandifloroside	PPARg	7e0a	-7.3	7.3	Hbond: LYS:224;LYS:263;LYS:263;SER:342;GLN:345;GLN:345<br>Hydrophobic: PRO:227;GLU:295

					Salt bridge: LYS:265
Hexahydrofarnesylacetone	PPARg	7e0a	-6.1	10.8	Hydrophobic: PHE:264;LYS:265;ARG:288;ARG:288;TYR:327;VAL:339;ILE:341
Lamiidoside	PPARg	7e0a	-8.1	1.0	Hbond: LYS:263;ARG:288;ARG:288;GLN:294;GLU:343<br>Hydrophobic: PHE:264;LYS:265;ARG:288
Martynoside	PPARg	7e0a	-8.6	0.5	Hbond: LYS:265;GLU:298;GLU:343;GLU:343;GLU:343<br>Hydrophobic: LYS:265;LYS:265;GLU:291<br>Salt bridge: LYS:265<br>Pi-cation: LYS:265
Miltirone	PPARg	7e0a	-8.6	5.7	Hydrophobic: PHE:264;PHE:264;ARG:288;VAL:339;ILE:341;ILE:341;MET:348
Myristicin	PPARg	7e0a	-6.6	4.3	Hydrophobic: PHE:264;LYS:265;ARG:288
Phytol	PPARg	7e0a	-6.0	9.6	Hydrophobic: PHE:264;PHE:264;LYS:265;PHE:287;GLU:291;ILE:341
Piperitenone oxide	PPARg	7e0a	-7.5	0.0	Hydrophobic: PHE:264;LYS:265
Przewalskinic acid A	PPARg	7e0a	-8.4	4.9	Hbond: ARG:288;GLU:343<br>Hydrophobic: PHE:264;LEU:330;LEU:333;VAL:339;ILE:341;ILE:341
Rosmarinic acid	PPARg	7e0a	-9.7	1.0	Hbond: ILE:281;GLU:291;LEU:340;SER:342;SER:342<br>Hydrophobic: PHE:264;LYS:265;ARG:288;LEU:330;ILE:341;ILE:341

					Salt bridge: HIS:266
Sagerinic acid	PPARg	7e0a	-6.3	4.1	
Sahandinone	PPARg	7e0a	-7.6	18.0	Hydrophobic: LEU:228;PHE:264;ARG:288;LEU:330;LEU:333;VAL:339;ILE:341;GLU:343
Salvianolic acid K	PPARg	7e0a	-8.8	8.9	Hbond: LYS:263;LYS:263;GLU:291;GLN:294;GLN:345<br>Hydrophobic: PHE:264;ARG:288;GLN:294;GLU:295<br>Salt bridge: LYS:265<br>Pi-cation: LYS:265
Taxodione	PPARg	7e0a	-8.1	0.8	Hydrophobic: PHE:264;LEU:330;VAL:339;VAL:339;ILE:341;MET:348
Viridone	PPARg	7e0a	-7.6	0.0	Hydrophobic: GLU:291;GLN:294
Yunnaneic acid D	PPARg	7e0a	-8.6	0.7	Hbond: LYS:263;ARG:288;ARG:288;GLN:294;GLU:298;GLN:345<br>Hydrophobic: LYS:265;LYS:265;ARG:288;GLU:291;GLN:294;GLU:295
3,5-dicaffeoylquinic acid	SHP2	3B7O	-7.7	0.5	Hbond: GLU:361;ARG:362;ALA:461;ARG:465;GLN:510<br>Hydrophobic: ILE:282;ALA:461;GLN:506;THR:507<br>Pi-stacking: TYR:279<br>Salt bridge: LYS:366;ARG:465
4,5-dicaffeoylquinic acid	SHP2	3B7O	-7.2	11.4	Hbond: GLY:427;GLY:427;ALA:461;ARG:465;GLN:510

					Hydrophobic: ILE:282;GLN:506;THR:507<br>Pi-stacking: TYR:279<br>Salt bridge: ARG:362;LYS:366;ARG:465
6-Hydroxy-5,6-dehydrosugiol	SHP2	3B7O	-7.2	14.8	Hbond: GLU:313<br>Hydrophobic: PRO:312;PRO:312;ILE:474;ILE:474;ASP:477;ILE:478;ILE:478;LYS:482
8,13-Epoxy-15,16-dinorlabd-12-ene	SHP2	3B7O	-6.4	3.6	Hydrophobic: ILE:494;GLN:495;TYR:511;TYR:511
Caffeic acid	SHP2	3B7O	-5.8	1.0	Hbond: TRP:423;GLY:427;SER:460;SER:460;SER:460;ALA:461<br>Hydrophobic: ARG:465<br>Salt bridge: ARG:465
Danshensu	SHP2	3B7O	-6.4	1.1	Hbond: TRP:423;GLY:427;GLY:427;ALA:461;GLN:510<br>Salt bridge: ARG:465
Docosane	SHP2	3B7O	-4.4	22.8	Hydrophobic: PHE:247;PHE:247;TRP:248;TRP:248;TRP:248;GLU:250;PHE:251;PHE:251;LEU:254
Grandifloroside	SHP2	3B7O	-6.9	7.1	Hbond: ARG:278;TYR:279;SER:365;LYS:366;TRP:423;SER:460;ARG:465<br>Hydrophobic: TYR:279<br>Pi-stacking: TYR:279<br>Salt bridge: ARG:362;LYS:364;LYS:364;LYS:366;LYS:366;LYS:366;ARG:465<br>Pi-cation: LYS:366;ARG:465

Hexahydrofarnesylacetone	SHP2	3B7O	-4.5	22.7	Hbond: LYS:325<br>Hydrophobic: PRO:312;PRO:312;ILE:478;ILE:478;LYS:492;LYS:492;MET:496
Lamiidoside	SHP2	3B7O	-8.1	2.9	Hbond: ARG:362;TRP:423;GLY:427;ALA:461;GLY:462;ILE:463;GLY:464;ARG:465;ARG:465;GLN:510;GLN:510<br>Hydrophobic: ILE:282;ALA:461<br>Pi-stacking: TYR:279<br>Salt bridge: ARG:362;LYS:364;LYS:366;ARG:465
Martynoside	SHP2	3B7O	-7.5	26.1	Hbond: TYR:279;SER:365;LYS:366;ASP:425;SER:460;GLY:464;ARG:465;ARG:465;ARG:465;GLN:506;GLN:506;GLN:506;GLN:510<br>Hydrophobic: ARG:278;TYR:279;LYS:364<br>Salt bridge: LYS:364;LYS:366;LYS:366
Miltirone	SHP2	3B7O	-6.8	0.0	Hbond: TYR:511<br>Hydrophobic: ILE:494;VAL:505;TYR:511;TYR:511;TYR:511<br>Pi-stacking: TYR:511;TYR:511
Myristicin	SHP2	3B7O	-5.2	2.5	Hbond: LYS:366;ARG:465<br>Hydrophobic: THR:507<br>Pi-cation: ARG:465;ARG:465
Phytol	SHP2	3B7O	-5.3	5.1	Hydrophobic: VAL:505;GLU:508;TYR:511;TYR:511;TYR:511;TYR:511;TYR:511;ARG:512;TYR:515;TYR:515;TYR:515;MET:516

Piperitenone oxide	SHP2	3B7O	-5.6	0.3	Hydrophobic: PRO:491;VAL:505;TYR:511;TYR:511;TYR:511
Przewalskinic acid A	SHP2	3B7O	-7.3	1.0	Hbond: ARG:362;LYS:364;ASP:425;SER:460;ALA:461;GLY:462;ILE:463;GLY:464<br>Hydrophobic: ARG:362;LYS:364<br>Salt bridge: LYS:366<br>Pi-cation: ARG:362;ARG:465
Rosmarinic acid	SHP2	3B7O	-7.1	0.9	Hbond: TRP:423;GLY:427;SER:460;ALA:461;GLN:506<br>Hydrophobic: TYR:279<br>Salt bridge: ARG:362;LYS:366;ARG:465;ARG:465
Sagerinic acid	SHP2	3B7O	-5.8	3.0	Hbond: SER:460;ARG:465<br>Pi-cation: ARG:465
Sahandinone	SHP2	3B7O	-6.1	23.1	Hbond: ALA:461<br>Hydrophobic: TYR:279;ILE:282;ALA:461;ARG:465;THR:507
Salvianolic acid K	SHP2	3B7O	-7.4	0.5	Hbond: ASN:281;LYS:366;TRP:423;GLY:427;SER:460;SER:460;ALA:461;ARG:465;GLN:510<br>Hydrophobic: ARG:465;ARG:465;THR:507<br>Pi-cation: ARG:465
Taxodione	SHP2	3B7O	-6.9	0.9	Hbond: GLY:427;ARG:465<br>Hydrophobic: THR:507
Viridone	SHP2	3B7O	-6.9	0.9	Hbond: TYR:511<br>Hydrophobic: ILE:494;VAL:505;TYR:511;TYR:511;TYR:511

Yunnaneic acid D	SHP2	3B7O	-7.2	27.2	Hbond: GLN:256;LYS:260;TYR:263;SER:499;GLN:500;GLN:500 Hydrophobic: GLN:256;TYR:263;GLN:495;MET:496

Compound	Target	PDB ID	Binding energy (kcal/mol)	RMSD (Å)
3,5-dicaffeoylquinic acid	pUL15-CTD	4IOX	-7.2	5.6
4,5-dicaffeoylquinic acid	pUL15-CTD	4IOX	-7.2	1.1
6-Hydroxy-5,6-dehydrosugiol	pUL15-CTD	4IOX	-6.9	0.8
8,13-Epoxy-15,16-dinorlabd-12-ene	pUL15-CTD	4IOX	-6.7	0.0
Caffeic acid	pUL15-CTD	4IOX	-4.9	0.9
Danshensu	pUL15-CTD	4IOX	-5.4	29.8
Docosane	pUL15-CTD	4IOX	-3.7	29.6
Grandifloroside	pUL15-CTD	4IOX	-6.8	27.6
Hexahydrofarnesylacetone	pUL15-CTD	4IOX	-4.5	27.3
Lamiidoside	pUL15-CTD	4IOX	-6.7	31.2
Martynoside	pUL15-CTD	4IOX	-7.2	1.3
Miltirone	pUL15-CTD	4IOX	-7.5	0.0
Myristicin	pUL15-CTD	4IOX	-4.4	3.3
Phytol	pUL15-CTD	4IOX	-4.9	11.7
Piperitenone oxide	pUL15-CTD	4IOX	-4.9	30.4
Przewalskinic acid A	pUL15-CTD	4IOX	-7.2	0.6
Rosmarinic acid	pUL15-CTD	4IOX	-5.9	6.1
Sagerinic acid	pUL15-CTD	4IOX	-4.7	4.7
Sahandinone	pUL15-CTD	4IOX	-6.8	0.1
Salvianolic acid K	pUL15-CTD	4IOX	-7.1	16.9

Taxodione	pUL15-CTD	4IOX	-7.0	1.1
Viridone	pUL15-CTD	4IOX	-7.3	0.7
Yunnaneic acid D	pUL15-CTD	4IOX	-7.0	29.5
3,5-dicaffeoylquinic acid	UL26 proteaz	8BQ1	-7.9	39.7
4,5-dicaffeoylquinic acid	UL26 proteaz	8BQ1	-6.9	43.7
6-Hydroxy-5,6-dehydrosugiol	UL26 proteaz	8BQ1	-7.0	1.7
8,13-Epoxy-15,16-dinorlabd-12-ene	UL26 proteaz	8BQ1	-7.2	0.0
Caffeic acid	UL26 proteaz	8BQ1	-5.4	1.5
Danshensu	UL26 proteaz	8BQ1	-5.5	0.9
Docosane	UL26 proteaz	8BQ1	-4.0	5.6
Grandifloroside	UL26 proteaz	8BQ1	-7.1	0.5
Hexahydrofarnesylacetone	UL26 proteaz	8BQ1	-4.9	0.1
Lamiidoside	UL26 proteaz	8BQ1	-7.5	1.5
Martynoside	UL26 proteaz	8BQ1	-7.0	1.0
Miltirone	UL26 proteaz	8BQ1	-7.1	0.0
Myristicin	UL26 proteaz	8BQ1	-5.4	34.6
Phytol	UL26 proteaz	8BQ1	-5.0	8.4
Piperitenone oxide	UL26 proteaz	8BQ1	-5.5	34.8
Przewalskinic acid A	UL26 proteaz	8BQ1	-6.9	0.2
Rosmarinic acid	UL26 proteaz	8BQ1	-6.3	21.7
Sagerinic acid	UL26 proteaz	8BQ1	-5.3	0.1
Sahandinone	UL26 proteaz	8BQ1	-6.6	0.0
Salvianolic acid K	UL26 proteaz	8BQ1	-6.8	19.1
Taxodione	UL26 proteaz	8BQ1	-7.8	0.7
Viridone	UL26 proteaz	8BQ1	-7.0	0.1
Yunnaneic acid D	UL26 proteaz	8BQ1	-7.2	0.6

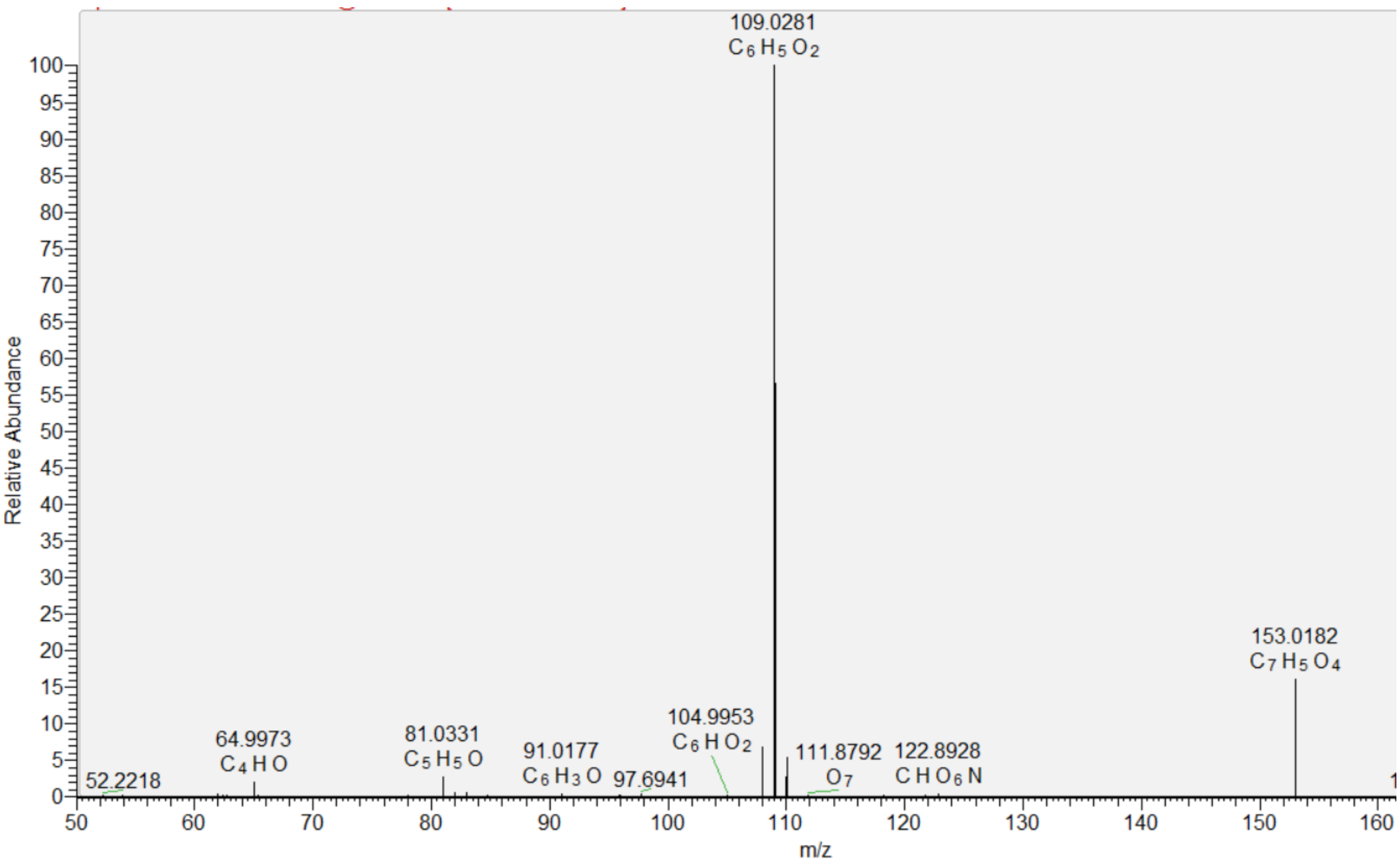

Figure S1. ESI-MS/MS spectrum of protocatechuic acid (2) at *m/z* 153.0193.

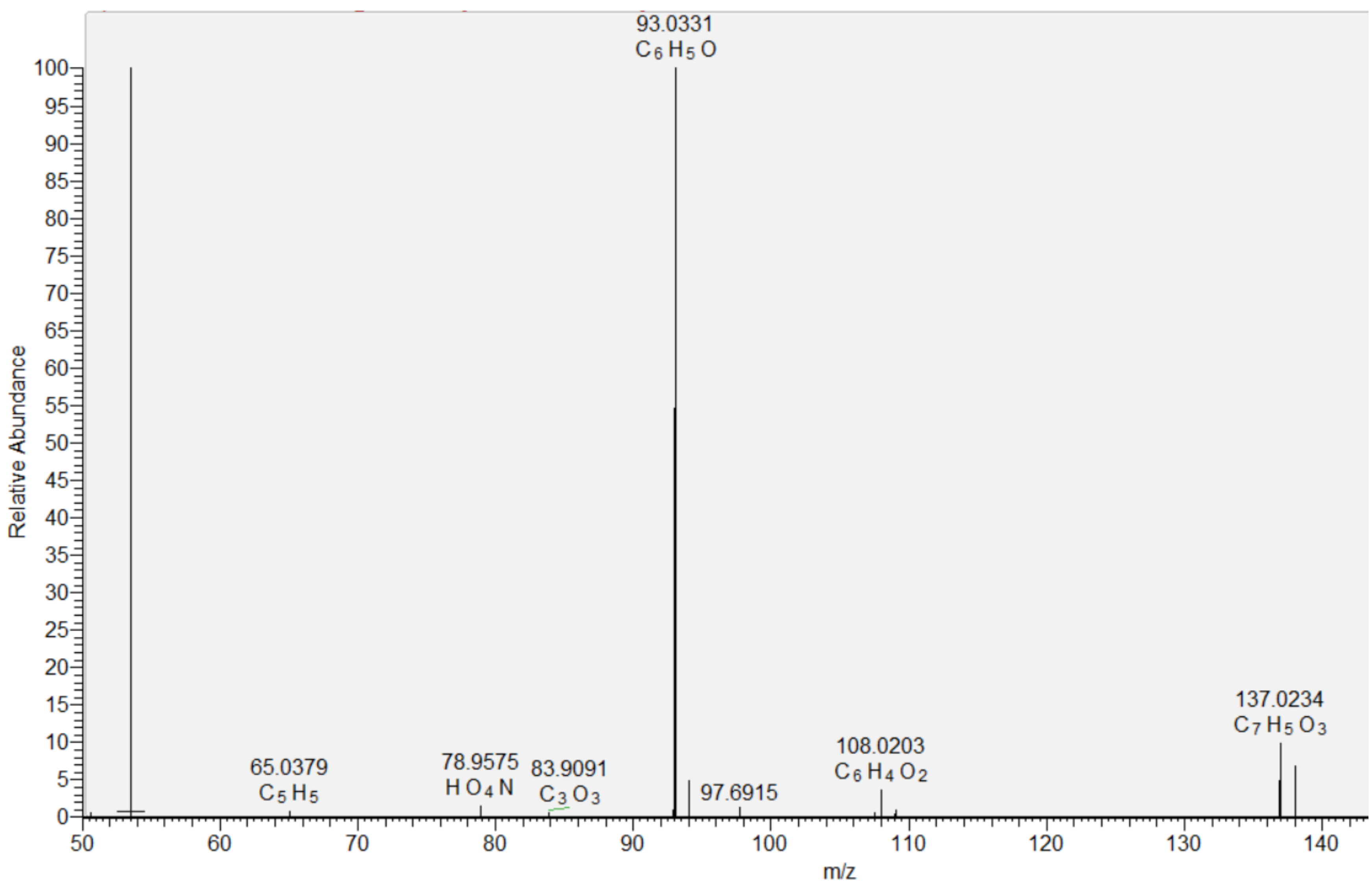

Figure S2. ESI-MS/MS spectrum of p-hydroxybenzoic (6) at *m*/*z* 137.0244.

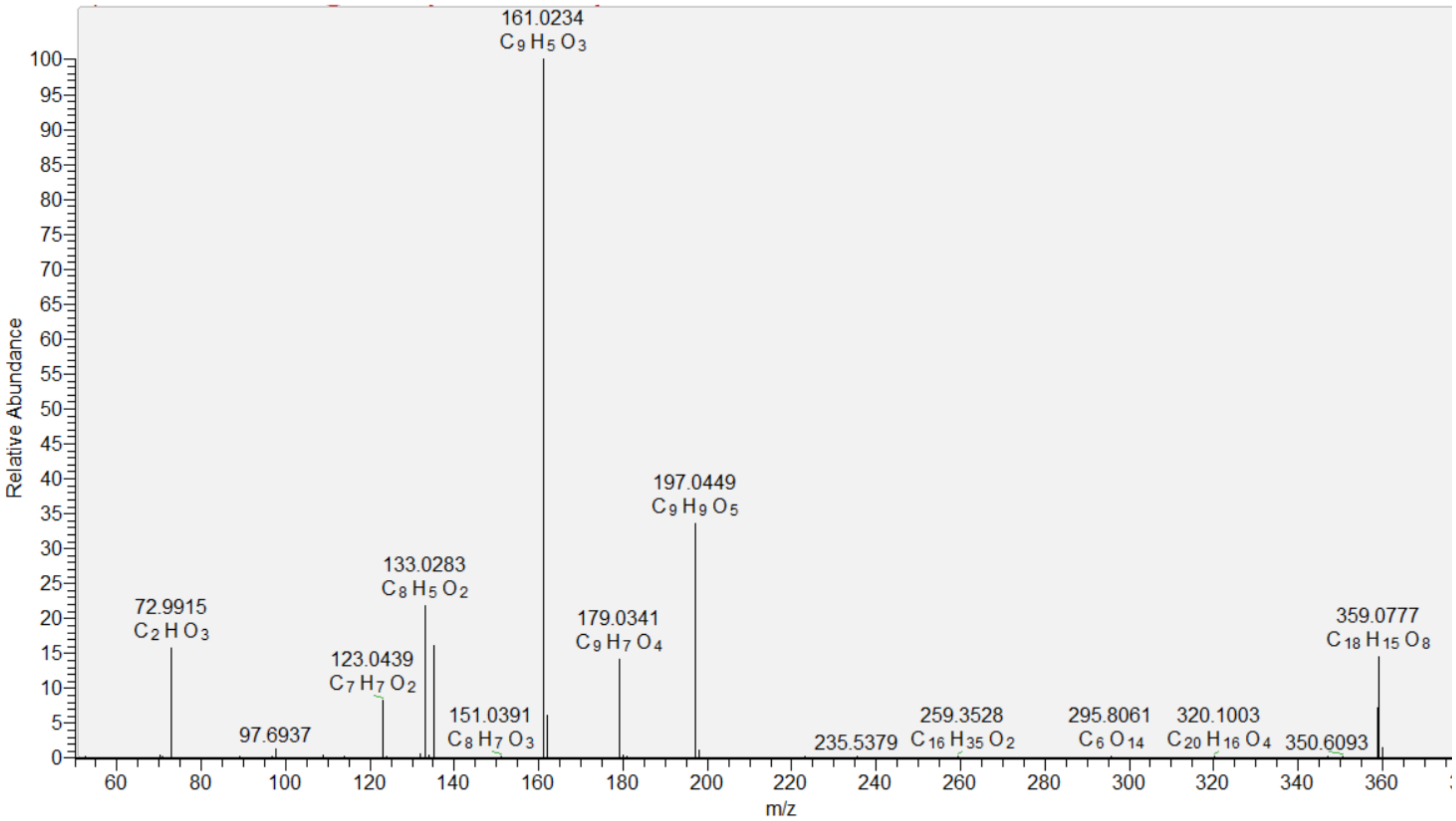

Figure S3. ESI-MS/MS spectrum of chlorogenic acid (9) at *m/z* 353.0878.

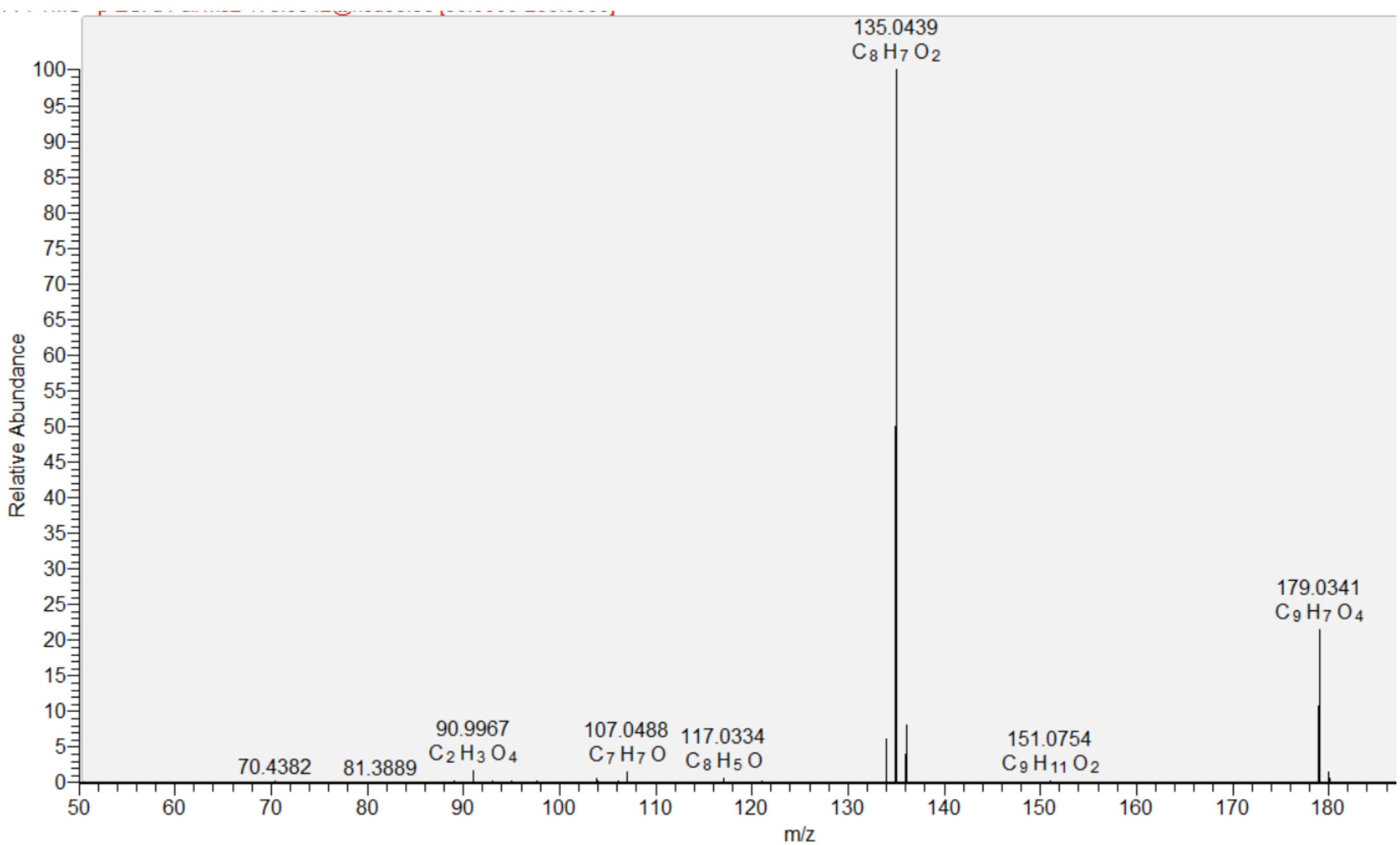

Figure S4. ESI-MS/MS spectrum of caffeic acid (13) at *m/z* 179.0350.

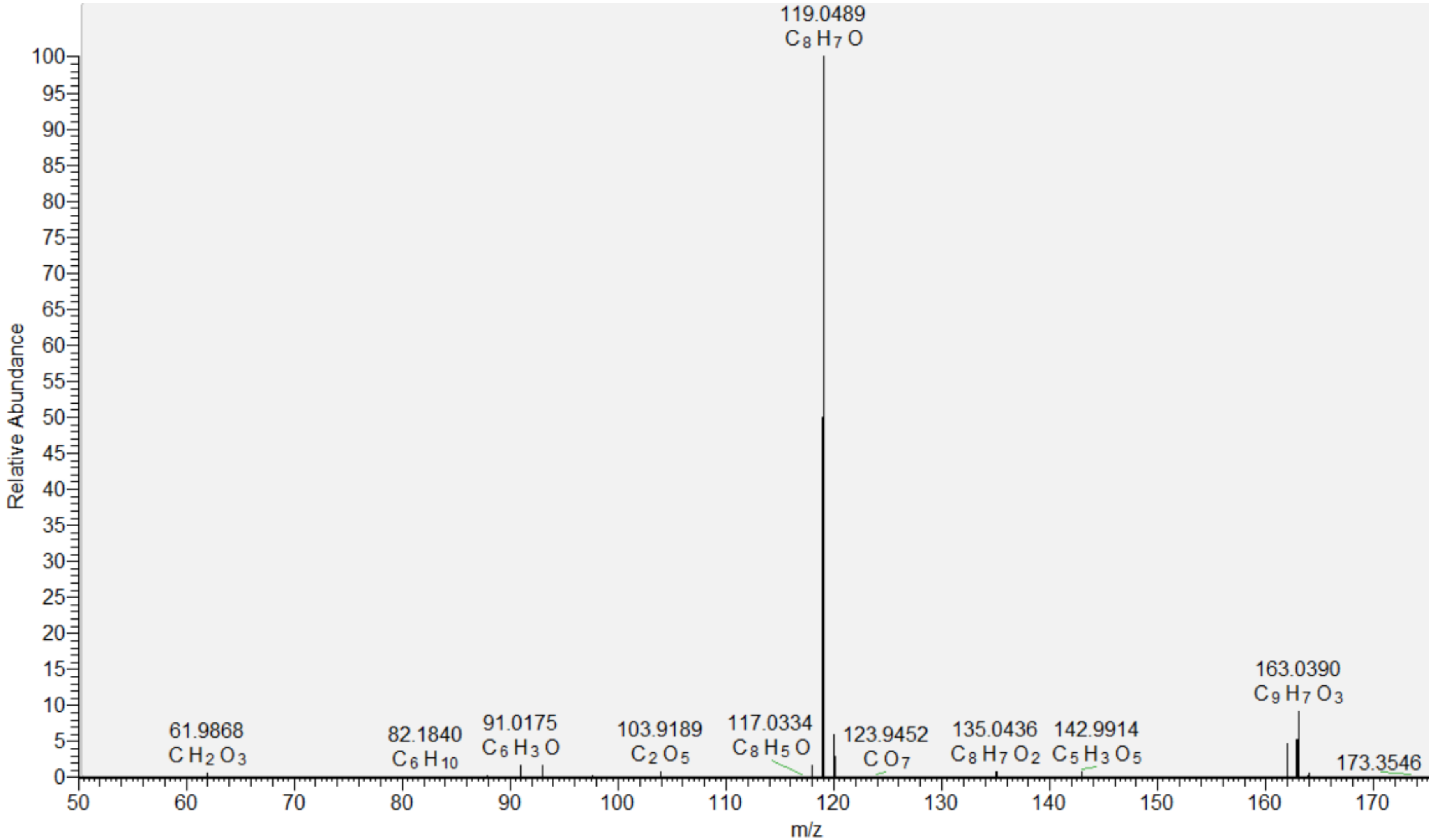

Figure S5. ESI-MS/MS spectrum of *o*-coumaric acid (16) at *m*/*z* 163.0401.

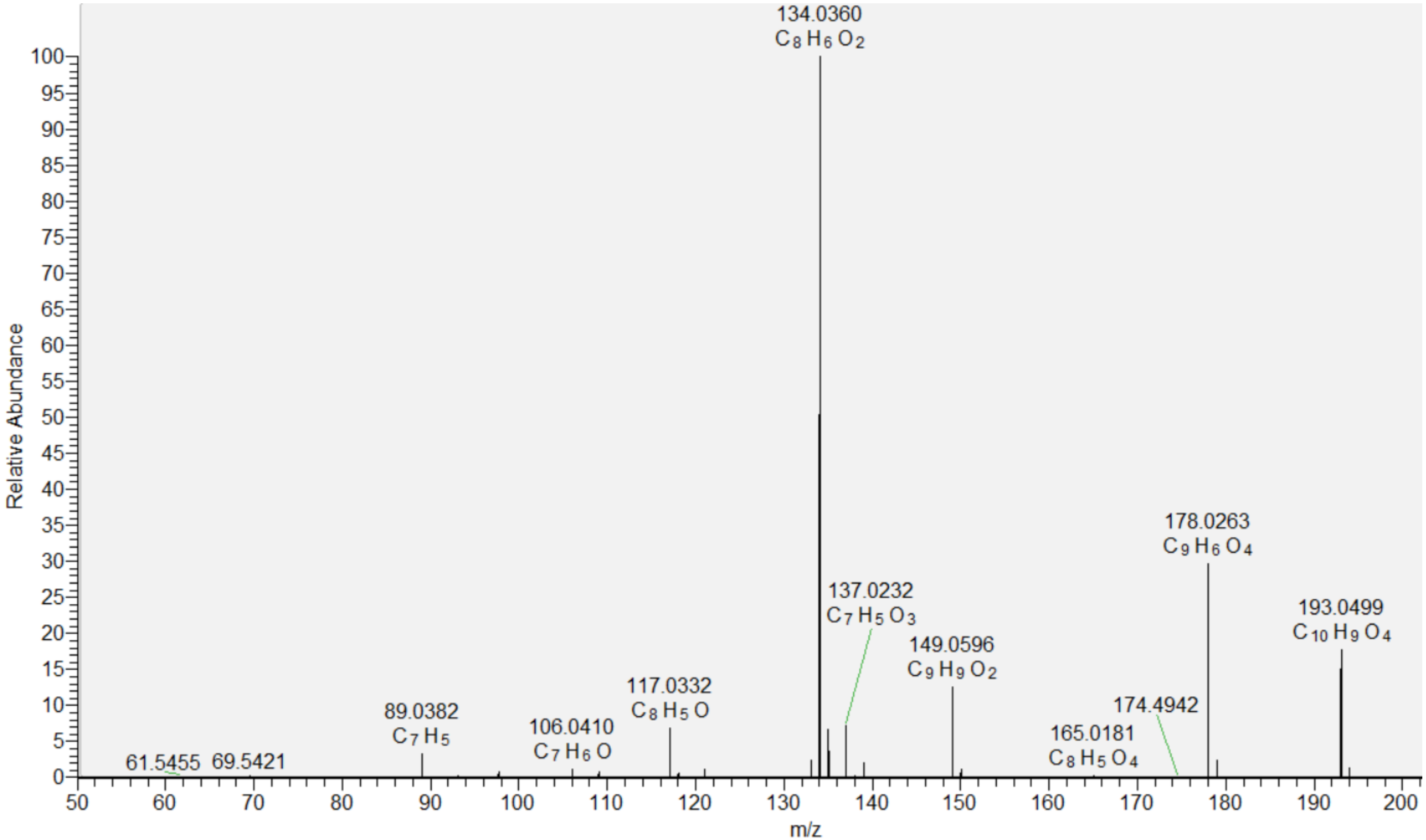

Figure S6. ESI-MS/MS spectrum of ferulic acid (17) at *m*/*z* 193.0506.

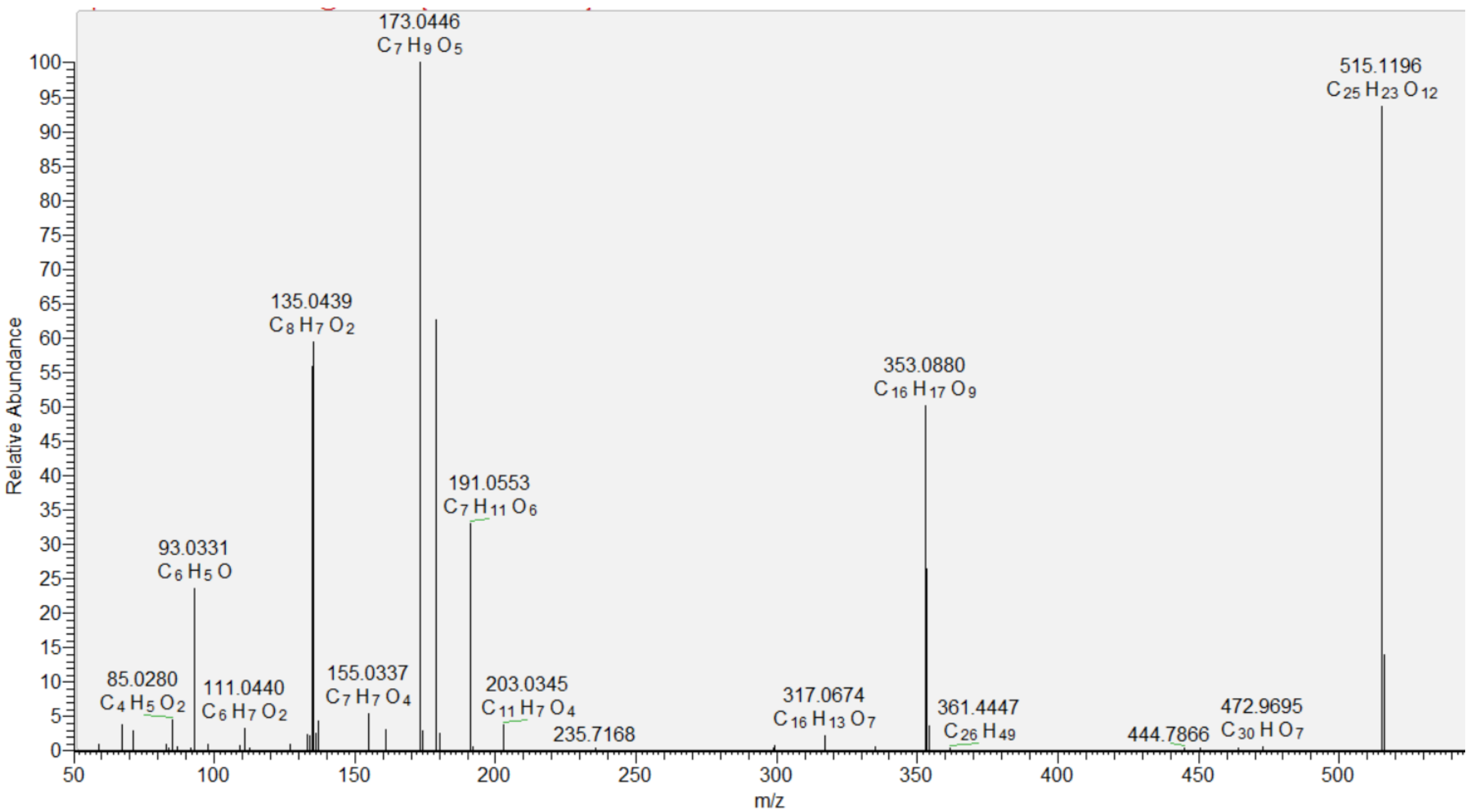

Figure S7. ESI-MS/MS spectrum of 4,5-dicaffeoylquinic acid (19) at *m/z* 515.1195.

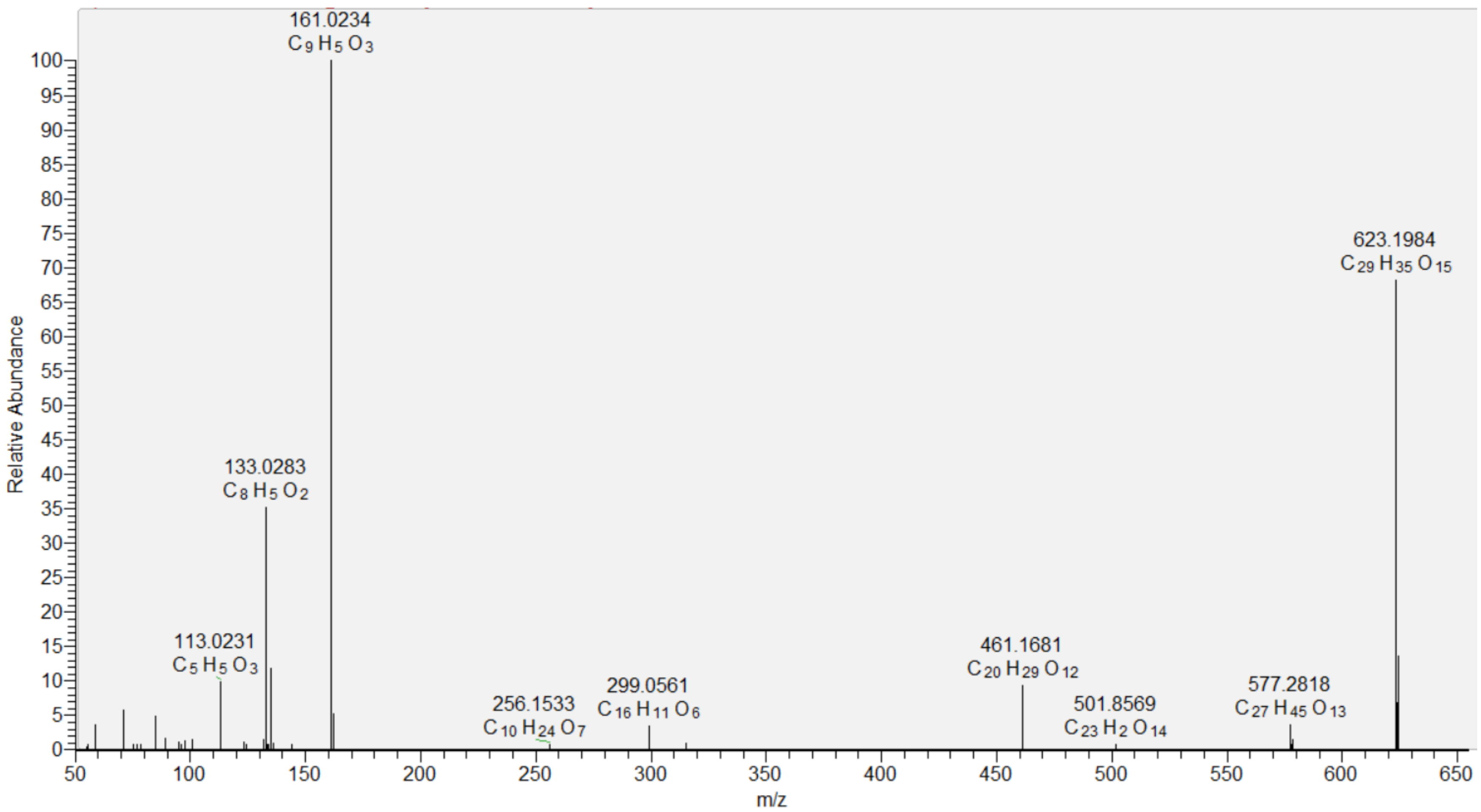

Figure S8. ESI-MS/MS spectrum of verbascoside (29) at *m/z* 623.1981.

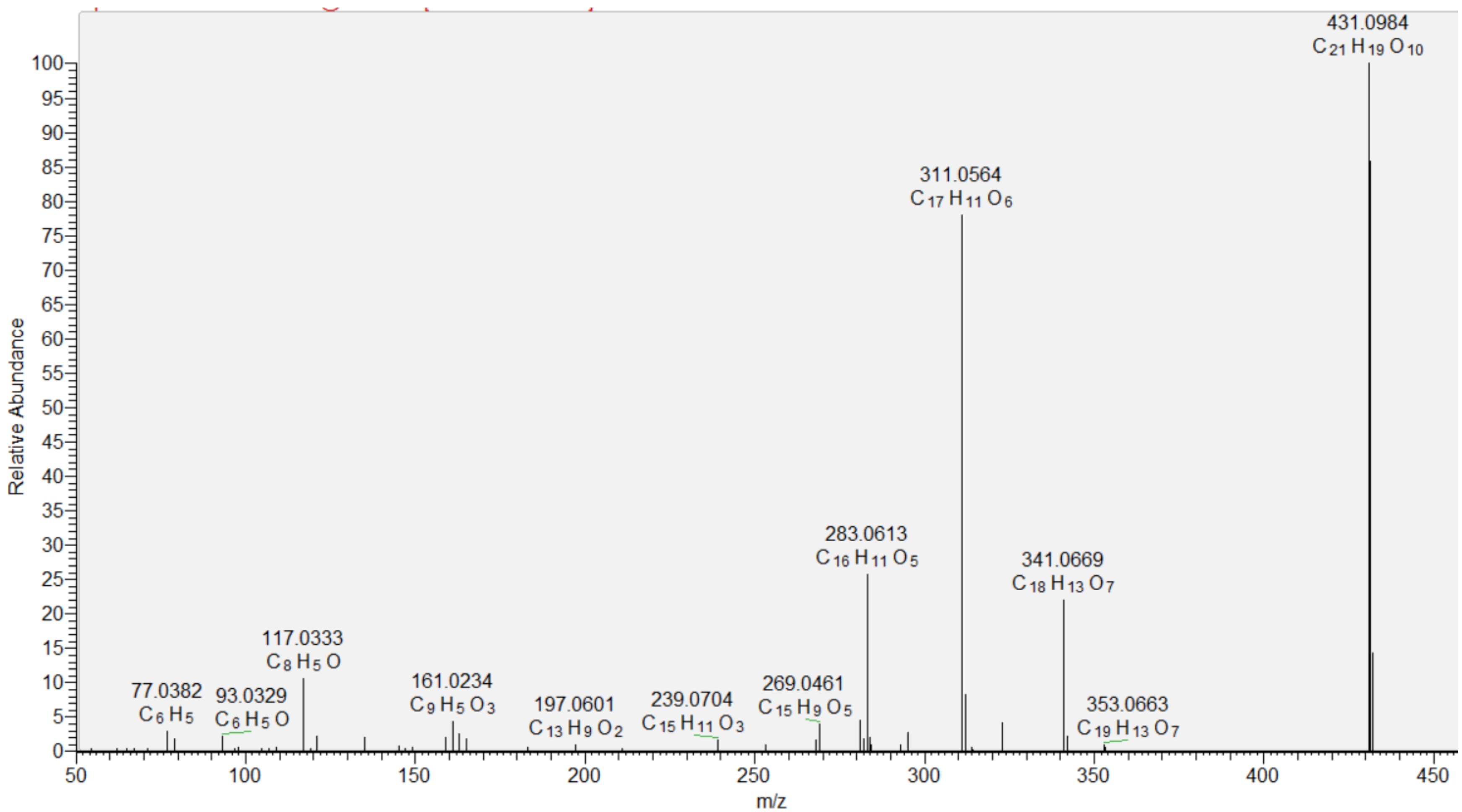

Figure S9. ESI-MS/MS spectrum of isovitexin (41) at *m/z* 431.0984.

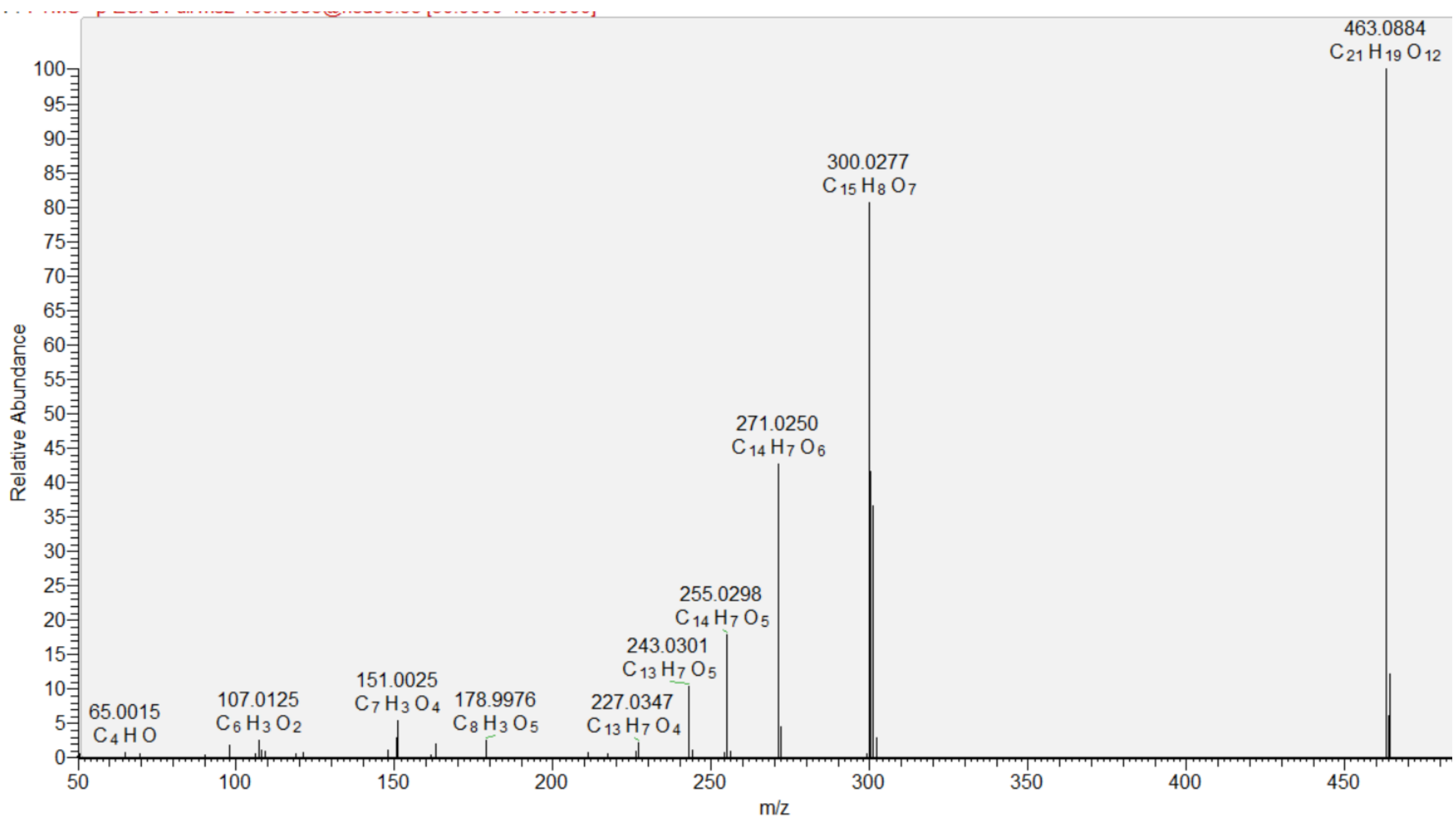

Figure S10. ESI-MS/MS spectrum of hyperoside (42) at *m*/*z* 463.0882.

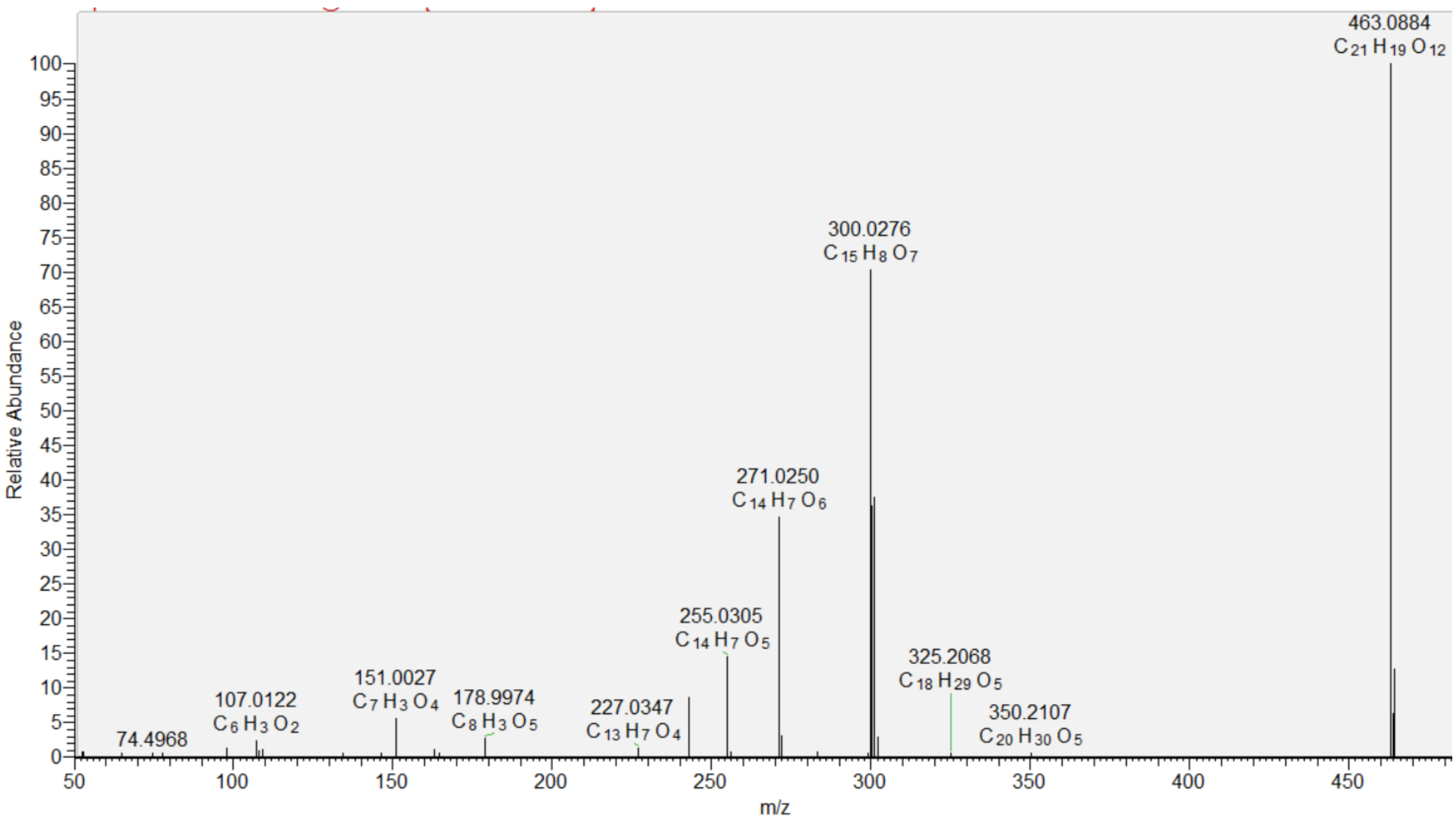

Figure S11. ESI-MS/MS spectrum of isoquercitrin (43) at *m*/*z* 463.0882.

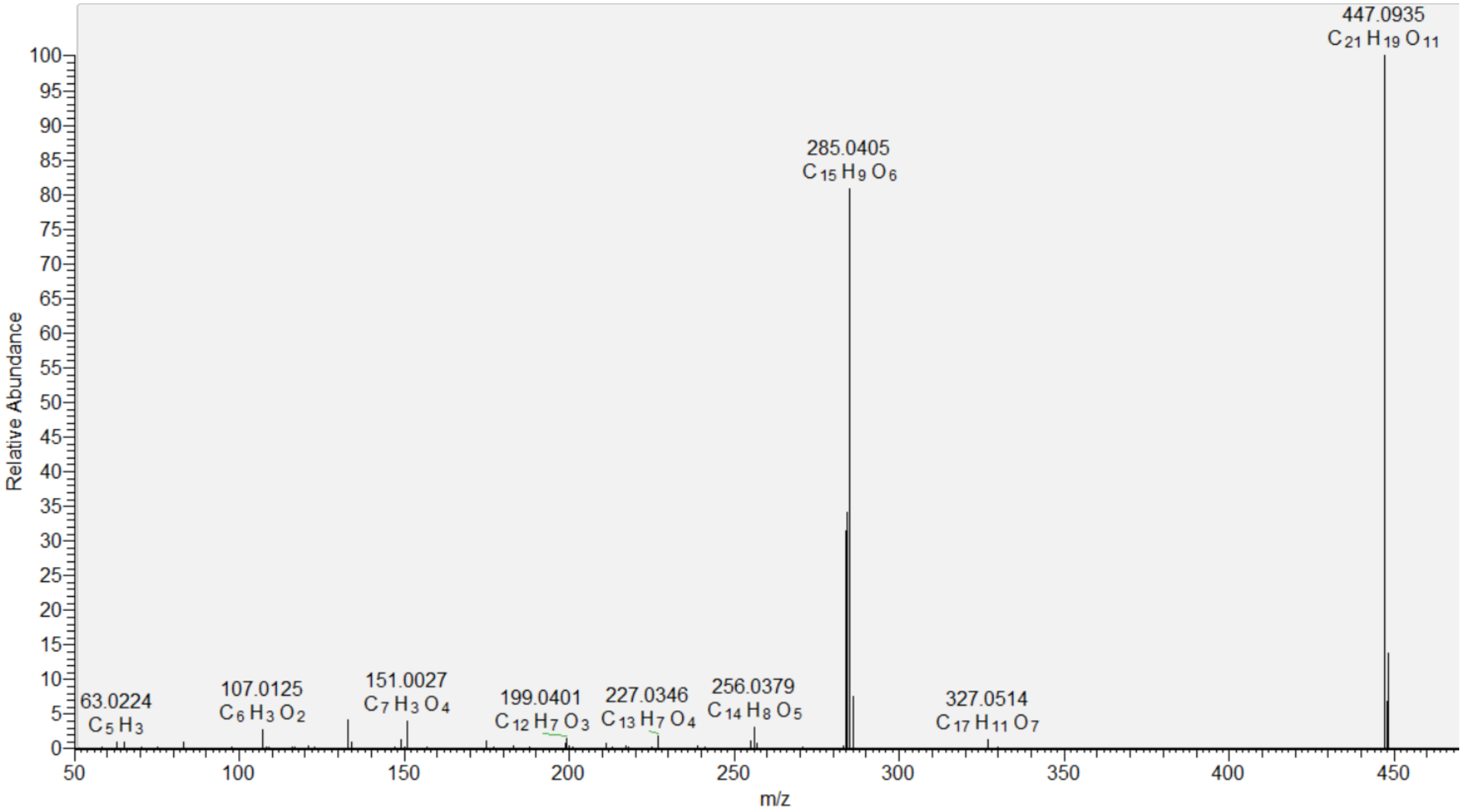

Figure S12. ESI-MS/MS spectrum of luteolin 7-*O*-glucoside (45) at *m/z* 447.0933.

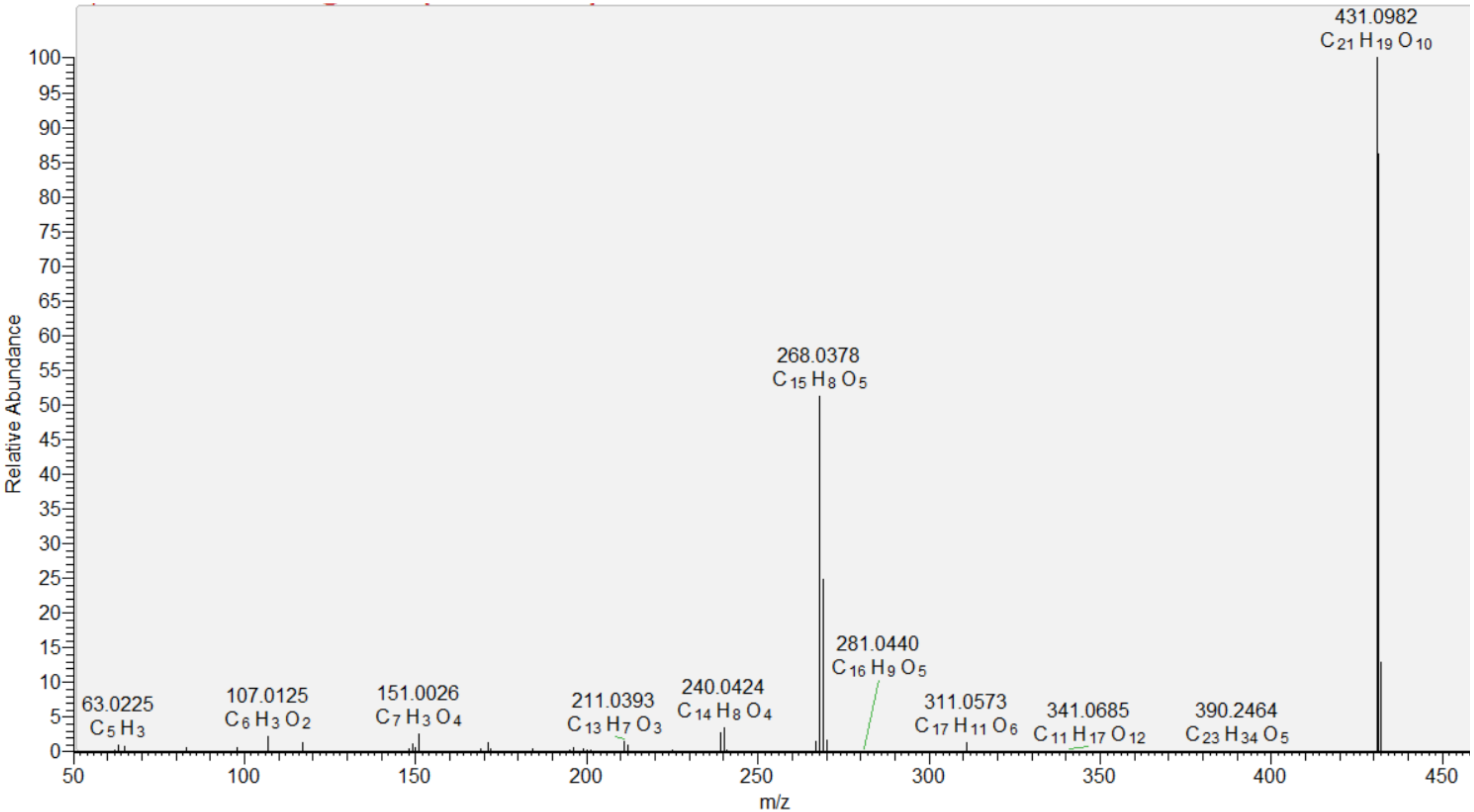

Figure S13. ESI-MS/MS spectrum of apigenin 7-*O*-glucoside (46) at *m*/*z* 431.0984.

References

1. Adams, R.P., *Identification of essential oil components by gas chromatography/mass spectrometry*. 4th ed. 2007, Carol Stream, IL, USA.: Allured Publishing Corp.
